# Association of Cardiometabolic Genes with Arsenic Metabolism Biomarkers in American Indian Communities: The Strong Heart Family Study (SHFS)

**DOI:** 10.1289/EHP251

**Published:** 2016-06-28

**Authors:** Poojitha Balakrishnan, Dhananjay Vaidya, Nora Franceschini, V. Saroja Voruganti, Matthew O. Gribble, Karin Haack, Sandra Laston, Jason G. Umans, Kevin A. Francesconi, Walter Goessler, Kari E. North, Elisa Lee, Joseph Yracheta, Lyle G. Best, Jean W. MacCluer, Jack Kent, Shelley A. Cole, Ana Navas-Acien

**Affiliations:** 1 Department of Environmental Health Sciences, and; 2 Department of Epidemiology, Johns Hopkins University Bloomberg School of Public Health, Baltimore, Maryland, USA; 3 The Welch Center for Prevention, Epidemiology, and Clinical Research, Johns Hopkins Medical Institutions, Baltimore, Maryland, USA; 4 Clinical and Translational Research, Johns Hopkins School of Medicine, Baltimore, Maryland, USA; 5 Department of Epidemiology, and; 6 Department of Nutrition, University of North Carolina at Chapel Hill, Chapel Hill, North Carolina, USA; 7 UNC Nutrition Research Institute, University of North Carolina at Chapel Hill, Kannapolis, North Carolina, USA; 8 Department of Environmental Health, Rollins School of Public Health, Emory University, Atlanta, Georgia, USA; 9 Department of Genetics, Texas Biomedical Research Institute, San Antonio, Texas, USA; 10 South Texas Diabetes and Obesity Institute, University of Texas Rio Grande Valley, Brownsville, Texas, USA; 11 MedStar Health Research Institute, Hyattsville, Maryland, USA; 12 Georgetown and Howard Universities Center for Clinical and Translational Science, Washington, DC, USA; 13 Institute of Chemistry - Analytical Chemistry, University of Graz, Austria; 14 Center for American Indian Health Research, College of Public Health, University of Oklahoma Health Sciences Center, Oklahoma City, Oklahoma, USA; 15 Missouri Breaks Industries Research, Inc., Timber Lake, South Dakota, USA; 16 Department of Oncology, Johns Hopkins School of Medicine, Baltimore, Maryland, USA

## Abstract

**Background:**

Metabolism of inorganic arsenic (iAs) is subject to inter-individual variability, which is explained partly by genetic determinants.

**Objectives:**

We investigated the association of genetic variants with arsenic species and principal components of arsenic species in the Strong Heart Family Study (SHFS).

**Methods:**

We examined variants previously associated with cardiometabolic traits (~ 200,000 from Illumina Cardio MetaboChip) or arsenic metabolism and toxicity (670) among 2,428 American Indian participants in the SHFS. Urine arsenic species were measured by high performance liquid chromatography–inductively coupled plasma mass spectrometry (HPLC-ICP-MS), and percent arsenic species [iAs, monomethylarsonate (MMA), and dimethylarsinate (DMA), divided by their sum × 100] were logit transformed. We created two orthogonal principal components that summarized iAs, MMA, and DMA and were also phenotypes for genetic analyses. Linear regression was performed for each phenotype, dependent on allele dosage of the variant. Models accounted for familial relatedness and were adjusted for age, sex, total arsenic levels, and population stratification. Single nucleotide polymorphism (SNP) associations were stratified by study site and were meta-analyzed. Bonferroni correction was used to account for multiple testing.

**Results:**

Variants at 10q24 were statistically significant for all percent arsenic species and principal components of arsenic species. The index SNP for iAs%, MMA%, and DMA% (rs12768205) and for the principal components (rs3740394, rs3740393) were located near AS3MT, whose gene product catalyzes methylation of iAs to MMA and DMA. Among the candidate arsenic variant associations, functional SNPs in AS3MT and 10q24 were most significant (p < 9.33 × 10–5).

**Conclusions:**

This hypothesis-driven association study supports the role of common variants in arsenic metabolism, particularly AS3MT and 10q24.

**Citation:**

Balakrishnan P, Vaidya D, Franceschini N, Voruganti VS, Gribble MO, Haack K, Laston S, Umans JG, Francesconi KA, Goessler W, North KE, Lee E, Yracheta J, Best LG, MacCluer JW, Kent J Jr., Cole SA, Navas-Acien A. 2017. Association of cardiometabolic genes with arsenic metabolism biomarkers in American Indian communities: the Strong Heart Family Study (SHFS). Environ Health Perspect 125:15–22; http://dx.doi.org/10.1289/EHP251

## Introduction

Inorganic arsenic (iAs) is a toxic and carcinogenic metalloid that is found in groundwater, soil, food, and air (Welch et al. 1988). Experimental and epidemiological studies support the role of chronic iAs exposure in arsenic toxicity (Hughes 2002). In particular, iAs exposure has been associated with cancer [International Agency for Research on Cancer (IARC) 2004], cardiometabolic disease (Moon et al. 2012; Navas-Acien et al. 2008), and kidney disease (Peters et al. 2014; Zheng et al. 2015). After entering the body, iAs undergoes two sets of reduction and methylation reactions to a trivalent state and oxidation to a pentavalent state, producing monomethylarsonate (MMA) and then dimethylarsinate (DMA) (Hughes 2002). The arsenic species iAs, MMA, and DMA excreted via urine serve as biomarkers of arsenic metabolism (Vahter 1999). Although the pathways of arsenic toxicity have not been completely established, trivalent arsenic metabolites have been implicated in arsenic-related toxicity via mechanisms including epigenetic regulation, cytotoxicity, interfering with DNA repair, and oxidative stress (Hughes 2002).

More research is needed to understand the mechanisms of arsenic toxicity, particularly given its systemic physiological effect on multiple organs. Arsenic toxicity may be better understood by investigating differences in response to arsenic; arsenic metabolism and patterns of arsenic methylation are subject to inter-individual variation and may be influenced by genetic susceptibility, age, sex, nutrition, route of exposure, and other risk factors (Concha et al. 2002; Vahter 1999). The proportion of variation explained by genetic determinants (heritability) range from 50% to 53% for iAs%, 16% to 50% for MMA%, and 33% to 63% for DMA% (Chung et al. 2002; Gao et al. 2015). Association studies have shown the involvement of single nucleotide polymorphisms (SNPs) in the arsenic (III) methyltransferase gene *AS3MT*, which encodes a major enzyme in biotransformation of iAs to MMA and DMA (Agusa et al. 2009; Rodrigues et al. 2012; Schläwicke Engström et al. 2009). More recently, genome-wide association studies (GWAS) in Bangladesh showed the association of *AS3MT* variants with MMA% and DMA% (Pierce et al. 2012). Additionally, because kidney disease, type 2 diabetes, and other cardiometabolic diseases have been associated with arsenic exposure, genes associated with metabolic traits may be relevant determinants of arsenic species.

U.S. Environmental Protection Agency (EPA) regulations state a maximum arsenic contaminant level in drinking water of 10 μg/L. In 2001, the U.S. EPA Regional Tribal Program estimated that 16.5% of tribally owned community water systems had at least one source with arsenic levels > 10 μg/L, similar to other rural and suburban communities around the United States, especially in the West, the Midwest, and the Northeast (Navas-Acien et al. 2009). In this study, we characterized the genetic architecture of arsenic metabolism in extended American Indian families from Arizona, Oklahoma, and North and South Dakota. Our objective was to replicate previous studies and to find novel associations of common variants with markers of arsenic metabolism, which can highlight biological pathways that influence arsenic toxicity and related diseases. In addition, because the evaluation of arsenic metabolism is complex—being based on three interrelated biomarkers that sum to 100% (iAs%, MMA%, DMA%)—we used a relatively novel method to summarize inter-individual variability in orthogonal urine arsenic species patterns using principal components analysis (PCA).

## Methods

### Study Population

The Strong Heart Family Study (SHFS; http://strongheart.ouhsc.edu/) is a large, multigenerational cohort recruited from the Strong Heart Study (SHS), an ongoing population-based study conducted in 13 American Indian tribes/communities in Arizona, Oklahoma, and North and South Dakota. Details of the study design have been described previously (Lee et al. 1990; North et al. 2002). Briefly, families were eligible if they had a core sibship consisting of 3 original SHS participants and ≥ 5 additional living family members including 3 original SHS participants. The baseline visit was conducted in two phases; ~1,000 participants had their baseline visit in 1998–1999, and > 2,500 participants had their baseline visit in 2001–2003. Our study was restricted to 2,428 participants who were free of diabetes at the baseline visit, had urine arsenic species measured at baseline, were genotyped (MetaboChip and custom panel), and passed genotyping quality control, which is described in detail below. The SHFS protocols were approved by the Indian Health Service (https://www.ihs.gov) Institutional Review Board, by the Institutional Review Boards of the participating institutions, and by the participating American Indian tribes. Informed consent was obtained from all participants.

### Arsenic Measurements

Total urine arsenic and arsenic species concentrations were measured in spot urine samples collected on the morning of the baseline visit. The samples were frozen and stored at –70°C. Total urine arsenic was determined using inductively coupled plasma mass spectrometry (ICP-MS), and arsenic species concentrations were determined using high performance liquid chromatography–ICP-MS (HPLC-ICP-MS) (Scheer et al. 2012). The inter-batch variability was monitored by obtaining replicate measurements of three urine reference materials with certified arsenic levels of 20.3 μg/L [National Institute of Standards and Technology (NIST) Standard Reference Material (SRM) 2669 I], 50.2 μg/L (NIST SRM 2669 II), or 119 μg/L [National Institute for Environmental Studies (NIES) Certified Reference Material (CRM) 18]; the coefficients of variation (CVs) ranged from 3.8% to 14.4%, with an overall mean CV of 7.9% (*n* = 46) (see Table S1). The limit of quantitation for total arsenic and arsenic species was 0.10 μg/L. A total of 221 samples were below the limit of quantitation for iAs (9.1%), 63 samples were below the limit of quantitation for MMA (2.6%), and 1 sample was below the limit of quantitation for DMA (< 0.1%). The arsenic species levels below the limit of quantitation were imputed as the limit of quantitation divided by the square root of 2 (0.07 μg/L). Because the lab assay included oxidization, the pentavalent and trivalent species were indistinguishable. The percentage of each arsenic species (iAs%, MMA%, DMA%) was calculated as the relative proportion of the species to the sum of all three arsenic species.

### SNP Genotyping

DNA was extracted from blood specimens obtained at the baseline visit using organic solvents (North et al. 2003) and was genotyped according to Illumina protocol (Voight et al. 2012) using the Illumina Cardio-Metabo DNA Analysis BeadChip (MetaboChip), which contains 196,725 markers. These markers were selected based on a large-scale meta-analysis for cardiometabolic traits such as coronary artery disease and type 2 diabetes. Approximately one third of the MetaboChip SNPs consist of replication targets, and nearly two thirds are located in fine mapping regions, including *AS3MT* and other genes in the 10q24 region. Before genotyping quality control, nonautosomal and monomorphic markers were removed. Genotyping inconsistencies (Mendelian errors) were removed using Preswalk, a PEDSYS-compatible (Dyke 1996) version of Simwalk2 (Sobel et al. 2002), and allele frequency estimation and Hardy–Weinberg equilibrium (HWE) were estimated using Sequential Oligogenic Linkage Analysis Routines (SOLAR) (Blangero and Almasy 1996). Family-based imputation was performed using a PEDSYS-compatible version of Merlin (Abecasis et al. 2002). Participants were excluded if the genotyping call rate was < 95% (*n* = 3). The SNP exclusion criteria included a call rate < 98% or no data (*n* = 33,604), not autosomal (*n* = 250), monomorphic (*n* = 158), HWE *p* < 1 × 10^–5^ (*n* = 1,519), and minor allele frequency (MAF) < 0.01 (*n* = 40,219). As a result, there were 120,975 common variants used in the analysis. Pairwise correlations (*r* ^2^) between markers were calculated to estimate linkage disequilibrium (LD). A custom panel was used to genotype loci that were associated with arsenic traits in previous studies that were not already genotyped on the MetaboChip. A total of 670 arsenic candidate SNPs from 55 candidate genes (see Table S2) were genotyped. SNPs were assessed for assay inconsistencies and for whether they were monomorphic in the sample. Samples were also assessed for genotyping errors using a call rate < 95%, mismatch between genotyped and reported sex, outliers in identity by descent (IBD) clustering, or outliers in PCA. There were no SNPs that failed quality control.

### Statistical Analysis

Percent arsenic species were logit transformed to approximate a normal distribution. Because percent arsenic species are interdependent, we also used PCA to summarize orthogonal dimensions of inter-individual variability in urine arsenic species patterns using the covariance structure of the arsenic species. Association analyses of 2,428 participants using MetaboChip SNPs and arsenic candidate SNPs were performed. All traits were modeled using linear regression of allele dosage at each SNP and were adjusted for age at baseline, sex, total arsenic levels, and principal components for population stratification. An additive SNP effect was assumed. All analyses were stratified by study region and accounted for familial relatedness using SOLAR. An inverse-variance-weighted meta-analysis of the stratified associations was performed using METAL (Willer et al. 2010). The MetaboChip-wide significance threshold was adjusted for multiple testing using Bonferroni correction (0.05/120,975 = 4.13 × 10^–7^). Because the Bonferroni method would overcorrect for multiple testing in the presence of LD (Nyholt 2004), the suggestive threshold was calculated for the effective number of SNPs accounting for LD using SOLAR (0.05/64374.85 = 7.77 × 10^–7^). Similarly for the arsenic candidate SNPs, the Bonferroni-corrected significance threshold was 9.33 × 10^–5^, and the LD-corrected significance threshold was 9.1 × 10^–5^. Association analysis conditioned on the index SNP, the most statistically significant, informative SNP at the locus, was also performed using SOLAR. Simple linear regression analysis and stratification by sex were also performed for the arsenic traits as secondary analyses. All descriptive analysis was performed using R (version 3.2.2; R Foundation for Statistical Computing).

## Results

The median [interquartile range (IQR)] for the sum of inorganic and methylated arsenic species was 6.6 (3.9–11.6) μg/L. Urine arsenic concentrations were higher in participants from Arizona than in participants from Oklahoma and North and South Dakota (Table 1). For arsenic metabolism, the median (IQR) was 9.8 (6.4–14.0) for iAs%, 13.9 (10.5–17.7) for MMA%, and 75.6 (68.6–81.6) for DMA%, with some variability across study regions (highest iAs% in Arizona, highest MMA% in North and South Dakota, and highest DMA% in Oklahoma). The variability in iAs%, MMA%, and DMA% can be summarized in two principal components. Principal component 1 (PC1) explained 86.1% of the variance in arsenic species and reflected higher iAs% and MMA% and lower DMA% (Table 2). PC2 explained the remaining 13.9% of variance in arsenic species and reflected higher iAs% and lower MMA% independent of DMA% (Table 2).

**Table 1:** Baseline characteristics of Strong Heart Family Study participants.

Characteristic	Total	Arizona	Oklahoma	North and South Dakota	*p*-Value
Number of participants	2,428	703	819	906
Mean age, years (SD)	35.2 (15.2)	30.9 (13.1)	38.8 (15.7)	35.3 (15.4)	< 0.01
Number of females (%)	1,469 (60.5%)	438 (62.3%)	486 (59.34%)	545 (60.15%)	0.48
Arsenic levels					< 0.01
Tertile 1 (0.21–4.73 μg/L)	804 (33.1%)	100 (14.2%)	357 (43.5%)	347 (38.3%)
Tertile 2 (4.74–9.35 μg/L)	799 (32.9%)	204 (29.0%)	293 (35.7%)	302 (33.3%)
Tertile 3 (9.37–176.6 μg/L)	825 (33.9%)	399 (56.7%)	169 (20.6%)	257 (28.3%)
Median iAs% (IQR)	9.78 (6.4–14.0)	10.8 (7.6–15.2)	8.2 (5.4–12.3)	10.1 (6.7–14.5)	< 0.01
Median MMA% (IQR)	13.9 (10.5–17.7)	12.8 (10.0–16.2)	13.7 (10.3–17.8)	14.9 (11.5–18.5)	< 0.01
Median DMA% (IQR)	75.6 (68.6–81.6)	75.2 (69.0–81.1)	77.2 (70.5–83.3)	74.3 (67.3–80.4)	< 0.01
Mean PC1 (SD)	0.04 (12.21)	0.62 (12.05)	–2.20 (11.64)	1.62 (12.54)	< 0.01
Mean PC2 (SD)	–0.01 (4.91)	1.59 (4.87)	–0.70 (4.53)	–0.63 (5.00)	< 0.01
Notes: DMA%, percent dimethylarsinate; iAs%, percent inorganic arsenic; IQR, interquartile range; MMA%, percent monomethylarsonate; PC, principal component; SD, standard deviation.					

**Table 2:** Summary of principal components of arsenic species.

Descriptive statistic	PC1	PC2
Variance in arsenic species explained (%)	86.1	13.9
Standard deviation	12.21	4.91
Weight for iAs%	0.49	0.65
Weight for MMA%	0.32	–0.75
Weight for DMA%	–0.81	0.10
Notes: DMA%, percent dimethylarsinate; iAs%, percent inorganic arsenic; MMA%, percent monomethyl­arsonate; PC, principal component.		

**Figure 1:**
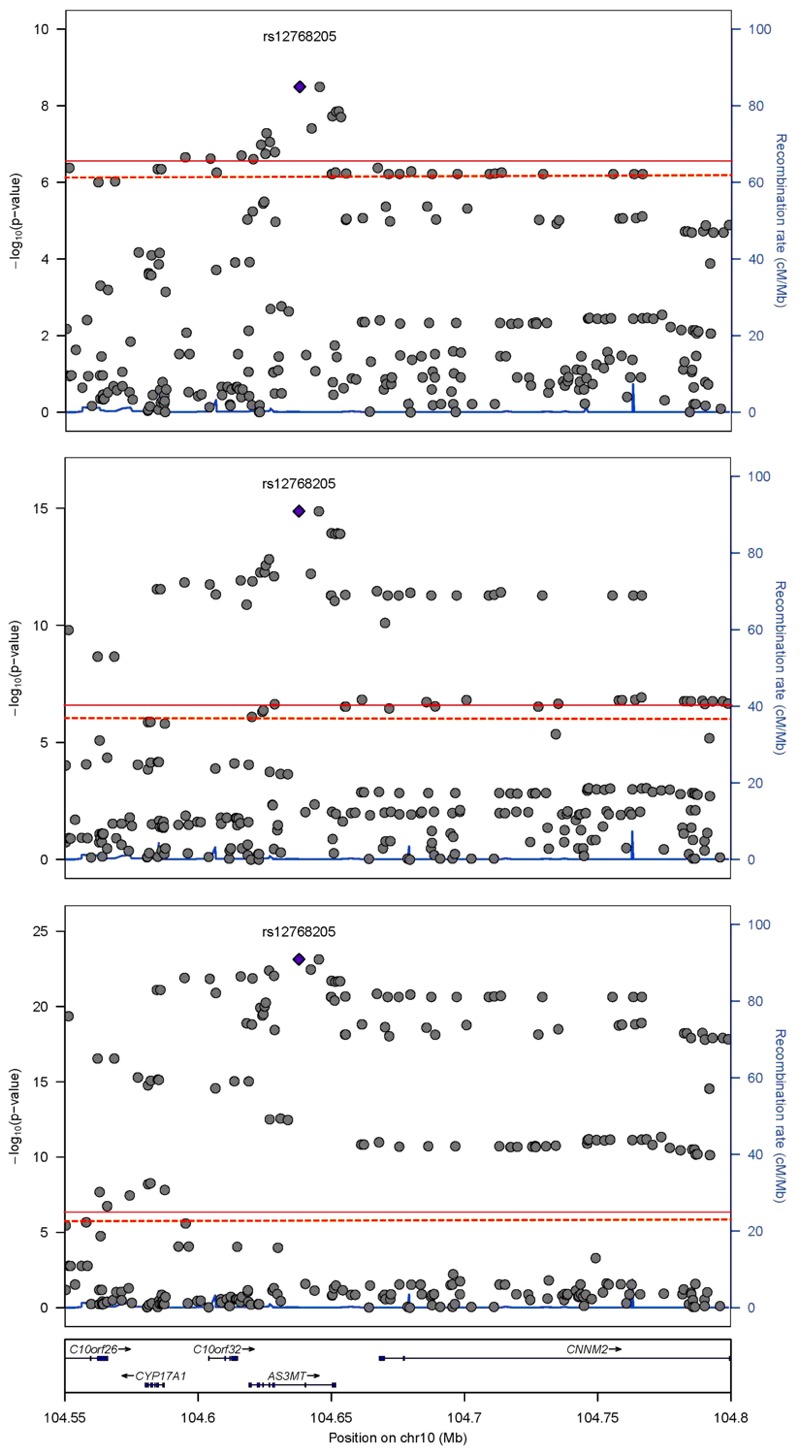
Regional association plot at 10q24 of percent arsenic species. Index single nucleotide polymorphism (SNP) rs12768205 nearby associations according to human genome build 18 for percent inorganic arsenic (iAs%) in top panel, percent monomethylarsonate (MMA%) in middle panel, and percent dimethylarsinate (DMA%) in bottom panel. The solid red line is the MetaboChip-wide significance threshold at –log(4.13 × 10^–7^) or 6.38. The dashed orange line is the suggestive MetaboChip-wide linkage disequilibrium (LD) threshold at –log(7.77 × 10^–7^). Chr, chromosome.

**Figure 2:**
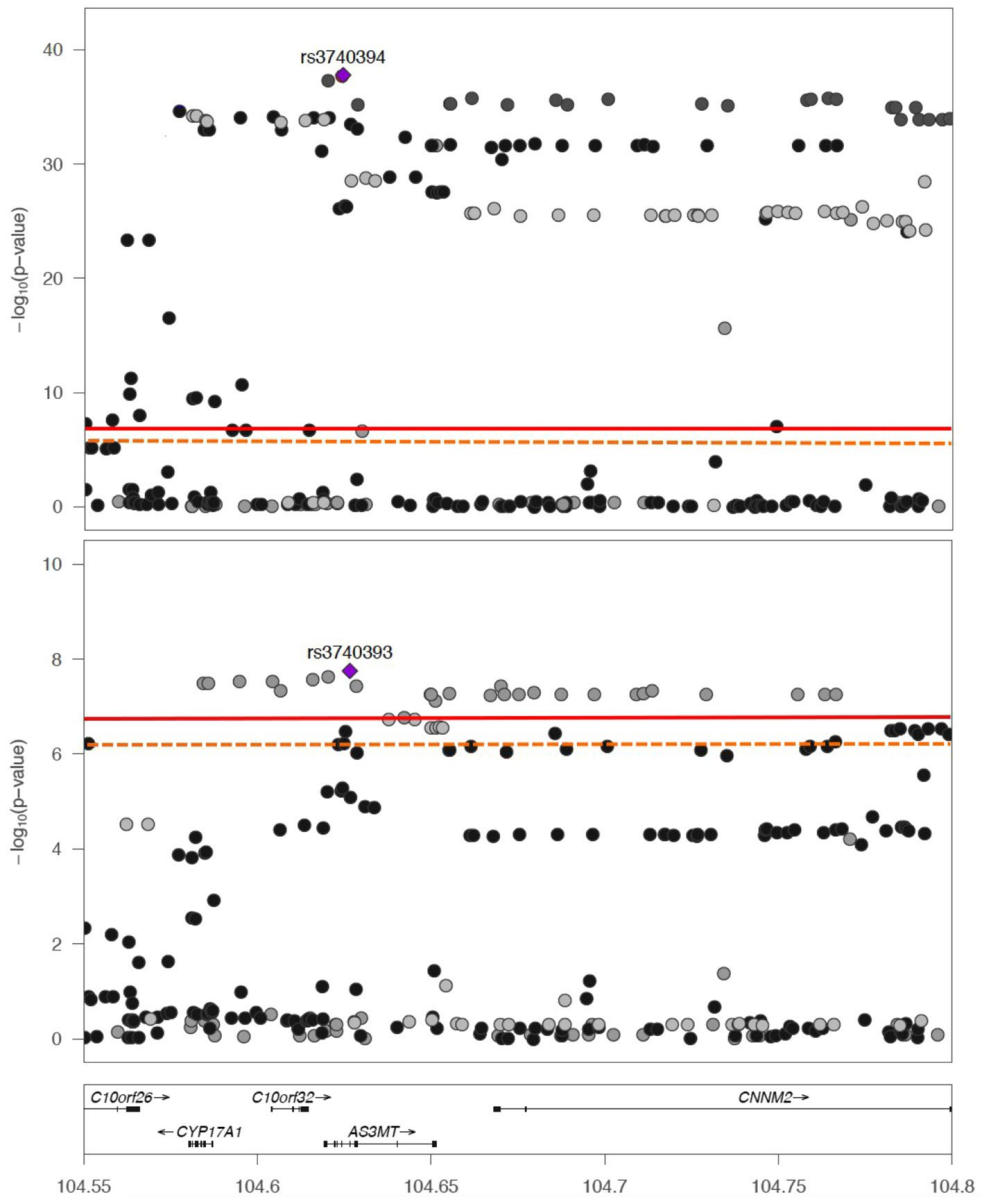
Regional association plot at 10q24 of principal components of arsenic species. Index single nucleotide polymorphisms (SNPs) for principal components (PCs) (top panel PC1 rs3740394, bottom panel PC2 rs3740393) and nearby associations according to human genome build 18. The solid red line is the MetaboChip-wide significance threshold at –log(4.13 × 10^–7^) or 6.38. The dashed orange line is the suggestive MetaboChip-wide linkage disequilibrium (LD) threshold at –log(7.77 × 10^–7^).

The 10q24 region was statistically significantly associated with all logit-transformed percent arsenic species using the MetaboChip SNPs (Figure 1). The index SNP rs12768205 (G > A) in *AS3MT* was consistently associated with percent arsenic species (positively with iAs% and negatively with MMA% and DMA%) and principal components (negatively with PC1 and positively with PC2) (Table 3; see also Tables S3–S7). For PC1, SNPs in 10q24 passed the MetaboChip-wide alpha threshold, and the index SNP rs3740394 (A > G) in *AS3MT* was also associated with DMA% (Table 3). An intronic SNP, rs3740393 (C > G), in *AS3MT* was the top SNP for PC2 and was also significantly associated with MMA%, DMA%, and PC1 (Table 3). Other SNPs within the LD block with pairwise correlation *r* ^2^ > 0.80 of the index SNP (104.62–104.65 mb) were statistically significantly associated with PC1 and PC2 (Figure 2; see also Tables S6 and S7). Quantile-quantile plots (see Figure S1), Manhattan plots (see Figures S3–S7) and top SNP associations (see Tables S2–S6) for MetaboChip SNPs are presented in the supplemental material. Association analyses in the 10q24 region conditioned on the index SNP did not yield any statistically significant independent associations that were not in LD (see Figures S8 and S9).

**Table 3:** Index MetaboChip SNPs of percent arsenic species and principal components of arsenic species.

SNP	Chr	Position^*a*^	Allele	MAF	Gene	Location	Trait	*p*-Value				
								iAs%	MMA%	DMA%	PC1	PC2
rs3740393	10	104626645	C/G	0.17	*AS3MT*	intron	PC2	8.63 × 10^–7^	1.07 × 10^–13^^*b*^	2.71 × 10^–23^^*b*^	2.58 × 10^–34^^*b*^	1.56 × 10^–8^^*b*^
rs3740394	10	104624464	A/G	0.18	*AS3MT*	intron	PC1	1.27 × 10^–4^	4.66 × 10^–7^^*c*^	3.87 × 10^–20^^*b*^	2.19 × 10^–38^^*b*^	5.57 × 10^–6^
rs12768205	10	104637839	G/A	0.27	*AS3MT*	intron	iAs%, MMA%, DMA%	8.27 × 10^–8^^*b*^	1.20 × 10^–15^^*b*^	5.90 × 10^–24^^*b*^	1.15 × 10^–29^^*b*^	1.78 × 10^–7^^*b*^
Notes: Chr, chromosome; DMA%, percent dimethylarsinate; iAs%, percent inorganic arsenic; MMA%, percent monomethylarsonate; MAF, minor allele frequency; PC, principal component; SNP, single nucleotide polymorphism. ^***a***^Base position according to human genome build 18. ^***b***^Significant SNP associations (*p* < 4.13 × 10^–7^). ^***c***^Suggestive SNP associations (*p* < 7.77 × 10^–7^).												

Similar to the MetaboChip SNP associations, the only region that passed the multiple testing correction among the arsenic candidate SNPs was 10q24 (Table 4; see also Tables S8–S12). A total of nine SNPs were associated with at least one trait; five SNPs were intronic, and four SNPs had known functional changes. There were two coding SNPs (rs11191439, T > C in *AS3MT*; rs4925, G > T in *GSTO1*) that alter the amino acid sequence and two SNPs (rs7911488, A > G in *USMG5*; rs2297235, A > G in *GSTO2*) that were located in the 3´ untranslated region in the mRNA after a stop codon. The quantile-quantile plots (see Figure S2) and top SNP associations (see Tables S8–S12) for the arsenic candidate SNPs are presented in the supplemental material. Secondary analyses including the unadjusted models, minimally adjusted models, models stratified by sex, and the conditional models for the arsenic traits did not yield any additional significant SNP associations.

**Table 4:** Top Candidate SNPs of percent arsenic species and principal components of arsenic species.

SNP	Chr	Position^*a*^	Allele	MAF	Gene	Location	*p*-Value				
							iAs%	MMA%	DMA%	PC1	PC2
rs11191439	10	104638723	T/C	0.18	*AS3MT*	coding	3.23 × 10^–4^	2.60 × 10^–7^^*b*^	2.89 × 10^–19^^*b*^	1.12 × 10^–36^^*b*^	7.54 × 10^–7^^*b*^
rs3740394	10	104634474	A/G	0.19	*AS3MT*	intron	2.53 × 10^–6^^*b*^	7.55 × 10^–7^^*b*^	8.30 × 10^–20^^*b*^	2.83 × 10^–38^^*b*^	6.09 × 10^–8^^*b*^
rs3740390	10	104638480	C/T	0.20	*AS3MT*	intron	1.76 × 10^–8^^*b*^	9.24 × 10^–13^^*b*^	8.63 × 10^–23^^*b*^	6.50 × 10^–34^^*b*^	0.99
rs11191453	10	104659852	T/C	0.20	*AS3MT*	intron	6.48 × 10^–7^^*b*^	5.57 × 10^–12^^*b*^	2.18 × 10^–21^^*b*^	1.80 × 10^–32^^*b*^	0.34
rs4919694	10	104698978	T/C	0.18	*CNNM2*	intron	8.10 × 10^–10^^*b*^	4.74 × 10^–7^^*b*^	1.67 × 10^–18^^*b*^	9.67 × 10^–36^^*b*^	3.95 × 10^–8^^*b*^
rs7911488	10	105154089	A/G	0.25	*USMG5*	UTR	1.04 × 10^–6^^*b*^	6.54 × 10^–8^^*b*^	7.04 × 10^–18^^*b*^	3.57 × 10^–31^^*b*^	6.63 × 10^–6^^*b*^
rs4925	10	106022789	C/A	0.12	*GSTO1*	coding	0.02	0.11	1.55 × 10^–3^	4.60 × 10^–6^^*b*^	9.18 × 10^–7^^*b*^
rs1147611	10	106025258	G/T	0.19	*GSTO1*	intron	0.11	0.35	7.23 × 10^–3^	8.45 × 10^–6^^*b*^	0.27
rs2297235	10	106034491	A/G	0.12	*GSTO2*	UTR	0.08	0.20	4.58 × 10^–3^	1.67 × 10^–5^^*b*^	4.31 × 10^–3^
Abbreviations: Chr, chromosome; DMA%, percent dimethylarsinate; iAs%, percent inorganic arsenic; MMA%, percent monomethylarsonate; MAF, minor allele frequency; PC, principal component; SNP, single nucleotide polymorphism. ^***a***^Base position according to human genome build 18. ^***b***^Significant SNP associations (*p* < 9.33 × 10^–5^).											

**Figure 3:**
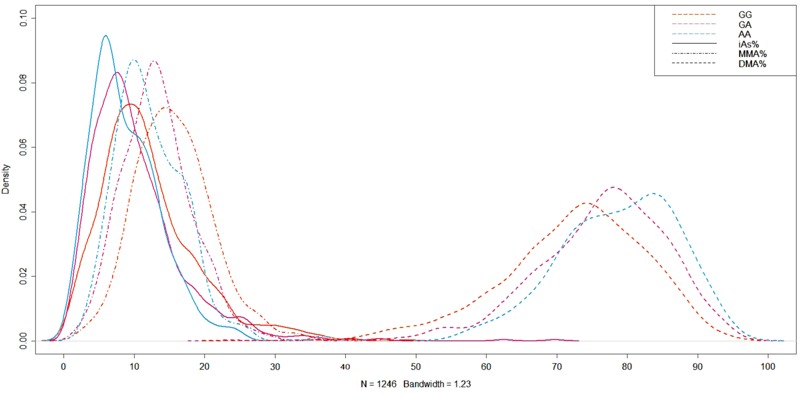
Distribution of percent arsenic species by rs12768205 genotype. Index single nucleotide polymorphism (SNP) rs12768205 for percent arsenic species principal components shows separation of distribution of percent inorganic arsenic (iAs%), percent monomethylarsonate (MMA%) and percent dimethylarsinate (DMA%) by genotype. Of the 2,428 participants, the distribution of genotypes is homozygous major AA (1,245), heterozygous AG (982), and homozygous minor GG (201).

The index SNP rs12768205 explained 1–15% of the heritability of the trait: iAs% (Arizona 5.8%, Oklahoma < 0.1%, North and South Dakota 1.1%), MMA% (Arizona 10.1%, Oklahoma 0.5%, North and South Dakota 2.2%), DMA% (Arizona 12.1%, Oklahoma 14.8%, North and South Dakota 3.4%). At rs12768205, each copy of the variant allele G had a separation in the distribution of percent arsenic species (Figure 3). The variant genotype GG, compared to genotypes GA and AA, had iAs% and MMA% distributions that were shifted towards higher percentages and a DMA% distribution that was shifted towards lower percentages. There was also a clear additive effect with each copy of the variant allele G for each percent arsenic species. The pattern of percent arsenic species by rs12768205 was similar when stratified by study region. The distribution of percent arsenic species by the index SNP genotypes for PC1 (see Figure S10) and PC2 (see Figure S11) are presented in the supplemental material.

## Discussion

We examined common variants (MAF > 1%) in arsenic metabolism among American Indians as measured by logit-transformed iAs%, MMA%, DMA%, and principal components of logit arsenic species (PC1, PC2). The variants evaluated were available on the MetaboChip with nearly 200,000 markers supplemented with a custom panel of 670 candidate SNPs for arsenic metabolism and toxicity. Locus 10q24 was consistently associated with the arsenic phenotypes, similar to previous genome-wide association studies (GWAS) among unrelated individuals in Bangladesh and Vietnam (Agusa et al. 2009; Pierce et al. 2012; Rodrigues et al. 2012) and with a linkage peak in chromosome 10 among SHFS participants (Gribble et al. 2015). In our study, *AS3MT* SNPs produced significant estimates in the 10q24 locus, and the results suggest that common variants play a substantial role in the variation of percent arsenic species and principal components of arsenic species.

In *AS3MT*, rs12768205 was associated with iAs% (*p* = 8.27 × 10^–8^), MMA% (*p* = 1.20 × 10^–15^), and DMA% (*p* = 5.90 × 10^–24^). The index SNPs for the principal components were also located within *AS3MT. AS3MT* encodes the enzyme arsenic (III) methyltransferase, which is responsible for methylation of iAs to MMA/DMA. Prior studies show association with SNPs in LD with rs12768205 and arsenic species and skin lesions, clinical manifestations specific to high chronic arsenic toxicity (Gao et al. 2015; Pierce et al. 2012; Rodrigues et al. 2012). Recently, a population genetics study indicated a positive selection force for *AS3MT* variants in Argentina exposed to high levels of arsenic in drinking water and characterized by elevated urine DMA% (Schlebusch et al. 2015). It is unknown whether this positive selection is specific to this community or if it is present in other populations chronically exposed to arsenic. Our association analysis results indicated that the same loci and possibly genes including *AS3MT, CNNM2*, and *GSTO1* may be involved in arsenic metabolism. The distribution of percent arsenic species showed differentiation by rs12768205 genotype, suggesting that a functional SNP in LD may be a major causal factor in arsenic metabolism. Interestingly, previous GWAS of blood pressure and schizophrenia have found associations with *AS3MT* variants and the 10q24 locus (Cross-Disorder Group of the Psychiatric Genomics Consortium 2013; Newton-Cheh et al. 2009), indicating a possible mechanism of arsenic metabolism and toxicity. It is also possible that *AS3MT* is involved in other metabolic processes beyond arsenic metabolism that have not yet been identified.

Among the candidate arsenic SNPs, functional and potentially causal variants in *AS3MT* were associated consistently with iAs%, MMA%, and DMA%. In particular, a potential candidate, the missense SNP rs11191439 (methionine→threonine) in *AS3MT,* is located within the LD block for rs12768205, the index SNP for percent arsenic species traits. Among the candidate arsenic SNPs, non-*AS3MT* genes also showed signals in our study. An upstream SNP at *USMG5* was associated with all arsenic traits. *USMG5* is a protein coding gene that is involved in skeletal muscle growth (Meyer et al. 2007; Zhao et al. 2014). The function of *USMG5* is unknown but may involve interactions with ATP synthases (Meyer et al. 2007). Interestingly, phosphorylation of proteins encoded by *USMG5* is known to be upregulated by insulin *in vivo* in human skeletal cells (Meyer et al. 2007). In addition, mice exposed to 100 μg/L arsenite for 5 weeks showed impaired muscle function and mitochondrial myopathy compared with controls (Ambrosio et al. 2014). Confirmation, through fine mapping or sequencing as well as through functional analyses, is needed on the relevance of *USMG5* to arsenic metabolism.

A coding variant (rs4925) at *GSTO1* and an upstream variant (rs2297235) at *GSTO2* were associated with PC1. The SNP rs4925 was also associated with PC2. The glutathione *S*-transferase omega (GSTO) family of genes are involved in transferring thiol functional groups and therefore are of importance in phase II metabolism of many xenobiotics. Because of their hypothesized role in oxidative stress and carcinogenesis, polymorphisms in the GSTO family have been candidates for various cancers and late-onset Alzheimer disease (Piacentini et al. 2012; Lima et al. 2008). In a population study based in Bangladesh, *GSTO* variants have been associated with urinary arsenic species (Rodrigues et al. 2012). Although the GSTO family of proteins is implicated in the arsenic reduction pathway (Hernández et al. 2008), we cannot infer causality from our candidate gene association results owing to the strong LD in the region. We also cannot discount the possibility that in addition to the strong LD, the genotype at one variant/gene can affect the expression at another. In fact, *AS3MT* variants may matter for gene expression in the SHS (Gribble et al. 2014). Additional mechanistic and epidemiological research is needed to confirm the relevance of GSTO and other functional variants in arsenic metabolism and toxicity.

The biotransformation pathway for inorganic arsenic is unresolved; prior biological evidence suggests that arsenic metabolism is mainly determined by reduction followed by oxidative methylation (Cullen and Reimer 1989; Vahter and Concha 2001; Vahter 1999). Our results from principal components analysis support this hypothesis. Because iAs% and MMA% are inversely related to DMA%, PC1 may represent the overall methylation to DMA. Because PC2 had an inverse relationship between iAs% and MMA% independent of DMA%, PC2 may represent the first methylation step from iAs to MMA. In addition, the development of arsenic-related chronic diseases may be related to reactive oxygen species and reactive nitrogen species caused by arsenic toxicity (Jomova et al. 2011; Jomova and Valko 2011; Shi et al. 2004; Valko et al. 2007). Metabolizing inorganic arsenic leads to damaging effects to most organs (Shi et al. 2004). An imbalance of reactive oxygen or nitrogen species exceeding the body’s physiological antioxidant defenses can lead to widespread tissue injury, organ dysfunction, and clinical disease via oxidative stress (Jomova et al. 2011; Valko et al. 2007). The genes associated with arsenic species and principal components of arsenic species in our analysis support this oxidative stress hypothesis (Jomova et al. 2011). In particular, previous studies showed that AS3MT and non-AS3MT proteins such as USMG and GSTO can reduce pentavalent arsenic and facilitate transfer of arsenic intermediates as well as antioxidant depletion within and between cells (Hughes 2002; Lefort et al. 2009; Tanaka-Kagawa et al. 2003; Vahter and Concha 2001). These proteins may also lead to oxidative stress via mitochondrial dysregulation from a buildup of free radicals (Lefort et al. 2009; Tanaka-Kagawa et al. 2003), and the ubiquity of mitochondrial function in all tissues may affect most organ systems and chronic diseases (Jomova et al. 2011; Valko et al. 2007).

This is the first large-scale study to assess markers of arsenic metabolism and common variants in a representative U.S. American Indian population sample; it is also one of only a few studies to evaluate genetic determinants of arsenic metabolism in a population exposed to low to moderate levels of arsenic. The Strong Heart Family Study presents a rich family-based cohort with low limits of quantification and limited missing data for arsenic exposure, arsenic species, and other covariates. Another strength of this study is the consistency of the findings within our study with those in the existing literature. In our study, the index SNP rs12768205 located in the *AS3MT* gene was associated with iAs%, MMA%, and DMA%, and SNPs in LD with rs12768205 were associated with PC1 and PC2. *AS3MT* has been previously reported to influence arsenic traits, including total arsenic levels (Argos et al. 2011; Pierce et al. 2012). Although individual urine arsenic metabolites are used in most cohort studies to assess arsenic metabolism, the pattern of arsenic metabolites in blood is different from that in urine, and it is unknown if the genetic determinants for urine arsenic metabolism correspond to the genetic determinants of arsenic metabolism as measured in blood (Goullé et al. 2005; Nixon and Moyer 1996; Tellez-Plaza et al. 2013; Vahter 1999; Valentine et al. 1979). In particular, although blood arsenic levels would be a more proximal biomarker of arsenic metabolism, they tend to be present at much lower levels that are difficult to detect by conventional spectrophotometric methods (Kristiansen et al. 1997). Furthermore, the few epidemiologic studies using blood arsenic measurements, conducted in populations exposed to high levels of arsenic in drinking water, have shown more consistent associations than urine arsenic measurements (Hall et al. 2006; Valentine et al. 1979). No studies, however, have evaluated the association of genetic variants with blood arsenic species. Future research is needed to evaluate whether the genetic variants associated with arsenic metabolism measured in urine are similar to those associated with arsenic metabolism measured in blood. Finally, PCA allowed us not only to reduce the number of dimensions but also to account for the interdependence of arsenic species so that PC1 and PC2 represent independent traits.

The analysis of MetaboChip SNPs allowed us to test hypothesis-driven variants because MetaboChip SNPs were chosen given prior evidence of their association with cardiometabolic diseases. In addition, we assessed SNPs that were previously associated with arsenic species and arsenic-related traits, which resulted in an investigation of SNPs with higher biological plausibility than typical GWAS. However, the use of MetaboChip SNPs also limited the ability to investigate novel SNP associations. Another limitation is that the MetaboChip panel was built using results from European American and African American populations (Voight et al. 2012). The SNP coverage and LD patterns may be different when extrapolated to other populations, although the use of MetaboChip has been characterized among populations with Asian and Mexican ancestry (Crawford et al. 2013). In addition, we were not able to test rare variants. Given the large statistical significance and the high LD observed in the 10q24 locus, it is possible that rare functional variants may be causal. We were able to look at some low-frequency variants (MAF 1–5%) among the candidate genes, but genome sequencing data may be more useful in identifying putative causal SNPs. It is also possible that the multiple testing correction was too strict, and therefore, type II error may be present, particularly in regions of strong LD such as locus 10q24, limiting our ability to find variants with possibly weaker effects than the *AS3MT* variants. Although the SHFS has a relatively small sample size, particularly for a genetic association study, the strength of the associations supports the importance of investigating genetic variants of arsenic metabolism within a prospective American Indian cohort. Finally, given the low to moderate levels of arsenic exposure in Arizona, Oklahoma, and the Dakotas, the generalizability of our findings needs to be further assessed in other populations.

## Conclusion

Association signals in *AS3MT* and surrounding genes in 10q24 are consistently associated with percent arsenic species and principal components of arsenic species. Furthermore, functionally annotated variants in 10q24 also show a strong relationship with the arsenic traits. The associated genes such as *AS3MT* and *GSTO1/2* highlight oxidative stress as a possible mechanism in arsenic biotransformation and therefore in arsenic-related diseases. Given the high LD in the 10q24 region in populations throughout the world (Fujihara et al. 2010; Gomez-Rubio et al. 2010), and particularly among the American Indians in our study, further investigation in comparable populations and using low-frequency variants is needed to confirm our findings. Further knowledge of causal variation may highlight biological mechanisms that are related to arsenic metabolism, including methylation, and may contribute to the elucidation of possible mechanisms for arsenic toxicity and the development of chronic diseases including skin lesions, cancer, and cardiovascular disease.

## Supplemental Material

(6.1 MB) PDF
