# Supplementary material for: Association of Cardiometabolic Genes with Arsenic Metabolism Biomarkers in American Indian Communities: The Strong Heart Family Study (SHFS)
Source: Environ Health Perspect. 2016 Jun 28;125(1):15–22. doi: 10.1289/EHP251 (PMC5226702; doi:10.1289/EHP251)
Supplement: (6.1 MB) PDF [file EHP251.s001.acco.pdf]

Environ Health Perspect
DOI: 10.1289/EHP251

Note to readers with disabilities: *EHP* strives to ensure that all journal content is accessible to all readers. However, some figures and Supplemental Material published in *EHP* articles may not conform to [508 standards](#) due to the complexity of the information being presented. If you need assistance accessing journal content, please contact ehp508@niehs.nih.gov. Our staff will work with you to assess and meet your accessibility needs within 3 working days.

Supplemental Material

Association of Cardiometabolic Genes with Arsenic Metabolism Biomarkers in American Indian Communities: The Strong Heart Family Study (SHFS)

Poojitha Balakrishnan, Dhananjay Vaidya, Nora Franceschini, V. Saroja Voruganti, Matthew O. Gribble, Karin Haack, Sandra Laston, Jason G. Umans, Kevin A. Francesconi, Walter Goessler, Kari E. North, Elisa Lee, Joseph Yracheta, Lyle G. Best, Jean W. MacCluer, Jack Kent, Jr, Shelley A. Cole, and Ana Navas-Acien

Table of Contents

Table S1. Inter-batch variability of urine arsenic measurements (n=46)
Table S2. Fine mapped loci on MetaboChip
Table S3. Top MetaboChip associations for percent inorganic arsenic
Table S4. Top MetaboChip associations for percent monomethylarsonate
Table S5. Top MetaboChip associations for percent dimethylarsinate
Table S6. Top MetaboChip associations for principal component 1 of arsenic species
Table S7. Top MetaboChip associations for principal component 2 of arsenic species
Table S8. Top candidate SNP associations for percent inorganic arsenic
Table S9. Top candidate SNP associations for percent monomethylarsonate
Table S10. Top candidate SNP associations for percent dimethylarsinate
Table S11. Top candidate SNP associations for principal component 1 of arsenic species

Table S12. Top candidate SNP associations for principal component 2 of arsenic species

Figure S1. Quantile-quantile plot for MetaboChip of percent arsenic species and principal components of arsenic species

Figure S2. Quantile-quantile plot for candidate SNPs of percent arsenic species and principal components of arsenic species

Figure S3. Manhattan plot of MetaboChip associations for percent inorganic arsenic

Figure S4. Manhattan plot of MetaboChip associations for percent monomethylarsononate

Figure S5. Manhattan plot of MetaboChip associations for percent dimethylarsinate

Figure S6. Manhattan plot of MetaboChip associations for principal component 1 of arsenic species

Figure S7. Manhattan plot of MetaboChip associations for principal component 2 of arsenic species

Figure S8. Regional association plot at 10q24 of percent arsenic species conditioned on rs12768205

Figure S9. Regional association plot at 10q24 of principal components of arsenic species conditioned on index SNP

Figure S10. Distribution of percent arsenic species by rs3740394 genotype

Figure S11. Distribution of percent arsenic species by rs7098825 genotype

Table S1. Inter-batch variability of urine arsenic measurements (n=46)

**Nies 18**					
	As (Kation)	DMA	MA	As (III+V)	Summe
Mean	**70,6**	**39,0**	**6,2**	**3,3**	**119**
SD	2,7	2,6	0,3	0,5	7
CV %	3,8	6,7	4,1	14,4	5,8
**Nist 2669 I**					
	As (Kation)	DMA	MA	As (III+V)	Summe
Mean	**12,3**	**3,2**	**1,7**	**3,4**	**20,3**
SD	1,6	0,3	0,2	0,3	1,5
CV %	13,1	8,1	11,6	9,3	7,4
**Nist 2669 II**					
	As (Kation)	DMA	MA	As (III+V)	Summe
Mean	**7,5**	**24,6**	**6,9**	**11,1**	**50,2**
SD	0,6	1,4	0,4	0,6	2,3
CV %	7,7	5,5	5,3	5,4	4,5

Table S2. Fine mapped loci on MetaboChip

ID	Symbol	Description	Locus	Chr	Start Position	End Position
4363	ABCC1	ATP-binding cassette, sub-family C (CFTR/MRP), member 1	16p13.1	16	15949577	16143074
1244	ABCC2	ATP-binding cassette, sub-family C (CFTR/MRP), member 2	10q24	10	99782598	99852576
368	ABCC6	ATP-binding cassette, sub-family C (CFTR/MRP), member 6	16p13.1	16	16149565	16223673
328	APEX1	APEX nuclease (multifunctional DNA repair enzyme) 1	14q11.2	14	20455131	20457772
360	AQP3	aquaporin 3 (Gill blood group)	9p13	9	33441154	33447633
364	AQP7	aquaporin 7	9p13	9	33384947	33402645
57412	AS3MT	arsenite methyltransferase	10q24.32	10	102869453	102901899
55799	CACNA2D3	calcium channel, voltage-dependent, alpha 2/delta subunit 3	3p21.1	3	54122593	55074557
11132	CAPN10	calpain 10	2q37.3	2	240586716	240599109
847	CAT	catalase	11p13	11	34438925	34472060
875	CBS	cystathionine-beta-synthase	21q22.3	21	43053191	43076868
55349	CHDH	choline dehydrogenase	3p21.1	3	53816297	53846393
54805	CNNM2	cyclin and CBS domain divalent metal cation transport mediator 2	10q24.32	10	102918293	103078587
1786	DNMT1	DNA (cytosine-5-)-methyltransferase 1	19p13.2	19	10133345	10195079
1788	DNMT3A	DNA (cytosine-5-)-methyltransferase 3 alpha	2p23	2	25232961	25342590
1789	DNMT3B	DNA (cytosine-5-)-methyltransferase 3 beta	20q11.2	20	32762385	32809356
79813	EHMT1	euchromatic histone-lysine N-methyltransferase 1	9q34.3	9	137618992	137836127

ID	Symbol	Description	Locus	Chr	Start Position	End Position
2068	ERCC2	excision repair cross-complementation group 2	19q13.3	19	45351391	45370646
79072	FASTKD3	FAST kinase domains 3	5p15.31	5	7859159	7869037
161835	FSIP1	fibrous sheath interacting protein 1	15q14	15	39599529	39782838
2745	GLRX	glutaredoxin (thioltransferase)	5q14	5	95813849	95822873
2944	GSTM1	glutathione S-transferase mu 1	1p13.3	1	109687796	109693745
9446	GSTO1	glutathione S-transferase omega 1	10q25.1	10	104254194	104267464
119391	GSTO2	glutathione S-transferase omega 2	10q25.1	10	104268416	104304948
2950	GSTP1	glutathione S-transferase pi 1	11q13	11	67583595	67586653
2954	GSTZ1	glutathione S-transferase zeta 1	14q24.3	14	77320887	77331597
10021	HCN4	hyperpolarization activated cyclic nucleotide gated potassium channel 4	15q24.1	15	73319859	73369264
3269	HRH1	histamine receptor H1	3p25	3	11137093	11263253
4049	LTA	lymphotoxin alpha	6p21.3	6	31560550	31574324
4524	MTHFR	methylenetetrahydrofolate reductase (NAD(P)H)	1p36.3	1	11785730	11806103
4548	MTR	5-methyltetrahydrofolate-homocysteine methyltransferase	1q43	1	236794304	236903981
4552	MTRR	5-methyltetrahydrofolate-homocysteine methyltransferase reductase	5p15.31	5	7851186	7901124
4627	MYH9	myosin, heavy chain 9, non-muscle	22q13.1	22	36281277	36388067
29104	N6AMT1	N-6 adenine-specific DNA methyltransferase 1 (putative)	21q21.3	21	28872191	28885373
55655	NLRP2	NLR family, pyrin domain containing 2	19q13.42	19	54965284	55001142
4846	NOS3	nitric oxide synthase 3 (endothelial cell)	7q36	7	150991056	151014599

ID	Symbol	Description	Locus	Chr	Start Position	End Position
4968	OGG1	8-oxoguanine DNA glycosylase	3p26.2	3	9749944	9788246
55644	OSGEP	O-sialoglycoprotein endopeptidase	14q11.2	14	20447048	20455108
84108	PCGF6	polycomb group ring finger 6	10q24.33	10	103302796	103351134
4860	PNP	purine nucleoside phosphorylase	14q13.1	14	20469379	20478006
56979	PRDM9	PR domain containing 9	5p14	5	23507615	23528597
7001	PRDX2	peroxiredoxin 2	19p13.2	19	12796820	12801910
23378	RRP8	ribosomal RNA processing 8, methyltransferase, homolog (yeast)	11p15.4	11	6514830	6603678
57466	SCAF4	SR-related CTD-associated factor 4	21q22.1	21	31671000	31732118
29986	SLC39A2	solute carrier family 39 (zinc transporter), member 2	14q11.2	14	20999255	21001875
10599	SLCO1B1	solute carrier organic anion transporter family, member 1B1	12p	12	21131194	21239796
6647	SOD1	superoxide dismutase 1, soluble	21q22.11	21	31659622	31668931
6648	SOD2	superoxide dismutase 2, mitochondrial	6q25.3	6	159679116	159693321
6649	SOD3	superoxide dismutase 3, extracellular	4p15.2	4	24795463	24800845
7124	TNF	tumor necrosis factor	6p21.3	6	31575567	31578336
7157	TP53	tumor protein p53	17p13.1	17	7668402	7687550
55135	WRAP53	WD repeat containing, antisense to TP53	17p13.1	17	7686071	7703502
7507	XPA	xeroderma pigmentosum, complementation group A	9q22.3	9	97654398	97697409
7515	XRCC1	X-ray repair complementing defective repair in Chinese hamster cells 1	19q13.2	19	43543312	43575578
7517	XRCC3	X-ray repair complementing defective repair in Chinese hamster cells 3	14q32.3	14	103697608	103715486

Start and end base position according to human genome build 19.

Table S3. Top MetaboChip associations for percent inorganic arsenic

SNP	Chr	Position	Alleles	MAF	Gene	Location	Meta P-value	Arizona		Oklahoma		North/South Dakotas	
								Beta	P-value	Beta	P-value	Beta	P-value
rs12768205	10	104637839	G/A	0.27	AS3MT	intron	8.27e-8	-0.26	1.59e-10	0.00	0.99	-0.14	1.68e-3
rs10883796	10	104645305	G/A	0.27	AS3MT	intron	8.27e-8	-0.26	1.59e-10	0.00	0.99	-0.14	1.68e-3
rs3740390	10	104628470	G/A	0.19	AS3MT	intron	3.11e-7	-0.27	4.08e-10	0.00	0.99	-0.16	4.11e-3
rs113282265	10	104647238	A/G	0.19	AS3MT	intron	3.11e-7	-0.27	4.08e-10	0.00	0.99	-0.16	4.11e-3
rs11191447	10	104642313	G/A	0.19	AS3MT	intron	3.11e-7	-0.27	4.08e-10	0.00	0.99	-0.16	4.11e-3
rs10786722	10	104650058	G/A	0.27	AS3MT	intron	3.21e-7	-0.25	3.02e-9	0.00	0.99	-0.14	1.68e-3
rs7897654	10	104652448	A/G	0.27	AS3MT, CNNM2	intergenic	3.21e-7	-0.25	3.02e-9	0.00	0.99	-0.14	1.68e-3
rs10883799	10	104653300	G/A	0.27	AS3MT, CNNM2	intergenic	3.21e-7	-0.25	3.02e-9	0.00	0.99	-0.14	1.68e-3
rs1046778	10	104651474	A/G	0.27	AS3MT	UTR	3.21e-7	-0.25	3.02e-9	0.00	0.99	-0.14	1.68e-3
rs10786736	10	104839106	C/G	0.20	NT5C2	UTR	3.31e-7	-0.26	7.25e-9	0.00	0.99	-0.17	1.11e-3

Abbreviations: Chr, chromosome. MAF, minor allele frequency. SNP, single nucleotide polymorphism.

All 18 SNP associations are below MetaboChip-wide significance threshold of 3.57e-7 for meta-analysis. Base position according to human genome build 18.

Table S4. Top MetaboChip associations for percent monomethylarsonate

SNP	Chr	Position	Alleles	MAF	Gene	Location	Meta P-value	Arizona		Oklahoma		North/South Dakotas	
								Beta	P-value	Beta	P-value	Beta	P-value
rs12768205	10	104637839	G/A	0.27	AS3MT	intron	1.20e-15	-0.26	2.92e-15	-0.08	0.05	-0.15	1.90e-5
rs10883796	10	104645305	G/A	0.27	AS3MT	intron	1.20e-15	-0.26	2.92e-15	-0.08	0.05	-0.15	1.90e-5
rs10786722	10	104650058	G/A	0.27	AS3MT	intron	1.12e-14	-0.24	1.65e-13	-0.08	0.05	-0.15	1.90e-5
rs7897654	10	104652448	A/G	0.27	AS3MT, CNNM2	intergenic	1.12e-14	-0.24	1.65e-13	-0.08	0.05	-0.15	1.90e-5
rs10883799	10	104653300	G/A	0.27	AS3MT, CNNM2	intergenic	1.12e-14	-0.24	1.65e-13	-0.08	0.05	-0.15	1.90e-5
rs1046778	10	104651474	A/G	0.27	AS3MT	UTR	1.12e-14	-0.24	1.65e-13	-0.08	0.05	-0.15	1.90e-5
rs3740393	10	104626645	G/C	0.21	AS3MT	intron	1.07e-13	-0.27	2.25e-15	-0.08	0.09	-0.14	3.24e-4
rs12765002	10	104625338	G/A	0.25	AS3MT	intron	2.31e-13	-0.26	2.97e-14	-0.07	0.09	-0.13	2.42e-4
rs11191447	10	104642313	G/A	0.19	AS3MT	intron	4.56e-13	-0.27	1.62e-14	-0.08	0.10	-0.15	4.29e-4
rs12764049	10	104624946	A/G	0.25	AS3MT	intron	4.91e-13	-0.26	2.97e-14	-0.07	0.12	-0.13	2.42e-4
rs7920697	10	104623327	A/G	0.26	AS3MT	intron	5.05e-13	-0.26	2.97e-14	-0.07	0.13	-0.13	2.37e-4
rs3740390	10	104628470	G/A	0.19	AS3MT	intron	5.52e-13	-0.27	1.62e-14	-0.08	0.11	-0.15	4.29e-4
rs113282265	10	104647238	A/G	0.19	AS3MT	intron	5.52e-13	-0.27	1.62e-14	-0.08	0.11	-0.15	4.29e-4
rs17878846	10	104620402	T/A	0.19	AS3MT	intron	9.63e-13	-0.27	1.49e-14	-0.07	0.14	-0.15	4.50e-4
rs11191416	10	104594906	A/C	0.19	CYP17A1, C10orf32	intergenic	1.08e-12	-0.27	2.43e-14	-0.07	0.13	-0.15	4.50e-4

SNP	Chr	Position	Alleles	MAF	Gene	Location	Meta P-value	Arizona		Oklahoma		North/South Dakotas	
								Beta	P-value	Beta	P-value	Beta	P-value
rs11191425	10	104615960	G/A	0.19	C10orf32, AS3MT	intergenic	1.08e-12	-0.27	2.43e-14	-0.07	0.13	-0.15	4.50e-4
rs188299216	10	104944768	G/A	0.19	NT5C2, LOC401648	intergenic	1.18e-12	-0.26	5.12e-13	-0.08	0.08	-0.15	3.12e-4
rs3824754	10	104604340	G/A	0.19	C10orf32	intron	1.23e-12	-0.27	2.43e-14	-0.07	0.14	-0.15	4.50e-4
rs3824755	10	104585839	C/G	0.19	CYP17A1	intron	1.97e-12	-0.26	4.39e-14	-0.07	0.13	-0.14	5.83e-4
rs1004467	10	104584497	A/G	0.19	CYP17A1	intron	1.97e-12	-0.26	4.39e-14	-0.07	0.13	-0.14	5.83e-4
rs12221064	10	104667116	G/A	0.19	AS3MT, CNNM2	intergenic	2.45e-12	-0.26	6.68e-13	-0.08	0.09	-0.15	4.29e-4
rs11191479	10	104713610	A/G	0.19	CNNM2	intron	2.69e-12	-0.26	6.68e-13	-0.08	0.11	-0.15	3.23e-4
rs11191555	10	104847513	A/C	0.19	NT5C2	intron	3.12e-12	-0.26	6.68e-13	-0.07	0.11	-0.15	3.41e-4
rs79668541	10	104783894	G/A	0.19	CNNM2	intron	3.25e-12	-0.26	4.50e-13	-0.07	0.11	-0.15	4.29e-4
rs4409766	10	104606653	A/G	0.19	C10orf32	intron	3.38e-12	-0.26	4.39e-14	-0.06	0.17	-0.14	5.83e-4
rs78821730	10	104674534	G/A	0.19	CNNM2	intron	3.56e-12	-0.26	6.68e-13	-0.08	0.11	-0.15	4.29e-4
rs11191453	10	104649842	A/G	0.19	AS3MT	intron	3.72e-12	-0.26	6.68e-13	-0.08	0.11	-0.15	4.29e-4
rs12221193	10	104655257	A/C	0.19	AS3MT, CNNM2	intergenic	3.72e-12	-0.26	6.68e-13	-0.08	0.11	-0.15	4.29e-4
rs77180047	10	104656747	G/A	0.19	AS3MT, CNNM2	intergenic	3.72e-12	-0.26	6.68e-13	-0.08	0.11	-0.15	4.29e-4
rs17115213	10	104671133	A/G	0.19	CNNM2	intron	3.72e-12	-0.26	6.68e-13	-0.08	0.11	-0.15	4.29e-4

SNP	Chr	Position	Alleles	MAF	Gene	Location	Meta P-value	Arizona		Oklahoma		North/South Dakotas	
								Beta	P-value	Beta	P-value	Beta	P-value
rs10509759	10	104679655	T/A	0.19	CNNM2	intron	3.72e-12	-0.26	6.68e-13	-0.08	0.11	-0.15	4.29e-4
rs5011520	10	104687506	G/A	0.19	CNNM2	intron	3.72e-12	-0.26	6.68e-13	-0.08	0.11	-0.15	4.29e-4
rs10883808	10	104711116	T/A	0.19	CNNM2	intron	3.72e-12	-0.26	6.68e-13	-0.08	0.11	-0.15	4.29e-4
rs10883815	10	104729169	A/G	0.19	CNNM2	intron	3.72e-12	-0.26	6.68e-13	-0.08	0.11	-0.15	4.29e-4
rs11191502	10	104755484	A/C	0.19	CNNM2	intron	3.72e-12	-0.26	6.68e-13	-0.08	0.11	-0.15	4.29e-4
rs11191514	10	104763354	G/A	0.19	CNNM2	intron	3.72e-12	-0.26	6.68e-13	-0.08	0.11	-0.15	4.29e-4
rs77787671	10	104766195	G/A	0.19	CNNM2	intron	3.72e-12	-0.26	6.68e-13	-0.08	0.11	-0.15	4.29e-4
rs75970938	10	104783638	A/G	0.19	CNNM2	intron	3.72e-12	-0.26	6.68e-13	-0.08	0.11	-0.15	4.29e-4
rs11191535	10	104805866	G/A	0.19	CNNM2	intron	3.72e-12	-0.26	6.68e-13	-0.08	0.11	-0.15	4.29e-4
rs10458729	10	104831469	G/A	0.19	CNNM2, NT5C2	intergenic	3.72e-12	-0.26	6.68e-13	-0.08	0.11	-0.15	4.29e-4
rs11191454	10	104649994	A/G	0.19	AS3MT	intron	3.72e-12	-0.26	6.68e-13	-0.08	0.11	-0.15	4.29e-4
rs11191472	10	104697006	T/A	0.19	CNNM2	intron	3.72e-12	-0.26	6.68e-13	-0.08	0.11	-0.15	4.29e-4
rs11191515	10	104766517	G/A	0.19	CNNM2	intron	3.72e-12	-0.26	6.68e-13	-0.08	0.11	-0.15	4.29e-4
rs11191548	10	104836168	A/G	0.19	CNNM2, NT5C2	intergenic	3.72e-12	-0.26	6.68e-13	-0.08	0.11	-0.15	4.29e-4
rs12411886	10	104675289	C/A	0.19	CNNM2	intron	3.72e-12	-0.26	6.68e-13	-0.08	0.11	-0.15	4.29e-4
rs12413409	10	104709086	G/A	0.19	CNNM2	intron	3.72e-12	-0.26	6.68e-13	-0.08	0.11	-0.15	4.29e-4
rs3781285	10	104815655	C/G	0.19	CNNM2	intron	3.72e-12	-0.26	6.68e-13	-0.08	0.11	-0.15	4.29e-4

SNP	Chr	Position	Alleles	MAF	Gene	Location	Meta P-value	Arizona		Oklahoma		North/South Dakotas	
								Beta	P-value	Beta	P-value	Beta	P-value
rs12219901	10	104830957	A/G	0.19	CNNM2, NT5C2	intergenic	3.90e-12	-0.26	6.68e-13	-0.07	0.11	-0.15	4.29e-4
rs732998	10	104887891	A/G	0.19	NT5C2	intron	3.99e-12	-0.26	6.52e-13	-0.07	0.11	-0.15	4.29e-4
rs79237883	10	104930936	A/G	0.19	LOC729081, NT5C2	intergenic	4.05e-12	-0.26	6.68e-13	-0.07	0.11	-0.15	4.29e-4
rs77420391	10	104935813	G/A	0.19	NT5C2	intron	4.05e-12	-0.26	6.68e-13	-0.07	0.11	-0.15	4.29e-4
rs11191558	10	104854668	G/A	0.19	NT5C2	intron	4.05e-12	-0.26	6.68e-13	-0.07	0.11	-0.15	4.29e-4
rs11191560	10	104859028	A/G	0.19	NT5C2	intron	4.05e-12	-0.26	6.68e-13	-0.07	0.11	-0.15	4.29e-4
rs11191580	10	104896201	A/G	0.19	NT5C2	intron	4.05e-12	-0.26	6.68e-13	-0.07	0.11	-0.15	4.29e-4
rs11191582	10	104903643	G/A	0.19	NT5C2	intron	4.05e-12	-0.26	6.68e-13	-0.07	0.11	-0.15	4.29e-4
rs12220375	10	104891481	A/G	0.19	NT5C2	intron	4.05e-12	-0.26	6.68e-13	-0.07	0.11	-0.15	4.29e-4
rs12413046	10	104861194	A/G	0.19	NT5C2	intron	4.05e-12	-0.26	6.68e-13	-0.07	0.11	-0.15	4.29e-4
rs9633712	10	104863751	G/C	0.19	NT5C2	intron	4.05e-12	-0.26	6.68e-13	-0.07	0.11	-0.15	4.29e-4
rs12412038	10	104846152	G/A	0.19	NT5C2	intron	4.06e-12	-0.26	6.68e-13	-0.07	0.11	-0.15	4.30e-4
rs12217501	10	104841879	A/G	0.19	NT5C2	intron	4.25e-12	-0.26	6.06e-13	-0.07	0.12	-0.15	4.29e-4
rs10883832	10	104861269	A/C	0.19	NT5C2	intron	4.34e-12	-0.26	6.68e-13	-0.07	0.12	-0.15	4.29e-4
rs12220743	10	104841902	G/A	0.19	NT5C2	intron	4.38e-12	-0.26	6.68e-13	-0.07	0.12	-0.15	4.29e-4
rs943037	10	104825909	G/A	0.19	CNNM2	coding	4.38e-12	-0.26	6.68e-13	-0.07	0.12	-0.15	4.29e-4
rs79780963	10	104942489	G/A	0.19	NT5C2	intron	5.06e-12	-0.25	6.72e-13	-0.07	0.13	-0.15	4.36e-4

SNP	Chr	Position	Alleles	MAF	Gene	Location	Meta P-value	Arizona		Oklahoma		North/South Dakotas	
								Beta	P-value	Beta	P-value	Beta	P-value
rs1060240	10	104873327	A/G	0.19	NT5C2	intron	5.37e-12	-0.26	4.53e-13	-0.07	0.15	-0.15	4.04e-4
rs184992072	10	104846756	A/G	0.19	NT5C2	intron	5.81e-12	-0.26	6.92e-13	-0.07	0.13	-0.15	4.67e-4
rs17884001	10	104651235	G/A	0.19	AS3MT	UTR	6.37e-12	-0.26	6.68e-13	-0.07	0.14	-0.15	4.29e-4
rs10430665	10	104838420	G/A	0.19	NT5C2	UTR	8.30e-12	-0.25	8.97e-13	-0.07	0.15	-0.15	4.24e-4
rs7098825	10	104618224	A/G	0.17	C10orf32, AS3MT	intergenic	9.01e-12	-0.30	6.80e-13	-0.05	0.33	-0.18	8.90e-5
rs11191595	10	104933038	A/C	0.19	NT5C2	intron	9.75e-12	-0.25	9.71e-13	-0.07	0.16	-0.15	4.36e-4
rs10786736	10	104839106	C/G	0.20	NT5C2	UTR	1.19e-11	-0.25	1.13e-12	-0.06	0.18	-0.14	3.73e-4
rs2297787	10	104670127	T/A	0.20	CNNM2	intron	5.61e-11	-0.25	1.69e-12	-0.06	0.22	-0.14	8.45e-4
rs2482507	10	104551346	G/A	0.21	C10orf26	intron	1.22e-10	-0.22	6.49e-11	-0.05	0.25	-0.15	2.30e-4
rs1926032	10	104819459	G/A	0.18	CNNM2	intron	2.92e-10	-0.24	1.22e-11	-0.03	0.50	-0.16	2.11e-4
rs284841	10	104540069	A/G	0.20	C10orf26	intron	5.51e-10	-0.23	4.44e-11	-0.06	0.23	-0.13	1.30e-3
rs284844	10	104544519	G/A	0.21	C10orf26	intron	5.51e-10	-0.23	4.44e-11	-0.06	0.23	-0.13	1.30e-3
rs284861	10	104562266	G/A	0.15	C10orf26	intron	1.61e-9	-0.24	2.46e-8	-0.10	0.07	-0.14	1.20e-3
rs284851	10	104568521	G/C	0.15	C10orf26, CYP17A1	intergenic	1.61e-9	-0.24	2.46e-8	-0.10	0.07	-0.14	1.20e-3
rs4917384	10	104985778	G/A	0.26	LOC401648, LOC729020	intergenic	8.26e-9	-0.22	3.04e-10	-0.05	0.23	-0.10	6.08e-3
rs10509763	10	104766381	G/A	0.18	CNNM2	intron	1.32e-7	0.12	1.22e-3	0.10	0.05	0.14	8.00e-5

SNP	Chr	Position	Alleles	MAF	Gene	Location	Meta P-value	Arizona		Oklahoma		North/South Dakotas	
								Beta	P-value	Beta	P-value	Beta	P-value
rs74749600	10	104722986	A/G	0.18	CNNM2	intron	1.40e-7	0.12	1.08e-3	0.10	0.06	0.14	7.60e-5
rs78893207	10	104786128	A/G	0.17	CNNM2	intron	1.47e-7	0.12	1.39e-3	0.10	0.05	0.14	8.00e-5
rs76892505	10	104709280	G/A	0.18	CNNM2	intron	1.50e-7	0.12	1.77e-3	0.11	0.05	0.14	7.60e-5
rs74376228	10	104667652	C/G	0.18	AS3MT, CNNM2	intergenic	1.60e-7	0.12	1.72e-3	0.11	0.05	0.14	7.60e-5
rs80327774	10	104646661	A/G	0.18	AS3MT	intron	1.63e-7	0.12	1.20e-3	0.10	0.06	0.14	8.00e-5
rs12251035	10	104661449	A/C	0.18	AS3MT, CNNM2	intergenic	1.65e-7	0.12	1.22e-3	0.10	0.06	0.14	8.00e-5
rs11191473	10	104700700	G/C	0.18	CNNM2	intron	1.65e-7	0.12	1.22e-3	0.10	0.06	0.14	8.00e-5
rs7100592	10	104759088	A/C	0.18	CNNM2	intron	1.65e-7	0.12	1.22e-3	0.10	0.06	0.14	8.00e-5
rs12245343	10	104764040	A/G	0.18	CNNM2	intron	1.65e-7	0.12	1.22e-3	0.10	0.06	0.14	8.00e-5
rs79331374	10	104872903	G/A	0.17	NT5C2	intron	1.67e-7	0.12	1.39e-3	0.10	0.06	0.14	8.00e-5
rs12258551	10	104757882	G/C	0.18	CNNM2	intron	1.78e-7	0.12	1.22e-3	0.10	0.06	0.14	8.80e-5
rs11191523	10	104782390	A/G	0.18	CNNM2	intron	1.85e-7	0.12	1.39e-3	0.10	0.06	0.14	8.00e-5
rs11191525	10	104783425	C/G	0.18	CNNM2	intron	1.85e-7	0.12	1.39e-3	0.10	0.06	0.14	8.00e-5
rs58317752	10	104789257	A/T	0.18	CNNM2	intron	1.85e-7	0.12	1.39e-3	0.10	0.06	0.14	8.00e-5
rs77827514	10	104811999	G/A	0.18	CNNM2	intron	1.85e-7	0.12	1.39e-3	0.10	0.06	0.14	8.00e-5
rs78214351	10	104812678	A/G	0.18	CNNM2	intron	1.85e-7	0.12	1.39e-3	0.10	0.06	0.14	8.00e-5
rs12257941	10	104823151	G/A	0.18	CNNM2	intron	1.85e-7	0.12	1.39e-3	0.10	0.06	0.14	8.00e-5

SNP	Chr	Position	Alleles	MAF	Gene	Location	Meta P-value	Arizona		Oklahoma		North/South Dakotas	
								Beta	P-value	Beta	P-value	Beta	P-value
rs12252500	10	104784974	G/C	0.17	CNNM2	intron	1.95e-7	0.12	1.39e-3	0.10	0.06	0.14	8.00e-5
rs12257935	10	104793052	C/A	0.17	CNNM2	intron	1.95e-7	0.12	1.39e-3	0.10	0.06	0.14	8.00e-5
rs12266291	10	104796874	G/A	0.17	CNNM2	intron	1.95e-7	0.12	1.39e-3	0.10	0.06	0.14	8.00e-5
rs11191545	10	104819783	G/A	0.17	CNNM2	intron	2.00e-7	0.12	1.39e-3	0.10	0.07	0.14	8.00e-5
rs77505796	10	104637764	T/A	0.18	AS3MT	intron	2.10e-7	0.12	1.77e-3	0.10	0.06	0.14	7.60e-5
rs17787717	10	104685689	A/C	0.18	CNNM2	intron	2.10e-7	0.12	1.77e-3	0.10	0.06	0.14	7.60e-5
rs75219158	10	104737584	G/A	0.18	CNNM2	intron	2.10e-7	0.12	1.77e-3	0.10	0.06	0.14	7.60e-5
rs17727391	10	104826091	C/A	0.18	CNNM2	intron	2.36e-7	0.12	1.39e-3	0.10	0.06	0.14	1.09e-4
rs17727044	10	104811246	T/A	0.17	CNNM2	intron	2.37e-7	0.12	2.03e-3	0.10	0.06	0.14	7.60e-5
rs58700372	10	104790195	A/G	0.17	CNNM2	intron	2.45e-7	0.12	1.39e-3	0.10	0.08	0.14	8.00e-5
rs12241091	10	104798987	G/A	0.17	CNNM2	intron	2.45e-7	0.12	1.39e-3	0.10	0.08	0.14	8.00e-5
rs11191490	10	104735164	T/A	0.18	CNNM2	intron	2.46e-7	0.11	1.95e-3	0.10	0.06	0.14	8.00e-5
rs11191439	10	104628713	A/G	0.18	AS3MT	coding	2.58e-7	0.12	1.15e-3	0.10	0.06	0.14	1.46e-4
rs76255497	10	104641945	A/G	0.18	AS3MT	intron	3.16e-7	0.12	1.22e-3	0.09	0.10	0.14	8.00e-5
rs12253284	10	104655135	C/G	0.18	AS3MT, CNNM2	intergenic	3.16e-7	0.12	1.22e-3	0.09	0.10	0.14	8.00e-5
rs12261040	10	104655387	A/G	0.18	AS3MT, CNNM2	intergenic	3.16e-7	0.12	1.22e-3	0.09	0.10	0.14	8.00e-5
rs10509758	10	104671700	A/G	0.18	CNNM2	intron	3.16e-7	0.12	1.22e-3	0.09	0.10	0.14	8.00e-5

SNP	Chr	Position	Alleles	MAF	Gene	Location	Meta P-value	Arizona		Oklahoma		North/South Dakotas	
								Beta	P-value	Beta	P-value	Beta	P-value
rs4919694	10	104688968	A/G	0.18	CNNM2	intron	3.16e-7	0.12	1.22e-3	0.09	0.10	0.14	8.00e-5
rs12268849	10	104727672	G/A	0.18	CNNM2	intron	3.16e-7	0.12	1.22e-3	0.09	0.10	0.14	8.00e-5

Abbreviations: Chr, chromosome. MAF, minor allele frequency. SNP, single nucleotide polymorphism.

All 116 SNP associations are below MetaboChip-wide significance threshold of 3.57e-7 for meta-analysis. Base position according to human genome build 18.

Table S5. Top MetaboChip associations for percent dimethylarsinate

SNP	Chr	Position	Alleles	MAF	Gene	Location	Meta P-value	Arizona		Oklahoma		North/South Dakotas	
								Beta	P-value	Beta	P-value	Beta	P-value
rs12768205	10	104637839	G/A	0.27	AS3MT	intron	5.90e-24	0.28	1.33e-18	0.11	5.76e-4	0.17	3.89e-8
rs10883796	10	104645305	G/A	0.27	AS3MT	intron	5.90e-24	0.28	1.33e-18	0.11	5.76e-4	0.17	3.89e-8
rs11191447	10	104642313	G/A	0.19	AS3MT	intron	2.54e-23	0.30	2.60e-18	0.13	6.41e-4	0.20	8.59e-8
rs3740393	10	104626645	G/C	0.21	AS3MT	intron	2.71e-23	0.30	1.91e-18	0.13	3.52e-4	0.18	2.47e-7
rs3740390	10	104628470	G/A	0.19	AS3MT	intron	4.36e-23	0.30	2.60e-18	0.12	8.99e-4	0.20	8.59e-8
rs113282265	10	104647238	A/G	0.19	AS3MT	intron	4.36e-23	0.30	2.60e-18	0.12	8.99e-4	0.20	8.59e-8
rs11191416	10	104594906	A/C	0.19	CYP17A1, C10orf32	intergenic	6.40e-23	0.30	4.13e-18	0.12	1.02e-3	0.20	7.83e-8
rs11191425	10	104615960	G/A	0.19	C10orf32, AS3MT	intergenic	6.40e-23	0.30	4.13e-18	0.12	1.02e-3	0.20	7.83e-8
rs3824754	10	104604340	G/A	0.19	C10orf32	intron	6.80e-23	0.30	4.13e-18	0.12	1.06e-3	0.20	7.83e-8
rs17878846	10	104620402	T/A	0.19	AS3MT	intron	6.90e-23	0.30	4.23e-18	0.12	1.06e-3	0.20	7.83e-8
rs188299216	10	104944768	G/A	0.19	NT5C2, LOC401648	intergenic	8.83e-23	0.28	4.19e-16	0.13	3.86e-4	0.20	2.95e-8
rs10786722	10	104650058	G/A	0.27	AS3MT	intron	1.85e-22	0.26	3.24e-16	0.11	5.76e-4	0.17	3.89e-8
rs7897654	10	104652448	A/G	0.27	AS3MT, CNNM2	intergenic	1.85e-22	0.26	3.24e-16	0.11	5.76e-4	0.17	3.89e-8
rs10883799	10	104653300	G/A	0.27	AS3MT,	intergenic	1.85e-22	0.26	3.24e-16	0.11	5.76e-4	0.17	3.89e-8

SNP	Chr	Position	Alleles	MAF	Gene	Location	Meta P-value	Arizona		Oklahoma		North/South Dakotas	
								Beta	P-value	Beta	P-value	Beta	P-value
					CNNM2								
rs1046778	10	104651474	A/G	0.27	AS3MT	UTR	1.85e-22	0.26	3.24e-16	0.11	5.76e-4	0.17	3.89e-8
rs3824755	10	104585839	C/G	0.19	CYP17A1	intron	3.82e-22	0.30	4.53e-18	0.12	1.02e-3	0.18	3.70e-7
rs1004467	10	104584497	A/G	0.19	CYP17A1	intron	3.82e-22	0.30	4.53e-18	0.12	1.02e-3	0.18	3.70e-7
rs77420391	10	104935813	G/A	0.19	NT5C2	intron	5.19e-22	0.28	5.20e-16	0.12	9.06e-4	0.20	3.96e-8
rs11191582	10	104903643	G/A	0.19	NT5C2	intron	5.19e-22	0.28	5.20e-16	0.12	9.06e-4	0.20	3.96e-8
rs4409766	10	104606653	A/G	0.19	C10orf32	intron	6.73e-22	0.30	4.53e-18	0.12	1.45e-3	0.18	3.70e-7
rs12221064	10	104667116	G/A	0.19	AS3MT, CNNM2	intergenic	7.81e-22	0.28	5.20e-16	0.13	6.96e-4	0.20	8.59e-8
rs11191479	10	104713610	A/G	0.19	CNNM2	intron	9.82e-22	0.28	5.20e-16	0.12	8.99e-4	0.20	7.33e-8
rs78821730	10	104674534	G/A	0.19	CNNM2	intron	1.08e-21	0.28	5.20e-16	0.12	8.56e-4	0.20	8.59e-8
rs732998	10	104887891	A/G	0.19	NT5C2	intron	1.15e-21	0.28	5.02e-16	0.12	9.06e-4	0.20	8.59e-8
rs11191453	10	104649842	A/G	0.19	AS3MT	intron	1.16e-21	0.28	5.20e-16	0.12	8.99e-4	0.20	8.59e-8
rs12221193	10	104655257	A/C	0.19	AS3MT, CNNM2	intergenic	1.16e-21	0.28	5.20e-16	0.12	8.99e-4	0.20	8.59e-8
rs77180047	10	104656747	G/A	0.19	AS3MT, CNNM2	intergenic	1.16e-21	0.28	5.20e-16	0.12	8.99e-4	0.20	8.59e-8
rs17115213	10	104671133	A/G	0.19	CNNM2	intron	1.16e-21	0.28	5.20e-16	0.12	8.99e-4	0.20	8.59e-8
rs10509759	10	104679655	T/A	0.19	CNNM2	intron	1.16e-21	0.28	5.20e-16	0.12	8.99e-4	0.20	8.59e-8
rs5011520	10	104687506	G/A	0.19	CNNM2	intron	1.16e-21	0.28	5.20e-16	0.12	8.99e-4	0.20	8.59e-8

SNP	Chr	Position	Alleles	MAF	Gene	Location	Meta P-value	Arizona		Oklahoma		North/South Dakotas	
								Beta	P-value	Beta	P-value	Beta	P-value
rs10883808	10	104711116	T/A	0.19	CNNM2	intron	1.16e-21	0.28	5.20e-16	0.12	8.99e-4	0.20	8.59e-8
rs10883815	10	104729169	A/G	0.19	CNNM2	intron	1.16e-21	0.28	5.20e-16	0.12	8.99e-4	0.20	8.59e-8
rs11191502	10	104755484	A/C	0.19	CNNM2	intron	1.16e-21	0.28	5.20e-16	0.12	8.99e-4	0.20	8.59e-8
rs11191514	10	104763354	G/A	0.19	CNNM2	intron	1.16e-21	0.28	5.20e-16	0.12	8.99e-4	0.20	8.59e-8
rs77787671	10	104766195	G/A	0.19	CNNM2	intron	1.16e-21	0.28	5.20e-16	0.12	8.99e-4	0.20	8.59e-8
rs75970938	10	104783638	A/G	0.19	CNNM2	intron	1.16e-21	0.28	5.20e-16	0.12	8.99e-4	0.20	8.59e-8
rs11191535	10	104805866	G/A	0.19	CNNM2	intron	1.16e-21	0.28	5.20e-16	0.12	8.99e-4	0.20	8.59e-8
rs10458729	10	104831469	G/A	0.19	CNNM2, NT5C2	intergenic	1.16e-21	0.28	5.20e-16	0.12	8.99e-4	0.20	8.59e-8
rs11191454	10	104649994	A/G	0.19	AS3MT	intron	1.16e-21	0.28	5.20e-16	0.12	8.99e-4	0.20	8.59e-8
rs11191472	10	104697006	T/A	0.19	CNNM2	intron	1.16e-21	0.28	5.20e-16	0.12	8.99e-4	0.20	8.59e-8
rs11191515	10	104766517	G/A	0.19	CNNM2	intron	1.16e-21	0.28	5.20e-16	0.12	8.99e-4	0.20	8.59e-8
rs11191548	10	104836168	A/G	0.19	CNNM2, NT5C2	intergenic	1.16e-21	0.28	5.20e-16	0.12	8.99e-4	0.20	8.59e-8
rs12411886	10	104675289	C/A	0.19	CNNM2	intron	1.16e-21	0.28	5.20e-16	0.12	8.99e-4	0.20	8.59e-8
rs12413409	10	104709086	G/A	0.19	CNNM2	intron	1.16e-21	0.28	5.20e-16	0.12	8.99e-4	0.20	8.59e-8
rs3781285	10	104815655	C/G	0.19	CNNM2	intron	1.16e-21	0.28	5.20e-16	0.12	8.99e-4	0.20	8.59e-8
rs11191555	10	104847513	A/C	0.19	NT5C2	intron	1.17e-21	0.28	5.20e-16	0.12	9.06e-4	0.20	8.51e-8
rs10786736	10	104839106	C/G	0.20	NT5C2	UTR	1.17e-21	0.28	7.76e-16	0.11	1.93e-3	0.20	2.09e-8

SNP	Chr	Position	Alleles	MAF	Gene	Location	Meta P-value	Arizona		Oklahoma		North/South Dakotas	
								Beta	P-value	Beta	P-value	Beta	P-value
rs79237883	10	104930936	A/G	0.19	LOC729081, NT5C2	intergenic	1.18e-21	0.28	5.20e-16	0.12	9.06e-4	0.20	8.59e-8
rs11191558	10	104854668	G/A	0.19	NT5C2	intron	1.18e-21	0.28	5.20e-16	0.12	9.06e-4	0.20	8.59e-8
rs11191560	10	104859028	A/G	0.19	NT5C2	intron	1.18e-21	0.28	5.20e-16	0.12	9.06e-4	0.20	8.59e-8
rs11191580	10	104896201	A/G	0.19	NT5C2	intron	1.18e-21	0.28	5.20e-16	0.12	9.06e-4	0.20	8.59e-8
rs12220375	10	104891481	A/G	0.19	NT5C2	intron	1.18e-21	0.28	5.20e-16	0.12	9.06e-4	0.20	8.59e-8
rs12413046	10	104861194	A/G	0.19	NT5C2	intron	1.18e-21	0.28	5.20e-16	0.12	9.06e-4	0.20	8.59e-8
rs9633712	10	104863751	G/C	0.19	NT5C2	intron	1.18e-21	0.28	5.20e-16	0.12	9.06e-4	0.20	8.59e-8
rs12412038	10	104846152	G/A	0.19	NT5C2	intron	1.18e-21	0.28	5.20e-16	0.12	9.06e-4	0.20	8.62e-8
rs79780963	10	104942489	G/A	0.19	NT5C2	intron	1.39e-21	0.28	1.65e-15	0.12	1.05e-3	0.20	4.04e-8
rs12220743	10	104841902	G/A	0.19	NT5C2	intron	1.41e-21	0.28	5.20e-16	0.12	1.02e-3	0.20	8.59e-8
rs943037	10	104825909	G/A	0.19	CNNM2	coding	1.41e-21	0.28	5.20e-16	0.12	1.02e-3	0.20	8.59e-8
rs10883832	10	104861269	A/C	0.19	NT5C2	intron	1.45e-21	0.28	5.20e-16	0.12	1.04e-3	0.20	8.59e-8
rs12217501	10	104841879	A/G	0.19	NT5C2	intron	1.50e-21	0.28	4.89e-16	0.12	1.08e-3	0.20	8.59e-8
rs12219901	10	104830957	A/G	0.19	CNNM2, NT5C2	intergenic	1.71e-21	0.28	5.20e-16	0.12	1.15e-3	0.20	8.59e-8
rs1060240	10	104873327	A/G	0.19	NT5C2	intron	2.02e-21	0.28	3.59e-16	0.12	1.64e-3	0.20	7.28e-8
rs17884001	10	104651235	G/A	0.19	AS3MT	UTR	2.04e-21	0.28	5.20e-16	0.12	1.28e-3	0.20	8.59e-8
rs79668541	10	104783894	G/A	0.19	CNNM2	intron	3.35e-21	0.28	2.01e-15	0.12	1.02e-3	0.20	8.59e-8

SNP	Chr	Position	Alleles	MAF	Gene	Location	Meta P-value	Arizona		Oklahoma		North/South Dakotas	
								Beta	P-value	Beta	P-value	Beta	P-value
rs12765002	10	104625338	G/A	0.25	AS3MT	intron	3.42e-21	0.29	2.74e-18	0.10	2.08e-3	0.16	1.18e-6
rs10430665	10	104838420	G/A	0.19	NT5C2	UTR	4.07e-21	0.28	6.42e-16	0.12	1.84e-3	0.20	8.38e-8
rs184992072	10	104846756	A/G	0.19	NT5C2	intron	5.35e-21	0.28	5.50e-16	0.12	1.22e-3	0.19	2.19e-7
rs12764049	10	104624946	A/G	0.25	AS3MT	intron	7.03e-21	0.29	2.74e-18	0.10	3.20e-3	0.16	1.18e-6
rs7920697	10	104623327	A/G	0.26	AS3MT	intron	8.77e-21	0.29	2.74e-18	0.10	3.73e-3	0.16	1.14e-6
rs11191595	10	104933038	A/C	0.19	NT5C2	intron	1.23e-20	0.27	3.88e-15	0.12	1.76e-3	0.20	8.76e-8
rs2482507	10	104551346	G/A	0.21	C10orf26	intron	2.41e-20	0.26	1.81e-14	0.11	3.22e-3	0.19	2.42e-8
rs3740394	10	104624464	A/G	0.18	AS3MT	intron	3.87e-20	-0.21	7.93e-9	-0.19	1.10e-5	-0.19	7.28e-9
rs10509760	10	104624097	A/G	0.18	AS3MT	intron	4.69e-20	-0.21	7.93e-9	-0.19	1.10e-5	-0.19	8.89e-9
rs7098825	10	104618224	A/G	0.17	C10orf32, AS3MT	intergenic	6.37e-20	0.32	2.74e-15	0.12	6.81e-3	0.22	5.79e-8
rs74376228	10	104667652	C/G	0.18	AS3MT, CNNM2	intergenic	1.19e-19	-0.21	9.56e-9	-0.18	2.90e-5	-0.19	5.96e-9
rs2297787	10	104670127	T/A	0.20	CNNM2	intron	1.21e-19	0.27	2.04e-15	0.11	3.54e-3	0.18	3.79e-7
rs76892505	10	104709280	G/A	0.18	CNNM2	intron	1.32e-19	-0.20	1.50e-8	-0.18	2.30e-5	-0.19	5.96e-9
rs10509763	10	104766381	G/A	0.18	CNNM2	intron	1.47e-19	-0.21	7.47e-9	-0.18	3.20e-5	-0.19	8.15e-9
rs80327774	10	104646661	A/G	0.18	AS3MT	intron	1.73e-19	-0.21	7.38e-9	-0.18	3.70e-5	-0.19	8.15e-9
rs4917986	10	104620171	A/G	0.18	AS3MT	intron	1.76e-19	-0.21	7.93e-9	-0.17	3.90e-5	-0.19	7.28e-9
rs12251035	10	104661449	A/C	0.18	AS3MT, CNNM2	intergenic	1.81e-19	-0.21	7.47e-9	-0.18	3.80e-5	-0.19	8.15e-9

SNP	Chr	Position	Alleles	MAF	Gene	Location	Meta P-value	Arizona		Oklahoma		North/South Dakotas	
								Beta	P-value	Beta	P-value	Beta	P-value
rs11191473	10	104700700	G/C	0.18	CNNM2	intron	1.81e-19	-0.21	7.47e-9	-0.18	3.80e-5	-0.19	8.15e-9
rs7100592	10	104759088	A/C	0.18	CNNM2	intron	1.81e-19	-0.21	7.47e-9	-0.18	3.80e-5	-0.19	8.15e-9
rs12245343	10	104764040	A/G	0.18	CNNM2	intron	1.81e-19	-0.21	7.47e-9	-0.18	3.80e-5	-0.19	8.15e-9
rs74749600	10	104722986	A/G	0.18	CNNM2	intron	1.99e-19	-0.21	9.02e-9	-0.18	4.60e-5	-0.19	5.96e-9
rs12258551	10	104757882	G/C	0.18	CNNM2	intron	2.14e-19	-0.21	7.47e-9	-0.18	3.80e-5	-0.19	9.73e-9
rs77505796	10	104637764	T/A	0.18	AS3MT	intron	3.05e-19	-0.20	1.50e-8	-0.18	4.60e-5	-0.19	5.96e-9
rs17787717	10	104685689	A/C	0.18	CNNM2	intron	3.05e-19	-0.20	1.50e-8	-0.18	4.60e-5	-0.19	5.96e-9
rs75219158	10	104737584	G/A	0.18	CNNM2	intron	3.05e-19	-0.20	1.50e-8	-0.18	4.60e-5	-0.19	5.96e-9
rs11191490	10	104735164	T/A	0.18	CNNM2	intron	3.78e-19	-0.20	1.81e-8	-0.18	3.80e-5	-0.19	8.15e-9
rs284841	10	104540069	A/G	0.20	C10orf26	intron	3.92e-19	0.26	2.56e-14	0.11	2.63e-3	0.18	4.14e-7
rs284844	10	104544519	G/A	0.21	C10orf26	intron	3.92e-19	0.26	2.56e-14	0.11	2.63e-3	0.18	4.14e-7
rs11191439	10	104628713	A/G	0.18	AS3MT	coding	4.37e-19	-0.21	6.45e-9	-0.18	3.90e-5	-0.18	2.26e-8
rs79331374	10	104872903	G/A	0.17	NT5C2	intron	4.76e-19	-0.21	3.33e-8	-0.18	3.00e-5	-0.19	8.15e-9
rs78893207	10	104786128	A/G	0.17	CNNM2	intron	5.15e-19	-0.21	3.33e-8	-0.18	3.20e-5	-0.19	8.15e-9
rs11191545	10	104819783	G/A	0.17	CNNM2	intron	5.53e-19	-0.21	3.33e-8	-0.18	3.40e-5	-0.19	8.15e-9
rs11191523	10	104782390	A/G	0.18	CNNM2	intron	6.33e-19	-0.21	3.33e-8	-0.18	3.80e-5	-0.19	8.15e-9
rs11191525	10	104783425	C/G	0.18	CNNM2	intron	6.33e-19	-0.21	3.33e-8	-0.18	3.80e-5	-0.19	8.15e-9
rs58317752	10	104789257	A/T	0.18	CNNM2	intron	6.33e-19	-0.21	3.33e-8	-0.18	3.80e-5	-0.19	8.15e-9
rs77827514	10	104811999	G/A	0.18	CNNM2	intron	6.33e-19	-0.21	3.33e-8	-0.18	3.80e-5	-0.19	8.15e-9

SNP	Chr	Position	Alleles	MAF	Gene	Location	Meta P-value	Arizona		Oklahoma		North/South Dakotas	
								Beta	P-value	Beta	P-value	Beta	P-value
rs78214351	10	104812678	A/G	0.18	CNNM2	intron	6.33e-19	-0.21	3.33e-8	-0.18	3.80e-5	-0.19	8.15e-9
rs12257941	10	104823151	G/A	0.18	CNNM2	intron	6.33e-19	-0.21	3.33e-8	-0.18	3.80e-5	-0.19	8.15e-9
rs76255497	10	104641945	A/G	0.18	AS3MT	intron	8.25e-19	-0.21	7.47e-9	-0.16	1.28e-4	-0.19	8.15e-9
rs12253284	10	104655135	C/G	0.18	AS3MT, CNNM2	intergenic	8.25e-19	-0.21	7.47e-9	-0.16	1.28e-4	-0.19	8.15e-9
rs12261040	10	104655387	A/G	0.18	AS3MT, CNNM2	intergenic	8.25e-19	-0.21	7.47e-9	-0.16	1.28e-4	-0.19	8.15e-9
rs10509758	10	104671700	A/G	0.18	CNNM2	intron	8.25e-19	-0.21	7.47e-9	-0.16	1.28e-4	-0.19	8.15e-9
rs4919694	10	104688968	A/G	0.18	CNNM2	intron	8.25e-19	-0.21	7.47e-9	-0.16	1.28e-4	-0.19	8.15e-9
rs12268849	10	104727672	G/A	0.18	CNNM2	intron	8.25e-19	-0.21	7.47e-9	-0.16	1.28e-4	-0.19	8.15e-9
rs17727391	10	104826091	C/A	0.18	CNNM2	intron	9.43e-19	-0.21	3.33e-8	-0.18	3.80e-5	-0.18	1.25e-8
rs17727044	10	104811246	T/A	0.17	CNNM2	intron	1.08e-18	-0.20	6.62e-8	-0.18	4.60e-5	-0.19	5.96e-9
rs4917384	10	104985778	G/A	0.26	LOC401648, LOC729020	intergenic	1.20e-18	0.25	1.60e-13	0.12	5.54e-4	0.14	3.36e-6
rs12252500	10	104784974	G/C	0.17	CNNM2	intron	1.47e-18	-0.21	3.33e-8	-0.17	7.60e-5	-0.19	8.15e-9
rs12257935	10	104793052	C/A	0.17	CNNM2	intron	1.47e-18	-0.21	3.33e-8	-0.17	7.60e-5	-0.19	8.15e-9
rs12266291	10	104796874	G/A	0.17	CNNM2	intron	1.47e-18	-0.21	3.33e-8	-0.17	7.60e-5	-0.19	8.15e-9
rs58700372	10	104790195	A/G	0.17	CNNM2	intron	1.82e-18	-0.21	3.33e-8	-0.17	9.00e-5	-0.19	8.15e-9
rs12241091	10	104798987	G/A	0.17	CNNM2	intron	1.82e-18	-0.21	3.33e-8	-0.17	9.00e-5	-0.19	8.15e-9

SNP	Chr	Position	Alleles	MAF	Gene	Location	Meta P-value	Arizona		Oklahoma		North/South Dakotas	
								Beta	P-value	Beta	P-value	Beta	P-value
rs75691516	10	104642035	G/A	0.18	AS3MT	intron	2.26e-18	-0.21	7.47e-9	-0.16	2.77e-4	-0.19	8.15e-9
rs1926032	10	104819459	G/A	0.18	CNNM2	intron	4.59e-18	0.27	1.34e-14	0.09	0.01	0.20	2.19e-7
rs143910868	10	104625749	G/A	0.19	AS3MT	intron	9.43e-18	-0.20	3.25e-8	-0.17	3.40e-5	-0.17	1.63e-7
rs284861	10	104562266	G/A	0.15	C10orf26	intron	1.46e-17	0.26	8.45e-10	0.15	7.41e-4	0.20	8.75e-8
rs284851	10	104568521	G/C	0.15	C10orf26, CYP17A1	intergenic	1.46e-17	0.26	8.45e-10	0.15	7.41e-4	0.20	8.75e-8
rs12261294	10	104924942	G/A	0.19	LOC729081	intron	1.91e-16	-0.20	4.58e-8	-0.14	5.77e-4	-0.17	7.42e-8
rs12262258	10	104890262	G/A	0.19	NT5C2	intron	3.92e-16	-0.20	1.23e-7	-0.14	6.71e-4	-0.17	5.51e-8
rs12243903	10	104896982	A/G	0.19	NT5C2	intron	3.92e-16	-0.20	1.23e-7	-0.14	6.71e-4	-0.17	5.51e-8
rs4917995	10	104835433	T/A	0.19	CNNM2, NT5C2	intergenic	4.96e-16	-0.20	1.23e-7	-0.13	8.02e-4	-0.17	5.51e-8
rs4919685	10	104577352	C/A	0.25	C10orf26, CYP17A1	intergenic	5.25e-16	-0.18	1.68e-7	-0.15	3.00e-5	-0.14	2.69e-6
rs12240508	10	104929222	G/A	0.19	LOC729081, NT5C2	intergenic	5.34e-16	-0.20	9.60e-8	-0.13	9.90e-4	-0.17	5.45e-8
rs743575	10	104584896	A/C	0.25	CYP17A1	intron	6.98e-16	-0.18	1.78e-7	-0.14	8.60e-5	-0.15	1.06e-6
rs4919687	10	104585238	G/A	0.25	CYP17A1	intron	6.98e-16	-0.18	1.78e-7	-0.14	8.60e-5	-0.15	1.06e-6
rs4919686	10	104582239	A/C	0.25	CYP17A1	intron	8.32e-16	-0.18	3.21e-7	-0.15	3.50e-5	-0.14	2.13e-6
rs12416687	10	104619001	A/G	0.24	C10orf32, AS3MT	intergenic	9.26e-16	-0.18	1.85e-7	-0.14	1.75e-4	-0.15	5.89e-7
rs9527	10	104613568	G/A	0.23	C10orf32	UTR	9.67e-16	-0.18	1.95e-7	-0.14	1.75e-4	-0.15	5.89e-7

SNP	Chr	Position	Alleles	MAF	Gene	Location	Meta P-value	Arizona		Oklahoma		North/South Dakotas	
								Beta	P-value	Beta	P-value	Beta	P-value
rs10883783	10	104581142	T/A	0.25	CYP17A1	intron	1.69e-15	-0.18	1.68e-7	-0.14	8.60e-5	-0.14	2.69e-6
rs12255761	10	104791856	G/A	0.19	CNNM2	intron	2.43e-15	-0.20	1.23e-7	-0.13	1.31e-3	-0.16	1.55e-7
rs1475642	10	104536173	A/G	0.24	C10orf26	intron	2.63e-15	-0.16	3.19e-6	-0.17	1.20e-5	-0.14	2.88e-6
rs4919690	10	104606490	A/G	0.24	C10orf32	intron	2.85e-15	-0.18	1.78e-7	-0.13	4.53e-4	-0.15	5.89e-7
rs11191381	10	104483434	G/A	0.24	SFXN2	intron	6.64e-15	-0.17	1.93e-6	-0.15	7.50e-5	-0.14	1.62e-6
rs11191385	10	104503039	C/A	0.24	C10orf26	intron	7.82e-15	-0.17	1.93e-6	-0.15	8.70e-5	-0.14	1.62e-6
rs12776506	10	104522818	A/G	0.25	C10orf26	intron	1.16e-14	-0.16	3.14e-6	-0.15	8.30e-5	-0.14	1.66e-6
rs12256506	10	104953608	A/T	0.19	NT5C2, LOC401648	intergenic	1.23e-14	-0.17	4.89e-6	-0.13	8.64e-4	-0.17	5.51e-8
rs34416174	10	104533878	C/G	0.25	C10orf26	intron	1.26e-14	-0.16	6.06e-6	-0.16	3.90e-5	-0.14	2.31e-6
rs10786667	10	104209122	A/G	0.46	C10orf95, TMEM180	intergenic	1.49e-14	0.16	2.08e-8	0.10	8.98e-4	0.12	7.06e-6
rs56254970	10	104209283	A/G	0.46	C10orf95, TMEM180	intergenic	1.49e-14	0.16	2.08e-8	0.10	8.98e-4	0.12	7.06e-6
rs674816	10	104530555	C/G	0.39	C10orf26	intron	7.88e-14	0.20	1.55e-11	0.10	1.47e-3	0.09	1.09e-3
rs631381	10	104533378	T/A	0.39	C10orf26	intron	1.02e-13	0.20	9.25e-12	0.10	2.00e-3	0.09	1.23e-3
rs12765459	10	104455677	C/G	0.25	ARL3	intron	1.17e-13	-0.16	3.81e-6	-0.15	6.80e-5	-0.12	1.80e-5
rs541393	10	104528601	G/A	0.39	C10orf26	intron	1.20e-13	0.20	1.30e-11	0.10	1.51e-3	0.09	1.57e-3
rs524430	10	104531490	A/C	0.39	C10orf26	intron	1.58e-13	0.20	1.44e-11	0.10	1.78e-3	0.09	1.57e-3
rs593410	10	104522822	A/G	0.39	C10orf26	intron	1.60e-13	0.20	1.54e-11	0.10	1.75e-3	0.09	1.57e-3

SNP	Chr	Position	Alleles	MAF	Gene	Location	Meta P-value	Arizona		Oklahoma		North/South Dakotas	
								Beta	P-value	Beta	P-value	Beta	P-value
rs549466	10	104522903	A/G	0.39	C10orf26	intron	1.60e-13	0.20	1.54e-11	0.10	1.75e-3	0.09	1.57e-3
rs553258	10	104523312	G/A	0.39	C10orf26	intron	1.60e-13	0.20	1.54e-11	0.10	1.75e-3	0.09	1.57e-3
rs499770	10	104524897	G/C	0.39	C10orf26	intron	1.60e-13	0.20	1.54e-11	0.10	1.75e-3	0.09	1.57e-3
rs2254093	10	104529907	C/A	0.39	C10orf26	intron	1.60e-13	0.20	1.54e-11	0.10	1.75e-3	0.09	1.57e-3
rs2482498	10	104525440	G/C	0.39	C10orf26	intron	1.61e-13	0.20	1.55e-11	0.10	1.75e-3	0.09	1.57e-3
rs630185	10	104527139	G/A	0.39	C10orf26	intron	1.61e-13	0.20	1.55e-11	0.10	1.75e-3	0.09	1.57e-3
rs2254111	10	104529510	A/T	0.39	C10orf26	intron	1.61e-13	0.20	1.55e-11	0.10	1.75e-3	0.09	1.57e-3
rs2253703	10	104532964	A/G	0.39	C10orf26	intron	1.61e-13	0.20	1.55e-11	0.10	1.75e-3	0.09	1.57e-3
rs77308618	10	104405811	A/G	0.26	TRIM8	intron	1.62e-13	-0.18	3.01e-7	-0.12	5.71e-4	-0.12	1.80e-5
rs34269636	10	104400589	A/G	0.24	TRIM8	intron	1.88e-13	-0.19	1.97e-7	-0.13	7.74e-4	-0.13	2.00e-5
rs2253983	10	104530451	A/G	0.39	C10orf26	intron	2.22e-13	0.20	1.54e-11	0.09	2.25e-3	0.09	1.57e-3
rs2254563	10	104526355	C/G	0.39	C10orf26	intron	2.63e-13	0.20	1.47e-11	0.09	2.61e-3	0.09	1.57e-3
rs10883790	10	104630945	A/C	0.24	AS3MT	intron	2.89e-13	-0.17	6.53e-7	-0.12	1.74e-3	-0.14	4.44e-6
rs7923415	10	104408646	G/A	0.40	TRIM8, ARL3	intergenic	2.96e-13	0.18	9.90e-10	0.10	1.15e-3	0.10	5.21e-4
rs3740392	10	104626845	A/G	0.24	AS3MT	intron	3.28e-13	-0.17	7.67e-7	-0.12	1.74e-3	-0.14	4.44e-6
rs12249194	10	104633688	A/G	0.24	AS3MT	intron	3.44e-13	-0.17	6.53e-7	-0.11	1.99e-3	-0.14	4.44e-6
rs141653928	10	104721009	G/A	0.03	CNNM2	intron	4.39e-13	-0.31	2.14e-4	-0.66	9.12e-6	-0.39	1.20e-5
rs2273555	10	104117161	A/G	0.45	GBF1	intron	6.76e-13	-0.12	5.90e-5	-0.11	3.57e-4	-0.14	1.38e-6

SNP	Chr	Position	Alleles	MAF	Gene	Location	Meta P-value	Arizona		Oklahoma		North/South Dakotas	
								Beta	P-value	Beta	P-value	Beta	P-value
rs12764219	10	104388885	C/A	0.24	SUFU, TRIM8	intergenic	7.37e-13	-0.19	1.95e-7	-0.11	2.35e-3	-0.13	2.00e-5
rs11191371	10	104449887	C/G	0.26	ARL3	intron	7.84e-13	-0.16	4.35e-6	-0.13	3.99e-4	-0.12	1.60e-5
rs12241712	10	104398262	C/A	0.25	TRIM8	intron	8.86e-13	-0.18	1.62e-7	-0.11	1.89e-3	-0.12	3.60e-5
rs142298786	10	104405084	A/C	0.25	TRIM8	intron	8.97e-13	-0.18	1.65e-7	-0.11	1.89e-3	-0.12	3.60e-5
rs3850699	10	104404211	A/G	0.25	TRIM8	intron	8.97e-13	-0.18	1.65e-7	-0.11	1.89e-3	-0.12	3.60e-5
rs7900450	10	104446300	G/A	0.26	ARL3	intron	1.05e-12	-0.16	4.49e-6	-0.13	2.97e-4	-0.12	2.90e-5
rs7905481	10	104466282	A/G	0.26	SFXN2	intron	1.14e-12	-0.16	4.63e-6	-0.12	6.36e-4	-0.13	1.30e-5
rs80288225	10	104248073	A/G	0.24	ACTR1A	intron	1.34e-12	-0.17	2.35e-6	-0.12	1.33e-3	-0.13	1.10e-5
rs34058856	10	104359313	G/A	0.23	SUFU	intron	1.39e-12	-0.18	1.09e-6	-0.12	1.05e-3	-0.12	2.80e-5
rs35485301	10	104360817	G/A	0.23	SUFU	intron	1.39e-12	-0.18	1.09e-6	-0.12	1.05e-3	-0.12	2.80e-5
rs12773600	10	104408218	C/G	0.25	TRIM8, ARL3	intergenic	1.45e-12	-0.16	8.57e-6	-0.13	3.43e-4	-0.12	2.00e-5
rs12784517	10	104454960	A/G	0.26	ARL3	intron	1.49e-12	-0.16	4.63e-6	-0.13	3.99e-4	-0.12	2.90e-5
rs12761195	10	104455364	A/G	0.26	ARL3	intron	1.49e-12	-0.16	4.63e-6	-0.13	3.99e-4	-0.12	2.90e-5
rs77060053	10	104207743	A/G	0.18	C10orf95, TMEM180	intergenic	1.56e-12	-0.18	3.10e-6	-0.13	2.44e-3	-0.15	4.59e-6
rs7907503	10	104430040	G/A	0.26	ARL3	intron	1.64e-12	-0.16	4.73e-6	-0.13	4.27e-4	-0.12	2.90e-5
rs12783444	10	104470649	G/A	0.26	SFXN2	intron	1.84e-12	-0.16	4.63e-6	-0.12	6.14e-4	-0.12	2.20e-5
rs17784294	10	104469375	C/A	0.26	SFXN2	intron	1.84e-12	-0.16	4.63e-6	-0.12	6.14e-4	-0.12	2.20e-5

SNP	Chr	Position	Alleles	MAF	Gene	Location	Meta P-value	Arizona		Oklahoma		North/South Dakotas	
								Beta	P-value	Beta	P-value	Beta	P-value
rs12779263	10	104876523	C/A	0.25	NT5C2	intron	2.12e-12	-0.17	2.57e-6	-0.10	4.92e-3	-0.14	2.78e-6
rs12780827	10	104457944	A/G	0.26	ARL3	intron	2.41e-12	-0.16	4.63e-6	-0.12	6.14e-4	-0.12	2.90e-5
rs4917999	10	104953041	G/C	0.25	NT5C2, LOC401648	intergenic	2.71e-12	-0.17	2.57e-6	-0.10	5.53e-3	-0.14	3.06e-6
rs4917991	10	104773823	A/G	0.26	CNNM2	intron	3.77e-12	-0.17	4.70e-7	-0.10	4.69e-3	-0.12	2.20e-5
rs182441718	10	104924388	A/G	0.25	NT5C2	intron	3.83e-12	-0.17	2.43e-6	-0.10	7.58e-3	-0.14	2.94e-6
rs7094325	10	104419736	G/C	0.26	TRIM8, ARL3	intergenic	4.87e-12	-0.16	5.43e-6	-0.12	8.65e-4	-0.12	3.50e-5
rs12773833	10	104408340	G/A	0.26	TRIM8, ARL3	intergenic	5.56e-12	-0.16	6.41e-6	-0.12	8.65e-4	-0.12	3.50e-5
rs7898770	10	104746626	G/A	0.26	CNNM2	intron	5.70e-12	-0.17	1.03e-6	-0.10	4.29e-3	-0.12	2.10e-5
rs10883820	10	104754651	C/A	0.26	CNNM2	intron	5.70e-12	-0.17	1.03e-6	-0.10	4.29e-3	-0.12	2.10e-5
rs10509764	10	104766465	G/A	0.26	CNNM2	intron	5.70e-12	-0.17	1.03e-6	-0.10	4.29e-3	-0.12	2.10e-5
rs6584537	10	104768257	G/C	0.26	CNNM2	intron	5.70e-12	-0.17	1.03e-6	-0.10	4.29e-3	-0.12	2.10e-5
rs12570611	10	104411669	G/C	0.26	TRIM8, ARL3	intergenic	5.79e-12	-0.16	6.74e-6	-0.12	8.65e-4	-0.12	3.50e-5
rs7894959	10	104746172	G/A	0.26	CNNM2	intron	5.96e-12	-0.17	1.03e-6	-0.10	4.29e-3	-0.12	2.20e-5
rs12763720	10	104231973	A/G	0.23	ACTR1A	intron	5.98e-12	-0.18	1.09e-6	-0.11	2.61e-3	-0.12	4.00e-5
rs10883818	10	104749576	A/G	0.26	CNNM2	intron	6.41e-12	-0.17	1.03e-6	-0.10	4.69e-3	-0.12	2.10e-5
rs10786732	10	104752525	A/G	0.26	CNNM2	intron	6.41e-12	-0.17	1.03e-6	-0.10	4.69e-3	-0.12	2.10e-5

SNP	Chr	Position	Alleles	MAF	Gene	Location	Meta P-value	Arizona		Oklahoma		North/South Dakotas	
								Beta	P-value	Beta	P-value	Beta	P-value
rs11191513	10	104762974	A/C	0.26	CNNM2	intron	6.41e-12	-0.17	1.03e-6	-0.10	4.69e-3	-0.12	2.10e-5
rs192145925	10	104666944	G/A	0.26	AS3MT, CNNM2	intergenic	7.32e-12	-0.17	4.68e-7	-0.09	7.96e-3	-0.12	2.10e-5
rs76099321	10	104787273	G/A	0.29	CNNM2	intron	7.79e-12	-0.19	1.51e-9	-0.09	0.01	-0.12	4.58e-4
rs78193706	10	104770554	A/G	0.26	CNNM2	intron	7.80e-12	-0.17	1.03e-6	-0.10	5.45e-3	-0.12	2.10e-5
rs10883801	10	104667877	A/C	0.26	AS3MT, CNNM2	intergenic	8.48e-12	-0.17	7.25e-7	-0.10	7.03e-3	-0.12	2.10e-5
rs10883829	10	104830625	A/G	0.26	CNNM2, NT5C2	intergenic	9.95e-12	-0.17	2.37e-6	-0.10	3.92e-3	-0.12	2.20e-5
rs12573077	10	104424620	C/A	0.25	ARL3	UTR	1.01e-11	-0.15	2.30e-5	-0.13	5.79e-4	-0.12	3.50e-5
rs35637354	10	104211844	A/C	0.23	TMEM180	intron	1.06e-11	-0.18	1.09e-6	-0.11	2.84e-3	-0.12	6.40e-5
rs7894588	10	104746020	A/T	0.26	CNNM2	intron	1.11e-11	-0.17	1.03e-6	-0.10	7.08e-3	-0.12	2.10e-5
rs17115317	10	104734215	G/A	0.28	CNNM2	intron	1.16e-11	-0.17	2.22e-8	-0.09	9.03e-3	-0.13	2.17e-4
rs1935323	10	104867025	A/G	0.25	NT5C2	intron	1.18e-11	-0.17	2.31e-6	-0.10	4.69e-3	-0.12	2.10e-5
rs17115414	10	104815142	A/C	0.29	CNNM2	intron	1.20e-11	-0.19	1.51e-9	-0.09	0.01	-0.12	5.91e-4
rs10883800	10	104661202	A/C	0.26	AS3MT, CNNM2	intergenic	1.29e-11	-0.17	1.03e-6	-0.09	7.96e-3	-0.12	2.10e-5
rs7073295	10	104662223	A/G	0.26	AS3MT, CNNM2	intergenic	1.29e-11	-0.17	1.03e-6	-0.09	7.96e-3	-0.12	2.10e-5
rs58289149	10	104770542	G/A	0.26	CNNM2	intron	1.34e-11	-0.17	1.03e-6	-0.09	8.18e-3	-0.12	2.10e-5
rs11191293	10	104211529	G/A	0.42	TMEM180	intron	1.37e-11	0.18	1.63e-9	0.09	3.16e-3	0.08	3.18e-3

SNP	Chr	Position	Alleles	MAF	Gene	Location	Meta P-value	Arizona		Oklahoma		North/South Dakotas	
								Beta	P-value	Beta	P-value	Beta	P-value
rs74233806	10	104836881	G/A	0.29	CNNM2, NT5C2	intergenic	1.39e-11	-0.19	1.51e-9	-0.09	0.01	-0.12	5.70e-4
rs1046411	10	104827806	G/A	0.25	CNNM2	UTR	1.51e-11	-0.16	4.92e-6	-0.11	2.44e-3	-0.12	3.50e-5
rs7096452	10	104726553	G/A	0.26	CNNM2	intron	1.52e-11	-0.17	1.03e-6	-0.09	9.59e-3	-0.12	1.90e-5
rs17725614	10	104675483	G/C	0.26	CNNM2	intron	1.67e-11	-0.17	1.03e-6	-0.09	9.59e-3	-0.12	2.10e-5
rs3897401	10	104686369	A/G	0.26	CNNM2	intron	1.67e-11	-0.17	1.03e-6	-0.09	9.59e-3	-0.12	2.10e-5
rs10883804	10	104696510	G/A	0.26	CNNM2	intron	1.67e-11	-0.17	1.03e-6	-0.09	9.59e-3	-0.12	2.10e-5
rs7089061	10	104713005	C/A	0.26	CNNM2	intron	1.67e-11	-0.17	1.03e-6	-0.09	9.59e-3	-0.12	2.10e-5
rs10883810	10	104717214	A/C	0.26	CNNM2	intron	1.67e-11	-0.17	1.03e-6	-0.09	9.59e-3	-0.12	2.10e-5
rs7899622	10	104717323	G/A	0.26	CNNM2	intron	1.67e-11	-0.17	1.03e-6	-0.09	9.59e-3	-0.12	2.10e-5
rs12248123	10	104725356	A/G	0.26	CNNM2	intron	1.67e-11	-0.17	1.03e-6	-0.09	9.59e-3	-0.12	2.10e-5
rs7080462	10	104726689	A/G	0.26	CNNM2	intron	1.67e-11	-0.17	1.03e-6	-0.09	9.59e-3	-0.12	2.10e-5
rs11191489	10	104730558	C/A	0.26	CNNM2	intron	1.67e-11	-0.17	1.03e-6	-0.09	9.59e-3	-0.12	2.10e-5
rs11191485	10	104719806	C/G	0.26	CNNM2	intron	1.72e-11	-0.17	1.07e-6	-0.09	9.59e-3	-0.12	2.10e-5
rs1926029	10	104845660	G/A	0.25	NT5C2	intron	2.11e-11	-0.16	4.92e-6	-0.10	4.70e-3	-0.12	2.10e-5
rs2274339	10	104850043	A/T	0.25	NT5C2	intron	2.11e-11	-0.16	4.92e-6	-0.10	4.70e-3	-0.12	2.10e-5
rs7912517	10	104895519	G/A	0.25	NT5C2	intron	2.11e-11	-0.16	4.92e-6	-0.10	4.70e-3	-0.12	2.10e-5
rs2066322	10	104899329	A/C	0.25	NT5C2	intron	2.11e-11	-0.16	4.92e-6	-0.10	4.70e-3	-0.12	2.10e-5
rs10786744	10	104935018	C/A	0.25	NT5C2	intron	2.11e-11	-0.16	4.92e-6	-0.10	4.70e-3	-0.12	2.10e-5

SNP	Chr	Position	Alleles	MAF	Gene	Location	Meta P-value	Arizona		Oklahoma		North/South Dakotas	
								Beta	P-value	Beta	P-value	Beta	P-value
rs10883843	10	104937483	G/C	0.25	NT5C2	intron	2.11e-11	-0.16	4.92e-6	-0.10	4.70e-3	-0.12	2.10e-5
rs11191602	10	104944209	G/A	0.25	NT5C2, LOC401648	intergenic	2.11e-11	-0.16	4.92e-6	-0.10	4.70e-3	-0.12	2.10e-5
rs7095304	10	104944785	G/A	0.25	NT5C2, LOC401648	intergenic	2.11e-11	-0.16	4.92e-6	-0.10	4.70e-3	-0.12	2.10e-5
rs7100369	10	104946208	C/A	0.25	NT5C2, LOC401648	intergenic	2.11e-11	-0.16	4.92e-6	-0.10	4.70e-3	-0.12	2.10e-5
rs12246739	10	104777009	C/A	0.23	CNNM2	intron	2.24e-11	-0.18	1.30e-6	-0.11	5.17e-3	-0.12	5.60e-5
rs10883847	10	104948274	G/A	0.25	NT5C2, LOC401648	intergenic	2.30e-11	-0.16	4.92e-6	-0.10	5.04e-3	-0.12	2.10e-5
rs7901197	10	104830423	G/A	0.25	CNNM2, NT5C2	intergenic	2.37e-11	-0.16	4.89e-6	-0.10	5.17e-3	-0.12	2.10e-5
rs11191549	10	104836787	G/A	0.25	CNNM2, NT5C2	intergenic	2.47e-11	-0.16	4.67e-6	-0.10	5.49e-3	-0.12	2.10e-5
rs11191547	10	104833138	G/A	0.25	CNNM2, NT5C2	intergenic	2.57e-11	-0.16	4.92e-6	-0.10	5.49e-3	-0.12	2.10e-5
rs12785223	10	104834001	A/G	0.25	CNNM2, NT5C2	intergenic	2.57e-11	-0.16	4.92e-6	-0.10	5.49e-3	-0.12	2.10e-5
rs10883830	10	104841275	G/A	0.25	NT5C2	intron	2.57e-11	-0.16	4.92e-6	-0.10	5.49e-3	-0.12	2.10e-5
rs11191553	10	104841386	C/A	0.25	NT5C2	intron	2.57e-11	-0.16	4.92e-6	-0.10	5.49e-3	-0.12	2.10e-5
rs7074395	10	104834918	C/A	0.25	CNNM2, NT5C2	intergenic	2.57e-11	-0.16	4.92e-6	-0.10	5.49e-3	-0.12	2.10e-5

SNP	Chr	Position	Alleles	MAF	Gene	Location	Meta P-value	Arizona		Oklahoma		North/South Dakotas	
								Beta	P-value	Beta	P-value	Beta	P-value
rs11191608	10	104951109	A/G	0.25	NT5C2, LOC401648	intergenic	2.66e-11	-0.16	4.92e-6	-0.10	5.28e-3	-0.12	2.30e-5
rs12259163	10	104951467	A/G	0.25	NT5C2, LOC401648	intergenic	2.66e-11	-0.16	4.92e-6	-0.10	5.28e-3	-0.12	2.30e-5
rs7920868	10	104785399	G/A	0.25	CNNM2	intron	2.79e-11	-0.16	4.92e-6	-0.10	5.85e-3	-0.12	2.10e-5
rs12415388	10	104786335	A/G	0.25	CNNM2	intron	2.79e-11	-0.16	4.92e-6	-0.10	5.85e-3	-0.12	2.10e-5
rs7087459	10	104780989	A/G	0.25	CNNM2	intron	3.12e-11	-0.16	4.92e-6	-0.10	6.38e-3	-0.12	2.10e-5
rs1572578	10	104812421	G/C	0.25	CNNM2	intron	3.12e-11	-0.16	4.92e-6	-0.10	6.38e-3	-0.12	2.10e-5
rs11191540	10	104812799	G/C	0.25	CNNM2	intron	3.12e-11	-0.16	4.92e-6	-0.10	6.38e-3	-0.12	2.10e-5
rs7092029	10	104813899	T/A	0.25	CNNM2	intron	3.12e-11	-0.16	4.92e-6	-0.10	6.38e-3	-0.12	2.10e-5
rs943039	10	104815122	G/A	0.25	CNNM2	intron	3.12e-11	-0.16	4.92e-6	-0.10	6.38e-3	-0.12	2.10e-5
rs2182348	10	104862674	A/G	0.25	NT5C2	intron	4.03e-11	-0.16	4.92e-6	-0.09	8.98e-3	-0.13	1.70e-5
rs726010	10	104831967	G/A	0.25	CNNM2, NT5C2	intergenic	4.17e-11	-0.16	4.92e-6	-0.10	7.96e-3	-0.12	2.10e-5
rs1926037	10	104868000	A/C	0.25	NT5C2	intron	5.28e-11	-0.16	5.06e-6	-0.09	9.36e-3	-0.12	2.10e-5
rs2281877	10	104856052	G/A	0.25	NT5C2	intron	5.39e-11	-0.16	4.92e-6	-0.09	9.65e-3	-0.12	2.10e-5
rs10786738	10	104859441	G/A	0.25	NT5C2	intron	5.39e-11	-0.16	4.92e-6	-0.09	9.65e-3	-0.12	2.10e-5
rs6584539	10	104869392	C/G	0.25	NT5C2	intron	5.39e-11	-0.16	4.92e-6	-0.09	9.65e-3	-0.12	2.10e-5
rs10883834	10	104871392	A/G	0.25	NT5C2	intron	5.39e-11	-0.16	4.92e-6	-0.09	9.65e-3	-0.12	2.10e-5
rs1541213	10	104875320	A/G	0.25	NT5C2	intron	5.39e-11	-0.16	4.92e-6	-0.09	9.65e-3	-0.12	2.10e-5

SNP	Chr	Position	Alleles	MAF	Gene	Location	Meta P-value	Arizona		Oklahoma		North/South Dakotas	
								Beta	P-value	Beta	P-value	Beta	P-value
rs10883836	10	104881188	A/G	0.25	NT5C2	intron	5.39e-11	-0.16	4.92e-6	-0.09	9.65e-3	-0.12	2.10e-5
rs34104646	10	104886068	T/A	0.25	NT5C2	intron	5.39e-11	-0.16	4.92e-6	-0.09	9.65e-3	-0.12	2.10e-5
rs11191573	10	104886806	A/G	0.25	NT5C2	intron	5.39e-11	-0.16	4.92e-6	-0.09	9.65e-3	-0.12	2.10e-5
rs12775302	10	104901346	A/G	0.25	NT5C2	intron	5.39e-11	-0.16	4.92e-6	-0.09	9.65e-3	-0.12	2.10e-5
rs10883839	10	104910331	A/G	0.25	NT5C2	intron	5.39e-11	-0.16	4.92e-6	-0.09	9.65e-3	-0.12	2.10e-5
rs10509766	10	104916324	A/G	0.25	NT5C2	intron	5.39e-11	-0.16	4.92e-6	-0.09	9.65e-3	-0.12	2.10e-5
rs2148198	10	104905300	G/A	0.25	NT5C2	intron	5.39e-11	-0.16	4.92e-6	-0.09	9.65e-3	-0.12	2.10e-5
rs7073323	10	104787413	A/C	0.25	CNNM2	intron	5.65e-11	-0.16	4.95e-6	-0.09	9.96e-3	-0.12	2.10e-5
rs7910900	10	104819888	G/A	0.25	CNNM2	intron	5.71e-11	-0.16	4.92e-6	-0.10	7.19e-3	-0.12	3.40e-5
rs10218853	10	104786777	A/C	0.25	CNNM2	intron	6.00e-11	-0.16	5.05e-6	-0.09	9.98e-3	-0.12	2.20e-5
rs77273695	10	104694520	G/A	0.28	CNNM2	intron	6.02e-11	-0.17	3.01e-8	-0.09	0.01	-0.12	4.53e-4
rs7089680	10	104792061	A/G	0.25	CNNM2	intron	6.28e-11	-0.16	4.92e-6	-0.09	0.01	-0.12	2.10e-5
rs12355120	10	104842156	A/C	0.25	NT5C2	intron	6.51e-11	-0.16	4.92e-6	-0.09	0.01	-0.12	2.10e-5
rs3781282	10	104842409	A/G	0.25	NT5C2	intron	6.51e-11	-0.16	4.92e-6	-0.09	0.01	-0.12	2.10e-5
rs3781281	10	104842638	G/A	0.25	NT5C2	intron	6.51e-11	-0.16	4.92e-6	-0.09	0.01	-0.12	2.10e-5
rs186617435	10	104842649	T/A	0.25	NT5C2	intron	6.51e-11	-0.16	4.92e-6	-0.09	0.01	-0.12	2.10e-5
rs10786741	10	104929172	G/A	0.25	LOC729081, NT5C2	intergenic	8.74e-11	-0.16	4.92e-6	-0.09	0.01	-0.12	2.10e-5
rs35195396	10	104918760	A/T	0.25	NT5C2	intron	9.86e-11	-0.16	4.89e-6	-0.09	0.01	-0.12	2.30e-5

SNP	Chr	Position	Alleles	MAF	Gene	Location	Meta P-value	Arizona		Oklahoma		North/South Dakotas	
								Beta	P-value	Beta	P-value	Beta	P-value
rs7067663	10	104873640	A/G	0.25	NT5C2	intron	1.39e-10	-0.16	4.92e-6	-0.09	9.65e-3	-0.12	5.80e-5
rs884825	10	104417606	C/A	0.32	TRIM8, ARL3	intergenic	3.50e-10	0.16	1.35e-7	0.08	0.01	0.10	7.26e-4
rs3961457	10	104419629	A/G	0.33	TRIM8, ARL3	intergenic	9.07e-10	0.16	2.69e-7	0.08	0.02	0.10	8.08e-4
rs74541879	10	104616219	A/G	0.06	C10orf32, AS3MT	intergenic	1.39e-9	0.26	9.31e-8	0.17	0.06	0.20	5.79e-4
rs284849	10	104581172	C/A	0.33	CYP17A1	intron	4.69e-9	-0.15	7.38e-7	-0.06	0.09	-0.11	2.89e-4
rs284848	10	104582115	G/A	0.33	CYP17A1	intron	4.69e-9	-0.15	7.38e-7	-0.06	0.09	-0.11	2.89e-4
rs2486758	10	104587470	A/G	0.33	CYP17A1, C10orf32	intergenic	1.33e-8	-0.15	6.72e-7	-0.06	0.09	-0.10	7.83e-4
rs284859	10	104563007	C/A	0.33	C10orf26	coding	1.61e-8	-0.13	8.76e-6	-0.05	0.11	-0.12	1.28e-4
rs4285804	10	104376299	A/T	0.33	SUFU	intron	2.93e-8	-0.14	2.60e-5	-0.06	0.05	-0.10	3.42e-4
rs2432580	10	104552202	G/A	0.33	C10orf26	intron	3.57e-8	-0.13	1.00e-5	-0.05	0.13	-0.12	2.16e-4
rs4919682	10	104574320	G/A	0.30	C10orf26, CYP17A1	intergenic	3.74e-8	-0.05	0.11	-0.12	5.22e-4	-0.13	1.60e-5
rs145033011	10	104405808	A/G	0.04	TRIM8	intron	5.58e-8	-0.31	9.24e-4	-0.29	3.33e-3	-0.21	1.46e-3
rs11191355	10	104382487	A/G	0.28	SUFU	UTR	7.70e-8	0.15	4.36e-6	0.09	7.60e-3	0.07	0.02
rs11598702	10	104887975	A/G	0.20	NT5C2	intron	8.00e-8	0.22	1.90e-5	0.07	0.07	0.10	8.92e-4
rs10883846	10	104948234	G/A	0.20	NT5C2, LOC401648	intergenic	9.00e-8	0.22	1.90e-5	0.07	0.08	0.10	8.92e-4

SNP	Chr	Position	Alleles	MAF	Gene	Location	Meta P-value	Arizona		Oklahoma		North/South Dakotas	
								Beta	P-value	Beta	P-value	Beta	P-value
rs6892	10	104565860	A/G	0.25	C10orf26	UTR	1.21e-7	-0.17	9.50e-5	-0.07	0.08	-0.14	3.23e-4
rs60459635	10	104490978	A/C	0.28	SFXN2, C10orf26	intergenic	2.69e-7	0.16	3.01e-7	0.07	0.02	0.06	0.06
rs2297450	10	104493164	A/G	0.28	SFXN2, C10orf26	intergenic	2.69e-7	0.16	3.01e-7	0.07	0.02	0.06	0.06
rs11191393	10	104517420	G/C	0.27	C10orf26	intron	3.03e-7	0.16	4.10e-7	0.07	0.02	0.06	0.06
rs72845847	10	104521596	A/G	0.27	C10orf26	intron	3.03e-7	0.16	4.10e-7	0.07	0.02	0.06	0.06
rs1339919	10	104524438	A/G	0.27	C10orf26	intron	3.03e-7	0.16	4.10e-7	0.07	0.02	0.06	0.06
rs11191389	10	104513008	G/A	0.28	C10orf26	intron	3.61e-7	0.16	4.10e-7	0.07	0.03	0.06	0.06
rs4147155	10	104526761	G/A	0.28	C10orf26	intron	3.61e-7	0.16	4.10e-7	0.07	0.03	0.06	0.06
rs4917968	10	104210566	G/A	0.30	C10orf95, TMEM180	intergenic	3.72e-7	0.10	1.29e-3	0.08	0.01	0.10	1.32e-3

Abbreviations: Chr, chromosome. MAF, minor allele frequency. SNP, single nucleotide polymorphism.

All SNP 306 associations are below MetaboChip-wide significance threshold of 3.57e-7 for meta-analysis. Base position according to human genome build 18.

Table S6. Top MetaboChip associations for principal component 1 of arsenic species

SNP	Chr	Position	Alleles	MAF	Gene	Location	Meta P-value	Arizona		Oklahoma		North/South Dakotas	
								Beta	P-value	Beta	P-value	Beta	P-value
rs3740394	10	104624464	A/G	0.18	AS3MT	intron	2.19e-38	5.43	8.98e-12	5.96	4.97e-14	5.57	9.18e-16
rs10509760	10	104624097	A/G	0.18	AS3MT	intron	2.45e-38	5.43	8.98e-12	5.96	4.97e-14	5.54	1.03e-15
rs4917986	10	104620171	A/G	0.18	AS3MT	intron	6.11e-38	5.43	8.98e-12	5.85	1.40e-13	5.57	9.18e-16
rs74376228	10	104667652	C/G	0.18	AS3MT, CNNM2	intergenic	7.74e-37	5.35	2.60e-11	5.97	1.45e-13	5.45	4.00e-15
rs76892505	10	104709280	G/A	0.18	CNNM2	intron	1.50e-36	5.27	5.30e-11	5.99	1.37e-13	5.45	4.00e-15
rs74749600	10	104722986	A/G	0.18	CNNM2	intron	2.10e-36	5.33	3.31e-11	5.88	3.11e-13	5.45	4.00e-15
rs80327774	10	104646661	A/G	0.18	AS3MT	intron	2.16e-36	5.44	1.01e-11	5.72	6.20e-13	5.41	6.84e-15
rs12251035	10	104661449	A/C	0.18	AS3MT, CNNM2	intergenic	2.50e-36	5.44	1.02e-11	5.70	7.10e-13	5.41	6.84e-15
rs11191473	10	104700700	G/C	0.18	CNNM2	intron	2.50e-36	5.44	1.02e-11	5.70	7.10e-13	5.41	6.84e-15
rs7100592	10	104759088	A/C	0.18	CNNM2	intron	2.50e-36	5.44	1.02e-11	5.70	7.10e-13	5.41	6.84e-15
rs12245343	10	104764040	A/G	0.18	CNNM2	intron	2.50e-36	5.44	1.02e-11	5.70	7.10e-13	5.41	6.84e-15
rs10509763	10	104766381	G/A	0.18	CNNM2	intron	3.04e-36	5.44	1.02e-11	5.70	8.61e-13	5.41	6.84e-15
rs77505796	10	104637764	T/A	0.18	AS3MT	intron	3.38e-36	5.27	5.30e-11	5.88	3.11e-13	5.45	4.00e-15
rs17787717	10	104685689	A/C	0.18	CNNM2	intron	3.38e-36	5.27	5.30e-11	5.88	3.11e-13	5.45	4.00e-15
rs75219158	10	104737584	G/A	0.18	CNNM2	intron	3.38e-36	5.27	5.30e-11	5.88	3.11e-13	5.45	4.00e-15

SNP	Chr	Position	Alleles	MAF	Gene	Location	Meta P-value	Arizona		Oklahoma		North/South Dakotas	
								Beta	P-value	Beta	P-value	Beta	P-value
rs12258551	10	104757882	G/C	0.18	CNNM2	intron	3.48e-36	5.44	1.02e-11	5.70	7.10e-13	5.38	9.58e-15
rs75691516	10	104642035	G/A	0.18	AS3MT	intron	4.01e-36	5.44	1.02e-11	5.68	1.13e-12	5.41	6.84e-15
rs76255497	10	104641945	A/G	0.18	AS3MT	intron	6.82e-36	5.44	1.02e-11	5.58	1.91e-12	5.41	6.84e-15
rs12253284	10	104655135	C/G	0.18	AS3MT, CNNM2	intergenic	6.82e-36	5.44	1.02e-11	5.58	1.91e-12	5.41	6.84e-15
rs12261040	10	104655387	A/G	0.18	AS3MT, CNNM2	intergenic	6.82e-36	5.44	1.02e-11	5.58	1.91e-12	5.41	6.84e-15
rs10509758	10	104671700	A/G	0.18	CNNM2	intron	6.82e-36	5.44	1.02e-11	5.58	1.91e-12	5.41	6.84e-15
rs4919694	10	104688968	A/G	0.18	CNNM2	intron	6.82e-36	5.44	1.02e-11	5.58	1.91e-12	5.41	6.84e-15
rs12268849	10	104727672	G/A	0.18	CNNM2	intron	6.82e-36	5.44	1.02e-11	5.58	1.91e-12	5.41	6.84e-15
rs79331374	10	104872903	G/A	0.17	NT5C2	intron	6.95e-36	5.47	5.29e-11	5.79	3.83e-13	5.41	6.84e-15
rs11191439	10	104628713	A/G	0.18	AS3MT	coding	9.14e-36	5.46	8.45e-12	5.69	8.23e-13	5.26	2.64e-14
rs11191490	10	104735164	T/A	0.18	CNNM2	intron	9.79e-36	5.30	4.04e-11	5.70	7.10e-13	5.41	6.84e-15
rs11191545	10	104819783	G/A	0.17	CNNM2	intron	1.02e-35	5.47	5.29e-11	5.73	5.65e-13	5.41	6.84e-15
rs11191523	10	104782390	A/G	0.18	CNNM2	intron	1.28e-35	5.47	5.29e-11	5.70	7.10e-13	5.41	6.84e-15
rs11191525	10	104783425	C/G	0.18	CNNM2	intron	1.28e-35	5.47	5.29e-11	5.70	7.10e-13	5.41	6.84e-15
rs58317752	10	104789257	A/T	0.18	CNNM2	intron	1.28e-35	5.47	5.29e-11	5.70	7.10e-13	5.41	6.84e-15
rs77827514	10	104811999	G/A	0.18	CNNM2	intron	1.28e-35	5.47	5.29e-11	5.70	7.10e-13	5.41	6.84e-15
rs78214351	10	104812678	A/G	0.18	CNNM2	intron	1.28e-35	5.47	5.29e-11	5.70	7.10e-13	5.41	6.84e-15

SNP	Chr	Position	Alleles	MAF	Gene	Location	Meta P-value	Arizona		Oklahoma		North/South Dakotas	
								Beta	P-value	Beta	P-value	Beta	P-value
rs12257941	10	104823151	G/A	0.18	CNNM2	intron	1.28e-35	5.47	5.29e-11	5.70	7.10e-13	5.41	6.84e-15
rs78893207	10	104786128	A/G	0.17	CNNM2	intron	1.56e-35	5.47	5.29e-11	5.70	8.61e-13	5.41	6.84e-15
rs17727044	10	104811246	T/A	0.17	CNNM2	intron	1.83e-35	5.28	2.74e-10	5.88	3.11e-13	5.45	4.00e-15
rs17727391	10	104826091	C/A	0.18	CNNM2	intron	2.06e-35	5.47	5.29e-11	5.70	7.10e-13	5.34	1.11e-14
rs4919685	10	104577352	C/A	0.25	C10orf26, CYP17A1	intergenic	3.34e-35	4.89	2.49e-10	5.14	6.07e-14	4.77	4.15e-14
rs3824754	10	104604340	G/A	0.19	C10orf32	intron	3.74e-35	-6.05	3.81e-15	-5.01	9.48e-13	-5.12	6.10e-11
rs11191416	10	104594906	A/C	0.19	CYP17A1, C10orf32	intergenic	4.86e-35	-6.05	3.81e-15	-4.98	1.23e-12	-5.12	6.10e-11
rs11191425	10	104615960	G/A	0.19	C10orf32, AS3MT	intergenic	4.86e-35	-6.05	3.81e-15	-4.98	1.23e-12	-5.12	6.10e-11
rs17878846	10	104620402	T/A	0.19	AS3MT	intron	4.94e-35	-6.02	5.31e-15	-5.01	9.48e-13	-5.12	6.10e-11
rs4919686	10	104582239	A/C	0.25	CYP17A1	intron	6.13e-35	4.70	1.40e-9	5.28	2.76e-14	4.81	2.57e-14
rs10883783	10	104581142	T/A	0.25	CYP17A1	intron	7.35e-35	4.89	2.49e-10	5.06	1.39e-13	4.77	4.15e-14
rs12416687	10	104619001	A/G	0.24	C10orf32, AS3MT	intergenic	1.46e-34	4.91	2.71e-10	5.14	1.57e-13	4.76	6.75e-14
rs58700372	10	104790195	A/G	0.17	CNNM2	intron	1.52e-34	5.47	5.29e-11	5.50	8.17e-12	5.41	6.84e-15
rs12241091	10	104798987	G/A	0.17	CNNM2	intron	1.52e-34	5.47	5.29e-11	5.50	8.17e-12	5.41	6.84e-15
rs143910868	10	104625749	G/A	0.19	AS3MT	intron	1.57e-34	5.17	7.92e-11	5.47	2.27e-12	5.24	1.80e-14
rs12252500	10	104784974	G/C	0.17	CNNM2	intron	1.84e-34	5.47	5.29e-11	5.49	9.86e-12	5.41	6.84e-15

SNP	Chr	Position	Alleles	MAF	Gene	Location	Meta P-value	Arizona		Oklahoma		North/South Dakotas	
								Beta	P-value	Beta	P-value	Beta	P-value
rs12257935	10	104793052	C/A	0.17	CNNM2	intron	1.84e-34	5.47	5.29e-11	5.49	9.86e-12	5.41	6.84e-15
rs12266291	10	104796874	G/A	0.17	CNNM2	intron	1.84e-34	5.47	5.29e-11	5.49	9.86e-12	5.41	6.84e-15
rs9527	10	104613568	G/A	0.23	C10orf32	UTR	1.94e-34	4.87	3.58e-10	5.14	1.57e-13	4.76	6.75e-14
rs743575	10	104584896	A/C	0.25	CYP17A1	intron	1.96e-34	4.91	2.65e-10	5.06	1.39e-13	4.70	1.04e-13
rs4919687	10	104585238	G/A	0.25	CYP17A1	intron	1.96e-34	4.91	2.65e-10	5.06	1.39e-13	4.70	1.04e-13
rs3740393	10	104626645	G/C	0.21	AS3MT	intron	2.58e-34	-5.75	3.91e-14	-4.92	4.57e-13	-4.77	1.10e-10
rs4919690	10	104606490	A/G	0.24	C10orf32	intron	3.13e-34	4.91	2.65e-10	5.06	3.56e-13	4.76	6.75e-14
rs3740390	10	104628470	G/A	0.19	AS3MT	intron	4.56e-34	-5.96	8.40e-15	-4.84	5.56e-12	-5.10	6.46e-11
rs113282265	10	104647238	A/G	0.19	AS3MT	intron	4.56e-34	-5.96	8.40e-15	-4.84	5.56e-12	-5.10	6.46e-11
rs3824755	10	104585839	C/G	0.19	CYP17A1	intron	5.08e-34	-6.05	3.76e-15	-4.98	1.23e-12	-4.82	4.72e-10
rs1004467	10	104584497	A/G	0.19	CYP17A1	intron	5.08e-34	-6.05	3.76e-15	-4.98	1.23e-12	-4.82	4.72e-10
rs4409766	10	104606653	A/G	0.19	C10orf32	intron	5.44e-34	-6.05	3.76e-15	-4.95	1.32e-12	-4.82	4.72e-10
rs10786736	10	104839106	C/G	0.20	NT5C2	UTR	6.98e-34	-5.64	4.90e-13	-4.78	4.83e-12	-5.22	4.31e-12
rs188299216	10	104944768	G/A	0.19	NT5C2, LOC401648	intergenic	8.33e-34	-5.69	3.18e-13	-4.86	3.19e-12	-5.26	1.08e-11
rs77420391	10	104935813	G/A	0.19	NT5C2	intron	2.40e-33	-5.67	3.69e-13	-4.84	5.25e-12	-5.27	1.61e-11
rs11191582	10	104903643	G/A	0.19	NT5C2	intron	2.40e-33	-5.67	3.69e-13	-4.84	5.25e-12	-5.27	1.61e-11
rs11191447	10	104642313	G/A	0.19	AS3MT	intron	4.51e-33	-5.96	8.40e-15	-4.57	5.18e-11	-5.10	6.46e-11
rs79780963	10	104942489	G/A	0.19	NT5C2	intron	7.79e-33	-5.53	1.10e-12	-4.84	6.01e-12	-5.27	1.72e-11

SNP	Chr	Position	Alleles	MAF	Gene	Location	Meta P-value	Arizona		Oklahoma		North/South Dakotas	
								Beta	P-value	Beta	P-value	Beta	P-value
rs78821730	10	104674534	G/A	0.19	CNNM2	intron	1.03e-32	-5.67	3.69e-13	-4.85	4.99e-12	-5.10	6.46e-11
rs732998	10	104887891	A/G	0.19	NT5C2	intron	1.04e-32	-5.68	3.53e-13	-4.84	5.25e-12	-5.10	6.46e-11
rs79237883	10	104930936	A/G	0.19	LOC729081, NT5C2	intergenic	1.08e-32	-5.67	3.69e-13	-4.84	5.25e-12	-5.10	6.46e-11
rs11191558	10	104854668	G/A	0.19	NT5C2	intron	1.08e-32	-5.67	3.69e-13	-4.84	5.25e-12	-5.10	6.46e-11
rs11191560	10	104859028	A/G	0.19	NT5C2	intron	1.08e-32	-5.67	3.69e-13	-4.84	5.25e-12	-5.10	6.46e-11
rs11191580	10	104896201	A/G	0.19	NT5C2	intron	1.08e-32	-5.67	3.69e-13	-4.84	5.25e-12	-5.10	6.46e-11
rs12220375	10	104891481	A/G	0.19	NT5C2	intron	1.08e-32	-5.67	3.69e-13	-4.84	5.25e-12	-5.10	6.46e-11
rs12413046	10	104861194	A/G	0.19	NT5C2	intron	1.08e-32	-5.67	3.69e-13	-4.84	5.25e-12	-5.10	6.46e-11
rs9633712	10	104863751	G/C	0.19	NT5C2	intron	1.08e-32	-5.67	3.69e-13	-4.84	5.25e-12	-5.10	6.46e-11
rs12412038	10	104846152	G/A	0.19	NT5C2	intron	1.14e-32	-5.67	3.69e-13	-4.84	5.25e-12	-5.10	6.78e-11
rs11191453	10	104649842	A/G	0.19	AS3MT	intron	1.15e-32	-5.67	3.69e-13	-4.84	5.56e-12	-5.10	6.46e-11
rs12221193	10	104655257	A/C	0.19	AS3MT, CNNM2	intergenic	1.15e-32	-5.67	3.69e-13	-4.84	5.56e-12	-5.10	6.46e-11
rs77180047	10	104656747	G/A	0.19	AS3MT, CNNM2	intergenic	1.15e-32	-5.67	3.69e-13	-4.84	5.56e-12	-5.10	6.46e-11
rs17115213	10	104671133	A/G	0.19	CNNM2	intron	1.15e-32	-5.67	3.69e-13	-4.84	5.56e-12	-5.10	6.46e-11
rs10509759	10	104679655	T/A	0.19	CNNM2	intron	1.15e-32	-5.67	3.69e-13	-4.84	5.56e-12	-5.10	6.46e-11
rs5011520	10	104687506	G/A	0.19	CNNM2	intron	1.15e-32	-5.67	3.69e-13	-4.84	5.56e-12	-5.10	6.46e-11
rs10883808	10	104711116	T/A	0.19	CNNM2	intron	1.15e-32	-5.67	3.69e-13	-4.84	5.56e-12	-5.10	6.46e-11

SNP	Chr	Position	Alleles	MAF	Gene	Location	Meta P-value	Arizona		Oklahoma		North/South Dakotas	
								Beta	P-value	Beta	P-value	Beta	P-value
rs10883815	10	104729169	A/G	0.19	CNNM2	intron	1.15e-32	-5.67	3.69e-13	-4.84	5.56e-12	-5.10	6.46e-11
rs11191502	10	104755484	A/C	0.19	CNNM2	intron	1.15e-32	-5.67	3.69e-13	-4.84	5.56e-12	-5.10	6.46e-11
rs11191514	10	104763354	G/A	0.19	CNNM2	intron	1.15e-32	-5.67	3.69e-13	-4.84	5.56e-12	-5.10	6.46e-11
rs77787671	10	104766195	G/A	0.19	CNNM2	intron	1.15e-32	-5.67	3.69e-13	-4.84	5.56e-12	-5.10	6.46e-11
rs75970938	10	104783638	A/G	0.19	CNNM2	intron	1.15e-32	-5.67	3.69e-13	-4.84	5.56e-12	-5.10	6.46e-11
rs11191535	10	104805866	G/A	0.19	CNNM2	intron	1.15e-32	-5.67	3.69e-13	-4.84	5.56e-12	-5.10	6.46e-11
rs10458729	10	104831469	G/A	0.19	CNNM2, NT5C2	intergenic	1.15e-32	-5.67	3.69e-13	-4.84	5.56e-12	-5.10	6.46e-11
rs11191454	10	104649994	A/G	0.19	AS3MT	intron	1.15e-32	-5.67	3.69e-13	-4.84	5.56e-12	-5.10	6.46e-11
rs11191472	10	104697006	T/A	0.19	CNNM2	intron	1.15e-32	-5.67	3.69e-13	-4.84	5.56e-12	-5.10	6.46e-11
rs11191515	10	104766517	G/A	0.19	CNNM2	intron	1.15e-32	-5.67	3.69e-13	-4.84	5.56e-12	-5.10	6.46e-11
rs11191548	10	104836168	A/G	0.19	CNNM2, NT5C2	intergenic	1.15e-32	-5.67	3.69e-13	-4.84	5.56e-12	-5.10	6.46e-11
rs12411886	10	104675289	C/A	0.19	CNNM2	intron	1.15e-32	-5.67	3.69e-13	-4.84	5.56e-12	-5.10	6.46e-11
rs12413409	10	104709086	G/A	0.19	CNNM2	intron	1.15e-32	-5.67	3.69e-13	-4.84	5.56e-12	-5.10	6.46e-11
rs3781285	10	104815655	C/G	0.19	CNNM2	intron	1.15e-32	-5.67	3.69e-13	-4.84	5.56e-12	-5.10	6.46e-11
rs17884001	10	104651235	G/A	0.19	AS3MT	UTR	1.23e-32	-5.67	3.69e-13	-4.81	5.95e-12	-5.10	6.46e-11
rs12220743	10	104841902	G/A	0.19	NT5C2	intron	1.28e-32	-5.67	3.69e-13	-4.83	6.20e-12	-5.10	6.46e-11
rs943037	10	104825909	G/A	0.19	CNNM2	coding	1.28e-32	-5.67	3.69e-13	-4.83	6.20e-12	-5.10	6.46e-11

SNP	Chr	Position	Alleles	MAF	Gene	Location	Meta P-value	Arizona		Oklahoma		North/South Dakotas	
								Beta	P-value	Beta	P-value	Beta	P-value
rs34416174	10	104533878	C/G	0.25	C10orf26	intron	1.36e-32	4.22	5.11e-8	5.22	1.94e-13	4.76	1.68e-14
rs11191479	10	104713610	A/G	0.19	CNNM2	intron	1.40e-32	-5.67	3.69e-13	-4.84	5.56e-12	-5.10	7.73e-11
rs1475642	10	104536173	A/G	0.24	C10orf26	intron	1.44e-32	4.33	2.56e-8	5.18	3.78e-13	4.78	2.13e-14
rs12217501	10	104841879	A/G	0.19	NT5C2	intron	1.60e-32	-5.68	3.53e-13	-4.81	8.10e-12	-5.10	6.46e-11
rs10883832	10	104861269	A/C	0.19	NT5C2	intron	1.68e-32	-5.67	3.69e-13	-4.79	8.14e-12	-5.10	6.46e-11
rs11191555	10	104847513	A/C	0.19	NT5C2	intron	1.69e-32	-5.67	3.69e-13	-4.84	5.25e-12	-5.02	9.68e-11
rs12221064	10	104667116	G/A	0.19	AS3MT, CNNM2	intergenic	2.02e-32	-5.67	3.69e-13	-4.79	9.80e-12	-5.10	6.46e-11
rs1060240	10	104873327	A/G	0.19	NT5C2	intron	2.14e-32	-5.73	2.91e-13	-4.76	1.46e-11	-5.12	5.72e-11
rs12219901	10	104830957	A/G	0.19	CNNM2, NT5C2	intergenic	3.11e-32	-5.67	3.69e-13	-4.75	1.51e-11	-5.10	6.46e-11
rs7098825	10	104618224	A/G	0.17	C10orf32, AS3MT	intergenic	3.80e-32	-6.71	1.25e-13	-5.63	1.06e-11	-5.48	2.47e-10
rs10430665	10	104838420	G/A	0.19	NT5C2	UTR	4.18e-32	-5.65	4.79e-13	-4.76	1.62e-11	-5.10	6.44e-11
rs79668541	10	104783894	G/A	0.19	CNNM2	intron	6.82e-32	-5.50	1.81e-12	-4.80	8.18e-12	-5.10	6.46e-11
rs184992072	10	104846756	A/G	0.19	NT5C2	intron	7.28e-32	-5.68	3.50e-13	-4.78	9.42e-12	-4.99	2.24e-10
rs2482507	10	104551346	G/A	0.21	C10orf26	intron	8.27e-32	-5.36	7.48e-13	-4.56	2.44e-11	-4.87	5.78e-11
rs12776506	10	104522818	A/G	0.25	C10orf26	intron	1.91e-31	4.36	2.61e-8	4.95	2.06e-12	4.71	5.94e-14
rs2297787	10	104670127	T/A	0.20	CNNM2	intron	2.00e-31	-5.52	1.03e-12	-4.68	1.59e-11	-4.94	1.50e-10
rs11191595	10	104933038	A/C	0.19	NT5C2	intron	2.30e-31	-5.42	3.35e-12	-4.76	1.51e-11	-5.10	6.90e-11

SNP	Chr	Position	Alleles	MAF	Gene	Location	Meta P-value	Arizona		Oklahoma		North/South Dakotas	
								Beta	P-value	Beta	P-value	Beta	P-value
rs11191381	10	104483434	G/A	0.24	SFXN2	intron	2.31e-31	4.39	2.03e-8	5.01	1.14e-12	4.64	1.74e-13
rs11191385	10	104503039	C/A	0.24	C10orf26	intron	2.78e-31	4.39	2.03e-8	4.99	1.39e-12	4.64	1.74e-13
rs12261294	10	104924942	G/A	0.19	LOC729081	intron	3.45e-31	5.51	1.85e-11	4.56	1.39e-9	4.90	2.16e-13
rs12262258	10	104890262	G/A	0.19	NT5C2	intron	7.17e-31	5.32	9.36e-11	4.59	1.06e-9	4.94	1.29e-13
rs12243903	10	104896982	A/G	0.19	NT5C2	intron	7.17e-31	5.32	9.36e-11	4.59	1.06e-9	4.94	1.29e-13
rs4917384	10	104985778	G/A	0.26	LOC401648, LOC729020	intergenic	9.84e-31	-5.03	4.15e-11	-4.54	2.46e-13	-3.98	1.10e-9
rs4917995	10	104835433	T/A	0.19	CNNM2, NT5C2	intergenic	1.14e-30	5.32	9.36e-11	4.52	1.63e-9	4.94	1.29e-13
rs12240508	10	104929222	G/A	0.19	LOC729081, NT5C2	intergenic	1.95e-30	5.41	5.61e-11	4.46	4.35e-9	4.95	1.20e-13
rs284841	10	104540069	A/G	0.20	C10orf26	intron	2.16e-30	-5.36	2.10e-12	-4.59	3.00e-11	-4.72	4.19e-10
rs284844	10	104544519	G/A	0.21	C10orf26	intron	2.16e-30	-5.36	2.10e-12	-4.59	3.00e-11	-4.72	4.19e-10
rs12768205	10	104637839	G/A	0.27	AS3MT	intron	1.15e-29	-5.56	1.07e-14	-3.76	1.87e-9	-4.02	2.03e-9
rs10883796	10	104645305	G/A	0.27	AS3MT	intron	1.15e-29	-5.56	1.07e-14	-3.76	1.87e-9	-4.02	2.03e-9
rs10883790	10	104630945	A/C	0.24	AS3MT	intron	2.08e-29	4.62	2.27e-9	4.57	3.00e-11	4.33	7.52e-12
rs12255761	10	104791856	G/A	0.19	CNNM2	intron	2.50e-29	5.32	9.36e-11	4.37	8.25e-9	4.81	5.18e-13
rs12256506	10	104953608	A/T	0.19	NT5C2, LOC401648	intergenic	2.56e-29	4.72	3.60e-9	4.58	1.08e-9	4.94	1.29e-13
rs12249194	10	104633688	A/G	0.24	AS3MT	intron	3.41e-29	4.62	2.27e-9	4.51	4.96e-11	4.33	7.52e-12

SNP	Chr	Position	Alleles	MAF	Gene	Location	Meta P-value	Arizona		Oklahoma		North/South Dakotas	
								Beta	P-value	Beta	P-value	Beta	P-value
rs3740392	10	104626845	A/G	0.24	AS3MT	intron	3.71e-29	4.54	4.04e-9	4.57	3.00e-11	4.33	7.52e-12
rs10786722	10	104650058	G/A	0.27	AS3MT	intron	2.62e-28	-5.23	5.42e-13	-3.76	1.87e-9	-4.02	2.03e-9
rs7897654	10	104652448	A/G	0.27	AS3MT, CNNM2	intergenic	2.62e-28	-5.23	5.42e-13	-3.76	1.87e-9	-4.02	2.03e-9
rs10883799	10	104653300	G/A	0.27	AS3MT, CNNM2	intergenic	2.62e-28	-5.23	5.42e-13	-3.76	1.87e-9	-4.02	2.03e-9
rs1046778	10	104651474	A/G	0.27	AS3MT	UTR	2.62e-28	-5.23	5.42e-13	-3.76	1.87e-9	-4.02	2.03e-9
rs1926032	10	104819459	G/A	0.18	CNNM2	intron	2.07e-27	-5.47	3.22e-12	-4.50	3.80e-10	-4.64	1.47e-8
rs34269636	10	104400589	A/G	0.24	TRIM8	intron	2.18e-27	4.76	2.32e-9	4.39	5.18e-10	4.15	4.75e-11
rs12765002	10	104625338	G/A	0.25	AS3MT	intron	3.27e-27	-5.79	4.57e-15	-3.64	1.18e-8	-3.69	8.27e-8
rs12764049	10	104624946	A/G	0.25	AS3MT	intron	3.33e-27	-5.79	4.57e-15	-3.64	1.20e-8	-3.69	8.27e-8
rs12765459	10	104455677	C/G	0.25	ARL3	intron	3.68e-27	4.30	4.32e-8	4.62	2.60e-11	4.01	7.73e-11
rs4917991	10	104773823	A/G	0.26	CNNM2	intron	4.21e-27	4.78	5.31e-10	3.97	2.26e-9	4.03	8.24e-11
rs12779263	10	104876523	C/A	0.25	NT5C2	intron	4.51e-27	4.68	3.44e-9	3.99	2.69e-9	4.22	1.22e-11
rs4917999	10	104953041	G/C	0.25	NT5C2, LOC401648	intergenic	4.62e-27	4.68	3.44e-9	3.98	3.04e-9	4.22	1.10e-11
rs192145925	10	104666944	G/A	0.26	AS3MT, CNNM2	intergenic	4.96e-27	4.77	5.26e-10	3.94	2.88e-9	4.04	7.62e-11
rs7920697	10	104623327	A/G	0.26	AS3MT	intron	6.11e-27	-5.79	4.57e-15	-3.58	2.17e-8	-3.69	8.02e-8
rs12764219	10	104388885	C/A	0.24	SUFU, TRIM8	intergenic	6.14e-27	4.79	2.07e-9	4.20	1.61e-9	4.15	4.75e-11

SNP	Chr	Position	Alleles	MAF	Gene	Location	Meta P-value	Arizona		Oklahoma		North/South Dakotas	
								Beta	P-value	Beta	P-value	Beta	P-value
rs10883801	10	104667877	A/C	0.26	AS3MT, CNNM2	intergenic	6.18e-27	4.70	1.06e-9	3.99	1.90e-9	4.04	7.62e-11
rs10883818	10	104749576	A/G	0.26	CNNM2	intron	1.29e-26	4.62	1.92e-9	3.97	2.26e-9	4.04	7.62e-11
rs10786732	10	104752525	A/G	0.26	CNNM2	intron	1.29e-26	4.62	1.92e-9	3.97	2.26e-9	4.04	7.62e-11
rs11191513	10	104762974	A/C	0.26	CNNM2	intron	1.29e-26	4.62	1.92e-9	3.97	2.26e-9	4.04	7.62e-11
rs78193706	10	104770554	A/G	0.26	CNNM2	intron	1.53e-26	4.62	1.92e-9	3.95	2.68e-9	4.04	7.62e-11
rs7898770	10	104746626	G/A	0.26	CNNM2	intron	1.53e-26	4.62	1.92e-9	3.96	2.68e-9	4.04	7.62e-11
rs10883820	10	104754651	C/A	0.26	CNNM2	intron	1.53e-26	4.62	1.92e-9	3.96	2.68e-9	4.04	7.62e-11
rs10509764	10	104766465	G/A	0.26	CNNM2	intron	1.53e-26	4.62	1.92e-9	3.96	2.68e-9	4.04	7.62e-11
rs6584537	10	104768257	G/C	0.26	CNNM2	intron	1.53e-26	4.62	1.92e-9	3.96	2.68e-9	4.04	7.62e-11
rs10883800	10	104661202	A/C	0.26	AS3MT, CNNM2	intergenic	1.65e-26	4.62	1.92e-9	3.94	2.88e-9	4.04	7.62e-11
rs7073295	10	104662223	A/G	0.26	AS3MT, CNNM2	intergenic	1.65e-26	4.62	1.92e-9	3.94	2.88e-9	4.04	7.62e-11
rs7894959	10	104746172	G/A	0.26	CNNM2	intron	1.71e-26	4.62	1.92e-9	3.96	2.68e-9	4.03	8.51e-11
rs182441718	10	104924388	A/G	0.25	NT5C2	intron	2.08e-26	4.72	3.09e-9	3.86	1.17e-8	4.22	1.35e-11
rs77308618	10	104405811	A/G	0.26	TRIM8	intron	2.21e-26	4.64	2.20e-9	3.92	7.50e-9	4.09	3.27e-11
rs7096452	10	104726553	G/A	0.26	CNNM2	intron	2.51e-26	4.62	1.92e-9	3.89	4.36e-9	4.03	7.60e-11
rs17725614	10	104675483	G/C	0.26	CNNM2	intron	2.52e-26	4.62	1.92e-9	3.89	4.36e-9	4.04	7.62e-11
rs3897401	10	104686369	A/G	0.26	CNNM2	intron	2.52e-26	4.62	1.92e-9	3.89	4.36e-9	4.04	7.62e-11

SNP	Chr	Position	Alleles	MAF	Gene	Location	Meta P-value	Arizona		Oklahoma		North/South Dakotas	
								Beta	P-value	Beta	P-value	Beta	P-value
rs10883804	10	104696510	G/A	0.26	CNNM2	intron	2.52e-26	4.62	1.92e-9	3.89	4.36e-9	4.04	7.62e-11
rs7089061	10	104713005	C/A	0.26	CNNM2	intron	2.52e-26	4.62	1.92e-9	3.89	4.36e-9	4.04	7.62e-11
rs10883810	10	104717214	A/C	0.26	CNNM2	intron	2.52e-26	4.62	1.92e-9	3.89	4.36e-9	4.04	7.62e-11
rs7899622	10	104717323	G/A	0.26	CNNM2	intron	2.52e-26	4.62	1.92e-9	3.89	4.36e-9	4.04	7.62e-11
rs12248123	10	104725356	A/G	0.26	CNNM2	intron	2.52e-26	4.62	1.92e-9	3.89	4.36e-9	4.04	7.62e-11
rs7080462	10	104726689	A/G	0.26	CNNM2	intron	2.52e-26	4.62	1.92e-9	3.89	4.36e-9	4.04	7.62e-11
rs11191489	10	104730558	C/A	0.26	CNNM2	intron	2.52e-26	4.62	1.92e-9	3.89	4.36e-9	4.04	7.62e-11
rs11191485	10	104719806	C/G	0.26	CNNM2	intron	2.65e-26	4.61	2.02e-9	3.89	4.36e-9	4.04	7.62e-11
rs1935323	10	104867025	A/G	0.25	NT5C2	intron	3.37e-26	4.71	1.98e-9	3.89	5.59e-9	4.04	7.62e-11
rs7894588	10	104746020	A/T	0.26	CNNM2	intron	5.09e-26	4.62	1.92e-9	3.84	8.59e-9	4.04	7.62e-11
rs58289149	10	104770542	G/A	0.26	CNNM2	intron	6.05e-26	4.62	1.92e-9	3.82	1.01e-8	4.04	7.62e-11
rs142298786	10	104405084	A/C	0.25	TRIM8	intron	6.47e-26	4.75	1.04e-9	4.01	5.93e-9	3.99	2.53e-10
rs3850699	10	104404211	A/G	0.25	TRIM8	intron	6.47e-26	4.75	1.04e-9	4.01	5.93e-9	3.99	2.53e-10
rs12241712	10	104398262	C/A	0.25	TRIM8	intron	6.90e-26	4.73	1.11e-9	4.01	5.93e-9	3.99	2.53e-10
rs10883829	10	104830625	A/G	0.26	CNNM2, NT5C2	intergenic	7.07e-26	4.71	2.05e-9	3.82	1.01e-8	4.03	8.41e-11
rs7087459	10	104780989	A/G	0.25	CNNM2	intron	8.66e-26	4.55	7.05e-9	3.90	4.37e-9	4.04	7.62e-11
rs1572578	10	104812421	G/C	0.25	CNNM2	intron	8.66e-26	4.55	7.05e-9	3.90	4.37e-9	4.04	7.62e-11
rs11191540	10	104812799	G/C	0.25	CNNM2	intron	8.66e-26	4.55	7.05e-9	3.90	4.37e-9	4.04	7.62e-11

SNP	Chr	Position	Alleles	MAF	Gene	Location	Meta P-value	Arizona		Oklahoma		North/South Dakotas	
								Beta	P-value	Beta	P-value	Beta	P-value
rs7092029	10	104813899	T/A	0.25	CNNM2	intron	8.66e-26	4.55	7.05e-9	3.90	4.37e-9	4.04	7.62e-11
rs943039	10	104815122	G/A	0.25	CNNM2	intron	8.66e-26	4.55	7.05e-9	3.90	4.37e-9	4.04	7.62e-11
rs7900450	10	104446300	G/A	0.26	ARL3	intron	9.15e-26	4.23	3.29e-8	4.23	4.32e-10	3.92	1.79e-10
rs7907503	10	104430040	G/A	0.26	ARL3	intron	9.79e-26	4.24	3.18e-8	4.15	6.60e-10	3.97	1.31e-10
rs12773600	10	104408218	C/G	0.25	TRIM8, ARL3	intergenic	9.99e-26	4.22	9.64e-8	4.19	1.91e-9	4.18	1.39e-11
rs7920868	10	104785399	G/A	0.25	CNNM2	intron	1.03e-25	4.55	7.05e-9	3.89	5.18e-9	4.04	7.62e-11
rs12415388	10	104786335	A/G	0.25	CNNM2	intron	1.03e-25	4.55	7.05e-9	3.89	5.18e-9	4.04	7.62e-11
rs12780827	10	104457944	A/G	0.26	ARL3	intron	1.06e-25	4.23	3.36e-8	4.21	4.65e-10	3.92	1.90e-10
rs1926029	10	104845660	G/A	0.25	NT5C2	intron	1.13e-25	4.55	7.05e-9	3.89	5.67e-9	4.04	7.62e-11
rs2274339	10	104850043	A/T	0.25	NT5C2	intron	1.13e-25	4.55	7.05e-9	3.89	5.67e-9	4.04	7.62e-11
rs7912517	10	104895519	G/A	0.25	NT5C2	intron	1.13e-25	4.55	7.05e-9	3.89	5.67e-9	4.04	7.62e-11
rs2066322	10	104899329	A/C	0.25	NT5C2	intron	1.13e-25	4.55	7.05e-9	3.89	5.67e-9	4.04	7.62e-11
rs10786744	10	104935018	C/A	0.25	NT5C2	intron	1.13e-25	4.55	7.05e-9	3.89	5.67e-9	4.04	7.62e-11
rs10883843	10	104937483	G/C	0.25	NT5C2	intron	1.13e-25	4.55	7.05e-9	3.89	5.67e-9	4.04	7.62e-11
rs11191602	10	104944209	G/A	0.25	NT5C2, LOC401648	intergenic	1.13e-25	4.55	7.05e-9	3.89	5.67e-9	4.04	7.62e-11
rs7095304	10	104944785	G/A	0.25	NT5C2, LOC401648	intergenic	1.13e-25	4.55	7.05e-9	3.89	5.67e-9	4.04	7.62e-11
rs7100369	10	104946208	C/A	0.25	NT5C2,	intergenic	1.13e-25	4.55	7.05e-9	3.89	5.67e-9	4.04	7.62e-11

SNP	Chr	Position	Alleles	MAF	Gene	Location	Meta P-value	Arizona		Oklahoma		North/South Dakotas	
								Beta	P-value	Beta	P-value	Beta	P-value
					LOC401648								
rs11191608	10	104951109	A/G	0.25	NT5C2, LOC401648	intergenic	1.16e-25	4.55	7.05e-9	3.88	6.35e-9	4.04	6.93e-11
rs12259163	10	104951467	A/G	0.25	NT5C2, LOC401648	intergenic	1.16e-25	4.55	7.05e-9	3.88	6.35e-9	4.04	6.93e-11
rs10883847	10	104948274	G/A	0.25	NT5C2, LOC401648	intergenic	1.26e-25	4.55	7.05e-9	3.88	6.29e-9	4.04	7.62e-11
rs2482498	10	104525440	G/C	0.39	C10orf26	intron	1.29e-25	-4.32	6.26e-11	-3.43	4.99e-9	-3.52	6.31e-9
rs630185	10	104527139	G/A	0.39	C10orf26	intron	1.29e-25	-4.32	6.26e-11	-3.43	4.99e-9	-3.52	6.31e-9
rs2254111	10	104529510	A/T	0.39	C10orf26	intron	1.29e-25	-4.32	6.26e-11	-3.43	4.99e-9	-3.52	6.31e-9
rs2253703	10	104532964	A/G	0.39	C10orf26	intron	1.29e-25	-4.32	6.26e-11	-3.43	4.99e-9	-3.52	6.31e-9
rs12246739	10	104777009	C/A	0.23	CNNM2	intron	1.37e-25	4.84	1.79e-9	4.06	9.27e-9	3.97	2.05e-10
rs593410	10	104522822	A/G	0.39	C10orf26	intron	1.37e-25	-4.31	6.70e-11	-3.43	4.99e-9	-3.52	6.31e-9
rs549466	10	104522903	A/G	0.39	C10orf26	intron	1.37e-25	-4.31	6.70e-11	-3.43	4.99e-9	-3.52	6.31e-9
rs553258	10	104523312	G/A	0.39	C10orf26	intron	1.37e-25	-4.31	6.70e-11	-3.43	4.99e-9	-3.52	6.31e-9
rs499770	10	104524897	G/C	0.39	C10orf26	intron	1.37e-25	-4.31	6.70e-11	-3.43	4.99e-9	-3.52	6.31e-9
rs2254093	10	104529907	C/A	0.39	C10orf26	intron	1.37e-25	-4.31	6.70e-11	-3.43	4.99e-9	-3.52	6.31e-9
rs12783444	10	104470649	G/A	0.26	SFXN2	intron	1.37e-25	4.23	3.36e-8	4.21	4.65e-10	3.91	2.45e-10
rs17784294	10	104469375	C/A	0.26	SFXN2	intron	1.37e-25	4.23	3.36e-8	4.21	4.65e-10	3.91	2.45e-10
rs11191549	10	104836787	G/A	0.25	CNNM2,	intergenic	1.50e-25	4.58	5.93e-9	3.82	8.74e-9	4.04	7.62e-11

SNP	Chr	Position	Alleles	MAF	Gene	Location	Meta P-value	Arizona		Oklahoma		North/South Dakotas	
								Beta	P-value	Beta	P-value	Beta	P-value
					NT5C2								
rs674816	10	104530555	C/G	0.39	C10orf26	intron	1.61e-25	-4.32	6.26e-11	-3.33	1.04e-8	-3.56	3.82e-9
rs524430	10	104531490	A/C	0.39	C10orf26	intron	1.68e-25	-4.33	5.93e-11	-3.40	6.73e-9	-3.52	6.31e-9
rs11191547	10	104833138	G/A	0.25	CNNM2, NT5C2	intergenic	1.76e-25	4.55	7.05e-9	3.82	8.74e-9	4.04	7.62e-11
rs12785223	10	104834001	A/G	0.25	CNNM2, NT5C2	intergenic	1.76e-25	4.55	7.05e-9	3.82	8.74e-9	4.04	7.62e-11
rs10883830	10	104841275	G/A	0.25	NT5C2	intron	1.76e-25	4.55	7.05e-9	3.82	8.74e-9	4.04	7.62e-11
rs11191553	10	104841386	C/A	0.25	NT5C2	intron	1.76e-25	4.55	7.05e-9	3.82	8.74e-9	4.04	7.62e-11
rs7074395	10	104834918	C/A	0.25	CNNM2, NT5C2	intergenic	1.76e-25	4.55	7.05e-9	3.82	8.74e-9	4.04	7.62e-11
rs726010	10	104831967	G/A	0.25	CNNM2, NT5C2	intergenic	1.81e-25	4.55	7.05e-9	3.86	8.94e-9	4.04	7.62e-11
rs12784517	10	104454960	A/G	0.26	ARL3	intron	1.89e-25	4.23	3.36e-8	4.13	8.41e-10	3.92	1.90e-10
rs12761195	10	104455364	A/G	0.26	ARL3	intron	1.89e-25	4.23	3.36e-8	4.13	8.41e-10	3.92	1.90e-10
rs1926037	10	104868000	A/C	0.25	NT5C2	intron	2.03e-25	4.55	7.36e-9	3.83	9.64e-9	4.04	7.62e-11
rs7901197	10	104830423	G/A	0.25	CNNM2, NT5C2	intergenic	2.05e-25	4.55	7.03e-9	3.81	1.01e-8	4.04	7.62e-11
rs2253983	10	104530451	A/G	0.39	C10orf26	intron	2.18e-25	-4.31	6.70e-11	-3.38	7.84e-9	-3.52	6.31e-9
rs2281877	10	104856052	G/A	0.25	NT5C2	intron	2.18e-25	4.55	7.05e-9	3.81	1.07e-8	4.04	7.62e-11
rs10786738	10	104859441	G/A	0.25	NT5C2	intron	2.18e-25	4.55	7.05e-9	3.81	1.07e-8	4.04	7.62e-11

SNP	Chr	Position	Alleles	MAF	Gene	Location	Meta P-value	Arizona		Oklahoma		North/South Dakotas	
								Beta	P-value	Beta	P-value	Beta	P-value
rs6584539	10	104869392	C/G	0.25	NT5C2	intron	2.18e-25	4.55	7.05e-9	3.81	1.07e-8	4.04	7.62e-11
rs10883834	10	104871392	A/G	0.25	NT5C2	intron	2.18e-25	4.55	7.05e-9	3.81	1.07e-8	4.04	7.62e-11
rs1541213	10	104875320	A/G	0.25	NT5C2	intron	2.18e-25	4.55	7.05e-9	3.81	1.07e-8	4.04	7.62e-11
rs10883836	10	104881188	A/G	0.25	NT5C2	intron	2.18e-25	4.55	7.05e-9	3.81	1.07e-8	4.04	7.62e-11
rs34104646	10	104886068	T/A	0.25	NT5C2	intron	2.18e-25	4.55	7.05e-9	3.81	1.07e-8	4.04	7.62e-11
rs11191573	10	104886806	A/G	0.25	NT5C2	intron	2.18e-25	4.55	7.05e-9	3.81	1.07e-8	4.04	7.62e-11
rs12775302	10	104901346	A/G	0.25	NT5C2	intron	2.18e-25	4.55	7.05e-9	3.81	1.07e-8	4.04	7.62e-11
rs10883839	10	104910331	A/G	0.25	NT5C2	intron	2.18e-25	4.55	7.05e-9	3.81	1.07e-8	4.04	7.62e-11
rs10509766	10	104916324	A/G	0.25	NT5C2	intron	2.18e-25	4.55	7.05e-9	3.81	1.07e-8	4.04	7.62e-11
rs2148198	10	104905300	G/A	0.25	NT5C2	intron	2.18e-25	4.55	7.05e-9	3.81	1.07e-8	4.04	7.62e-11
rs2254563	10	104526355	C/G	0.39	C10orf26	intron	2.46e-25	-4.33	5.83e-11	-3.37	9.90e-9	-3.52	6.31e-9
rs11191371	10	104449887	C/G	0.26	ARL3	intron	2.78e-25	4.24	3.04e-8	4.13	8.41e-10	3.87	3.11e-10
rs631381	10	104533378	T/A	0.39	C10orf26	intron	2.90e-25	-4.38	3.46e-11	-3.36	8.10e-9	-3.44	1.33e-8
rs7905481	10	104466282	A/G	0.26	SFXN2	intron	2.97e-25	4.23	3.36e-8	4.21	4.77e-10	3.85	5.18e-10
rs2182348	10	104862674	A/G	0.25	NT5C2	intron	3.16e-25	4.55	7.05e-9	3.88	5.36e-9	3.94	2.32e-10
rs541393	10	104528601	G/A	0.39	C10orf26	intron	3.35e-25	-4.35	5.34e-11	-3.32	1.43e-8	-3.52	6.31e-9
rs12355120	10	104842156	A/C	0.25	NT5C2	intron	3.37e-25	4.55	7.05e-9	3.75	1.63e-8	4.04	7.62e-11
rs3781282	10	104842409	A/G	0.25	NT5C2	intron	3.37e-25	4.55	7.05e-9	3.75	1.63e-8	4.04	7.62e-11
rs3781281	10	104842638	G/A	0.25	NT5C2	intron	3.37e-25	4.55	7.05e-9	3.75	1.63e-8	4.04	7.62e-11

SNP	Chr	Position	Alleles	MAF	Gene	Location	Meta P-value	Arizona		Oklahoma		North/South Dakotas	
								Beta	P-value	Beta	P-value	Beta	P-value
rs186617435	10	104842649	T/A	0.25	NT5C2	intron	3.37e-25	4.55	7.05e-9	3.75	1.63e-8	4.04	7.62e-11
rs1046411	10	104827806	G/A	0.25	CNNM2	UTR	4.35e-25	4.55	7.05e-9	3.82	1.03e-8	3.97	1.63e-10
rs7089680	10	104792061	A/G	0.25	CNNM2	intron	5.28e-25	4.55	7.05e-9	3.74	2.50e-8	4.04	7.62e-11
rs7073323	10	104787413	A/C	0.25	CNNM2	intron	6.35e-25	4.55	7.13e-9	3.72	2.95e-8	4.04	7.62e-11
rs10218853	10	104786777	A/C	0.25	CNNM2	intron	7.01e-25	4.55	7.35e-9	3.72	2.94e-8	4.03	8.22e-11
rs7067663	10	104873640	A/G	0.25	NT5C2	intron	7.82e-25	4.55	7.05e-9	3.81	1.07e-8	3.91	2.83e-10
rs77060053	10	104207743	A/G	0.18	C10orf95, TMEM180	intergenic	8.20e-25	4.69	5.69e-8	4.28	4.55e-8	4.65	7.03e-12
rs10786741	10	104929172	G/A	0.25	LOC729081, NT5C2	intergenic	8.91e-25	4.55	7.05e-9	3.69	4.10e-8	4.04	7.62e-11
rs35195396	10	104918760	A/T	0.25	NT5C2	intron	9.26e-25	4.55	7.03e-9	3.68	3.89e-8	4.03	8.42e-11
rs7094325	10	104419736	G/C	0.26	TRIM8, ARL3	intergenic	1.67e-24	4.25	3.59e-8	3.85	1.73e-8	4.04	7.39e-11
rs34058856	10	104359313	G/A	0.23	SUFU	intron	1.76e-24	4.67	1.16e-8	4.18	4.03e-9	3.87	1.07e-9
rs35485301	10	104360817	G/A	0.23	SUFU	intron	1.76e-24	4.67	1.16e-8	4.18	4.03e-9	3.87	1.07e-9
rs12773833	10	104408340	G/A	0.26	TRIM8, ARL3	intergenic	2.07e-24	4.23	4.47e-8	3.85	1.73e-8	4.04	7.39e-11
rs12570611	10	104411669	G/C	0.26	TRIM8, ARL3	intergenic	2.11e-24	4.22	4.56e-8	3.85	1.73e-8	4.04	7.39e-11
rs7910900	10	104819888	G/A	0.25	CNNM2	intron	2.41e-24	4.55	7.05e-9	3.71	3.51e-8	3.93	2.54e-10
rs284861	10	104562266	G/A	0.15	C10orf26	intron	2.68e-24	-5.60	4.48e-9	-4.37	1.24e-7	-5.06	1.08e-10

SNP	Chr	Position	Alleles	MAF	Gene	Location	Meta P-value	Arizona		Oklahoma		North/South Dakotas	
								Beta	P-value	Beta	P-value	Beta	P-value
rs284851	10	104568521	G/C	0.15	C10orf26, CYP17A1	intergenic	2.68e-24	-5.60	4.48e-9	-4.37	1.24e-7	-5.06	1.08e-10
rs12573077	10	104424620	C/A	0.25	ARL3	UTR	6.05e-24	4.13	2.25e-7	3.95	1.01e-8	4.04	7.39e-11
rs80288225	10	104248073	A/G	0.24	ACTR1A	intron	6.25e-24	4.44	3.28e-8	4.02	7.29e-9	3.83	7.89e-10
rs12763720	10	104231973	A/G	0.23	ACTR1A	intron	6.83e-24	4.67	1.16e-8	4.07	9.66e-9	3.80	1.74e-9
rs35637354	10	104211844	A/C	0.23	TMEM180	intron	9.00e-24	4.67	1.16e-8	4.11	6.11e-9	3.73	3.58e-9
rs10786667	10	104209122	A/G	0.46	C10orf95, TMEM180	intergenic	1.11e-23	-3.40	1.07e-7	-2.95	3.51e-7	-4.03	5.25e-12
rs56254970	10	104209283	A/G	0.46	C10orf95, TMEM180	intergenic	1.11e-23	-3.40	1.07e-7	-2.95	3.51e-7	-4.03	5.25e-12
rs7923415	10	104408646	G/A	0.40	TRIM8, ARL3	intergenic	8.73e-23	-4.16	5.88e-10	-2.88	7.42e-7	-3.56	3.16e-9
rs2273555	10	104117161	A/G	0.45	GBF1	intron	2.60e-22	2.22	6.63e-4	3.12	7.56e-8	4.68	6.56e-15
rs11191293	10	104211529	G/A	0.42	TMEM180	intron	4.47e-20	-3.95	1.90e-9	-2.84	1.12e-6	-3.06	3.36e-7
rs4919682	10	104574320	G/A	0.30	C10orf26, CYP17A1	intergenic	3.73e-17	2.04	3.71e-3	2.64	2.70e-5	4.56	4.52e-13
rs884825	10	104417606	C/A	0.32	TRIM8, ARL3	intergenic	5.36e-17	-3.62	1.55e-7	-2.91	3.93e-6	-3.00	2.52e-6
rs17115317	10	104734215	G/A	0.28	CNNM2	intron	1.49e-16	2.90	2.50e-5	3.07	5.15e-6	4.08	4.47e-8
rs141653928	10	104721009	G/A	0.03	CNNM2	intron	1.60e-16	8.11	1.00e-5	13.19	9.86e-7	9.45	7.09e-7
rs76099321	10	104787273	G/A	0.29	CNNM2	intron	2.68e-16	3.10	6.03e-6	3.06	4.88e-6	3.70	3.95e-7

SNP	Chr	Position	Alleles	MAF	Gene	Location	Meta P-value	Arizona		Oklahoma		North/South Dakotas	
								Beta	P-value	Beta	P-value	Beta	P-value
rs4285804	10	104376299	A/T	0.33	SUFU	intron	3.57e-16	3.71	8.86e-7	2.96	3.83e-6	2.77	3.79e-6
rs17115414	10	104815142	A/C	0.29	CNNM2	intron	4.00e-16	3.10	6.03e-6	3.08	4.43e-6	3.63	6.61e-7
rs74233806	10	104836881	G/A	0.29	CNNM2, NT5C2	intergenic	8.00e-16	3.10	6.03e-6	3.03	6.50e-6	3.61	9.04e-7
rs3961457	10	104419629	A/G	0.33	TRIM8, ARL3	intergenic	1.01e-15	-3.51	3.74e-7	-2.80	1.00e-5	-2.84	8.11e-6
rs77273695	10	104694520	G/A	0.28	CNNM2	intron	3.33e-15	2.80	4.30e-5	3.03	6.87e-6	3.66	5.26e-7
rs11598702	10	104887975	A/G	0.20	NT5C2	intron	4.26e-15	-5.36	2.15e-6	-2.76	1.15e-4	-3.37	5.58e-7
rs10883846	10	104948234	G/A	0.20	NT5C2, LOC401648	intergenic	4.89e-15	-5.36	2.15e-6	-2.72	1.30e-4	-3.37	5.58e-7
rs10786697	10	104368553	A/C	0.33	SUFU	intron	7.59e-14	2.92	1.38e-4	2.77	1.40e-5	2.85	2.01e-6
rs74541879	10	104616219	A/G	0.06	C10orf32, AS3MT	intergenic	8.06e-14	-5.47	3.32e-7	-7.55	5.65e-6	-4.30	6.37e-4
rs10786696	10	104368124	G/A	0.33	SUFU	intron	8.12e-14	2.92	1.38e-4	2.76	1.50e-5	2.85	2.01e-6
rs7087984	10	104370676	A/G	0.33	SUFU	intron	8.12e-14	2.92	1.38e-4	2.76	1.50e-5	2.85	2.01e-6
rs7081467	10	104221652	G/C	0.33	TMEM180	intron	1.13e-13	2.90	1.57e-4	2.91	4.94e-6	2.64	7.37e-6
rs7908249	10	104363290	G/A	0.33	SUFU	intron	1.18e-13	2.92	1.38e-4	2.70	2.20e-5	2.85	2.01e-6
rs7912339	10	104363748	G/C	0.33	SUFU	intron	1.18e-13	2.92	1.38e-4	2.70	2.20e-5	2.85	2.01e-6
rs4604806	10	104363894	A/G	0.33	SUFU	intron	1.18e-13	2.92	1.38e-4	2.70	2.20e-5	2.85	2.01e-6
rs2863716	10	104365726	G/A	0.34	SUFU	intron	1.22e-13	2.93	1.35e-4	2.90	6.27e-6	2.69	7.42e-6

SNP	Chr	Position	Alleles	MAF	Gene	Location	Meta P-value	Arizona		Oklahoma		North/South Dakotas	
								Beta	P-value	Beta	P-value	Beta	P-value
rs7081110	10	104221641	A/G	0.33	TMEM180	intron	1.27e-13	2.91	1.53e-4	2.88	5.95e-6	2.64	7.18e-6
rs12570859	10	104247216	G/A	0.32	ACTR1A	intron	1.36e-13	2.86	1.97e-4	2.95	3.58e-6	2.61	9.47e-6
rs2281880	10	104259207	G/A	0.32	SUFU	intron	1.36e-13	2.86	1.97e-4	2.95	3.58e-6	2.61	9.47e-6
rs12781718	10	104217423	G/A	0.33	TMEM180	intron	1.55e-13	2.91	1.53e-4	2.85	7.34e-6	2.64	7.18e-6
rs11593526	10	104218007	C/A	0.33	TMEM180	intron	1.55e-13	2.91	1.53e-4	2.85	7.34e-6	2.64	7.18e-6
rs11593583	10	104218139	G/A	0.33	TMEM180	intron	1.55e-13	2.91	1.53e-4	2.85	7.34e-6	2.64	7.18e-6
rs41306870	10	104219775	G/A	0.33	TMEM180	coding	1.55e-13	2.91	1.53e-4	2.85	7.34e-6	2.64	7.18e-6
rs2025712	10	104220054	A/G	0.33	TMEM180	intron	1.55e-13	2.91	1.53e-4	2.85	7.34e-6	2.64	7.18e-6
rs11191295	10	104221044	A/G	0.33	TMEM180	coding	1.55e-13	2.91	1.53e-4	2.85	7.34e-6	2.64	7.18e-6
rs3740415	10	104222706	G/A	0.33	TMEM180	intron	1.55e-13	2.91	1.53e-4	2.85	7.34e-6	2.64	7.18e-6
rs74337554	10	104217414	A/G	0.33	TMEM180	intron	1.56e-13	2.91	1.53e-4	2.85	7.34e-6	2.64	7.22e-6
rs2025713	10	104220146	A/G	0.33	TMEM180	intron	1.59e-13	2.90	1.57e-4	2.85	7.34e-6	2.64	7.18e-6
rs7078511	10	104273864	C/A	0.32	SUFU	intron	1.61e-13	2.83	2.32e-4	2.95	3.58e-6	2.61	9.47e-6
rs4917968	10	104210566	G/A	0.30	C10orf95, TMEM180	intergenic	1.86e-13	-2.23	1.13e-3	-3.07	3.06e-6	-3.08	2.13e-6
rs5870	10	104229090	A/G	0.33	ACTR1A	UTR	2.45e-13	2.86	1.97e-4	2.75	1.60e-5	2.72	4.14e-6
rs1056744	10	104226677	A/G	0.33	TMEM180	UTR	2.67e-13	2.91	1.53e-4	2.75	1.50e-5	2.66	6.29e-6
rs11593971	10	104224080	G/C	0.33	TMEM180	intron	3.03e-13	2.91	1.53e-4	2.75	1.50e-5	2.64	7.17e-6
rs729023	10	104331877	A/G	0.34	SUFU	intron	3.11e-13	2.92	1.41e-4	2.86	7.77e-6	2.57	1.50e-5

SNP	Chr	Position	Alleles	MAF	Gene	Location	Meta P-value	Arizona		Oklahoma		North/South Dakotas	
								Beta	P-value	Beta	P-value	Beta	P-value
rs7906115	10	104317154	G/A	0.33	SUFU	intron	3.12e-13	2.92	1.38e-4	2.76	1.50e-5	2.65	8.23e-6
rs10786684	10	104313018	A/C	0.33	SUFU	intron	4.17e-13	2.89	1.64e-4	2.83	9.07e-6	2.57	1.50e-5
rs7342070	10	104213618	A/G	0.33	TMEM180	intron	4.44e-13	2.91	1.53e-4	2.72	1.60e-5	2.60	1.00e-5
rs3862030	10	104317574	G/A	0.33	SUFU	intron	5.62e-13	2.92	1.38e-4	2.76	1.50e-5	2.57	1.50e-5
rs181536287	10	104330968	A/G	0.34	SUFU	intron	5.62e-13	2.92	1.38e-4	2.76	1.50e-5	2.57	1.50e-5
rs729024	10	104331965	G/A	0.33	SUFU	intron	5.62e-13	2.92	1.38e-4	2.76	1.50e-5	2.57	1.50e-5
rs3936017	10	104344414	G/A	0.33	SUFU	intron	5.62e-13	2.92	1.38e-4	2.76	1.50e-5	2.57	1.50e-5
rs4919663	10	104353283	G/A	0.33	SUFU	intron	5.62e-13	2.92	1.38e-4	2.76	1.50e-5	2.57	1.50e-5
rs7075269	10	104355714	A/G	0.33	SUFU	intron	5.62e-13	2.92	1.38e-4	2.76	1.50e-5	2.57	1.50e-5
rs4919665	10	104356032	G/A	0.33	SUFU	intron	5.62e-13	2.92	1.38e-4	2.76	1.50e-5	2.57	1.50e-5
rs4917977	10	104353109	A/G	0.34	SUFU	intron	6.33e-13	2.92	1.38e-4	2.74	1.70e-5	2.57	1.50e-5
rs10883738	10	104311711	G/A	0.33	SUFU	intron	6.66e-13	2.89	1.64e-4	2.76	1.50e-5	2.57	1.50e-5
rs6584512	10	104319675	G/A	0.33	SUFU	intron	6.66e-13	2.89	1.64e-4	2.76	1.50e-5	2.57	1.50e-5
rs7091252	10	104318849	A/C	0.34	SUFU	intron	8.08e-13	2.92	1.38e-4	2.70	2.20e-5	2.57	1.50e-5
rs7907760	10	104335187	A/G	0.34	SUFU	intron	8.08e-13	2.92	1.38e-4	2.70	2.20e-5	2.57	1.50e-5
rs10748826	10	104344794	G/A	0.34	SUFU	intron	8.08e-13	2.92	1.38e-4	2.70	2.20e-5	2.57	1.50e-5
rs10786689	10	104346349	G/A	0.34	SUFU	intron	8.08e-13	2.92	1.38e-4	2.70	2.20e-5	2.57	1.50e-5
rs4919661	10	104348393	G/A	0.34	SUFU	intron	8.08e-13	2.92	1.38e-4	2.70	2.20e-5	2.57	1.50e-5
rs4917975	10	104348397	C/G	0.34	SUFU	intron	8.08e-13	2.92	1.38e-4	2.70	2.20e-5	2.57	1.50e-5

SNP	Chr	Position	Alleles	MAF	Gene	Location	Meta P-value	Arizona		Oklahoma		North/South Dakotas	
								Beta	P-value	Beta	P-value	Beta	P-value
rs4917976	10	104350508	A/G	0.34	SUFU	intron	8.08e-13	2.92	1.38e-4	2.70	2.20e-5	2.57	1.50e-5
rs10786691	10	104354642	A/G	0.34	SUFU	intron	8.08e-13	2.92	1.38e-4	2.70	2.20e-5	2.57	1.50e-5
rs11191352	10	104362234	G/A	0.34	SUFU	intron	8.08e-13	2.92	1.38e-4	2.70	2.20e-5	2.57	1.50e-5
rs10883753	10	104362431	A/G	0.34	SUFU	intron	8.08e-13	2.92	1.38e-4	2.70	2.20e-5	2.57	1.50e-5
rs10786682	10	104308312	A/C	0.33	SUFU	intron	9.55e-13	2.89	1.64e-4	2.70	2.20e-5	2.57	1.50e-5
rs10883736	10	104310019	A/C	0.33	SUFU	intron	9.55e-13	2.89	1.64e-4	2.70	2.20e-5	2.57	1.50e-5
rs10786683	10	104310466	A/G	0.33	SUFU	intron	9.55e-13	2.89	1.64e-4	2.70	2.20e-5	2.57	1.50e-5
rs10786685	10	104313093	A/G	0.33	SUFU	intron	9.55e-13	2.89	1.64e-4	2.70	2.20e-5	2.57	1.50e-5
rs4919664	10	104353398	C/A	0.33	SUFU	intron	9.55e-13	2.89	1.64e-4	2.70	2.20e-5	2.57	1.50e-5
rs10786695	10	104358746	C/A	0.33	SUFU	intron	9.55e-13	2.89	1.64e-4	2.70	2.20e-5	2.57	1.50e-5
rs11594073	10	104298183	A/G	0.33	SUFU	intron	1.19e-12	2.92	1.38e-4	2.75	1.60e-5	2.48	3.00e-5
rs11593710	10	104318709	A/C	0.33	SUFU	intron	1.29e-12	2.92	1.38e-4	2.65	3.60e-5	2.55	1.50e-5
rs12782153	10	104358284	A/G	0.33	SUFU	intron	2.01e-12	2.89	1.64e-4	2.77	1.40e-5	2.41	4.80e-5
rs11595170	10	104329841	A/G	0.33	SUFU	intron	2.36e-12	2.92	1.38e-4	2.72	2.00e-5	2.41	4.80e-5
rs12779854	10	104348464	G/A	0.33	SUFU	intron	2.36e-12	2.92	1.38e-4	2.72	2.00e-5	2.41	4.80e-5
rs11191393	10	104517420	G/C	0.27	C10orf26	intron	2.61e-12	-3.42	9.26e-7	-2.91	6.53e-6	-1.97	4.43e-3
rs72845847	10	104521596	A/G	0.27	C10orf26	intron	2.61e-12	-3.42	9.26e-7	-2.91	6.53e-6	-1.97	4.43e-3
rs1339919	10	104524438	A/G	0.27	C10orf26	intron	2.61e-12	-3.42	9.26e-7	-2.91	6.53e-6	-1.97	4.43e-3
rs7907417	10	104360652	A/G	0.33	SUFU	intron	2.78e-12	2.89	1.64e-4	2.72	2.00e-5	2.41	4.80e-5

SNP	Chr	Position	Alleles	MAF	Gene	Location	Meta P-value	Arizona		Oklahoma		North/South Dakotas	
								Beta	P-value	Beta	P-value	Beta	P-value
rs11191389	10	104513008	G/A	0.28	C10orf26	intron	4.40e-12	-3.42	9.26e-7	-2.83	1.20e-5	-1.98	4.37e-3
rs4147155	10	104526761	G/A	0.28	C10orf26	intron	4.40e-12	-3.42	9.26e-7	-2.83	1.20e-5	-1.98	4.37e-3
rs4147157	10	104526350	G/A	0.26	C10orf26	intron	5.17e-12	-3.46	1.03e-6	-2.84	1.70e-5	-2.08	3.68e-3
rs11191355	10	104382487	A/G	0.28	SUFU	UTR	6.20e-12	-3.27	3.79e-6	-2.88	7.26e-6	-1.97	3.53e-3
rs60459635	10	104490978	A/C	0.28	SFXN2, C10orf26	intergenic	6.77e-12	-3.43	8.70e-7	-2.87	8.08e-6	-1.84	7.87e-3
rs2297450	10	104493164	A/G	0.28	SFXN2, C10orf26	intergenic	6.77e-12	-3.43	8.70e-7	-2.87	8.08e-6	-1.84	7.87e-3
rs11191356	10	104383479	A/G	0.26	SUFU, TRIM8	intergenic	1.08e-11	-3.21	6.82e-6	-2.89	1.10e-5	-2.08	2.87e-3
rs17114534	10	104211519	A/G	0.28	TMEM180	intron	1.33e-11	-3.08	1.10e-5	-2.83	1.30e-5	-2.09	2.24e-3
rs11191359	10	104391476	T/A	0.26	SUFU, TRIM8	intergenic	1.49e-11	-3.19	7.69e-6	-2.89	1.30e-5	-2.07	3.06e-3
rs17114803	10	104376924	A/G	0.27	SUFU	coding	1.52e-11	-3.28	4.25e-6	-2.89	1.10e-5	-1.96	4.91e-3
rs12573474	10	104383496	A/G	0.26	SUFU, TRIM8	intergenic	1.63e-11	-3.17	8.88e-6	-2.79	2.30e-5	-2.16	2.01e-3
rs7086898	10	104376142	A/G	0.27	SUFU	intron	1.64e-11	-3.26	4.70e-6	-2.89	1.10e-5	-1.96	4.91e-3
rs10786700	10	104377725	G/A	0.27	SUFU	intron	1.64e-11	-3.26	4.70e-6	-2.89	1.10e-5	-1.96	4.91e-3
rs17114810	10	104381535	G/A	0.27	SUFU	UTR	1.64e-11	-3.26	4.70e-6	-2.89	1.10e-5	-1.96	4.91e-3
rs11818043	10	104381617	A/G	0.27	SUFU	UTR	1.64e-11	-3.26	4.70e-6	-2.89	1.10e-5	-1.96	4.91e-3
rs17114808	10	104381275	G/A	0.27	SUFU	UTR	1.64e-11	-3.26	4.70e-6	-2.89	1.10e-5	-1.96	4.91e-3

SNP	Chr	Position	Alleles	MAF	Gene	Location	Meta P-value	Arizona		Oklahoma		North/South Dakotas	
								Beta	P-value	Beta	P-value	Beta	P-value
rs4919666	10	104374019	G/A	0.27	SUFU	intron	1.64e-11	-3.26	4.70e-6	-2.89	1.10e-5	-1.96	4.91e-3
rs2902544	10	104251349	G/A	0.27	ACTR1A	intron	1.79e-11	-2.83	5.70e-5	-3.09	2.69e-6	-2.06	2.72e-3
rs4919649	10	104262326	A/G	0.27	SUFU	intron	1.90e-11	-2.81	6.30e-5	-3.11	2.61e-6	-2.06	2.72e-3
rs193185980	10	104258849	G/A	0.27	SUFU	intron	1.95e-11	-2.81	6.30e-5	-3.09	2.69e-6	-2.06	2.72e-3
rs4919656	10	104272851	G/A	0.27	SUFU	intron	1.95e-11	-2.81	6.30e-5	-3.09	2.69e-6	-2.06	2.72e-3
rs185072800	10	104258850	C/A	0.27	SUFU	intron	1.95e-11	-2.81	6.30e-5	-3.09	2.69e-6	-2.07	2.72e-3
rs74558061	10	104374327	A/G	0.26	SUFU	intron	2.10e-11	-3.26	4.81e-6	-2.89	1.30e-5	-1.95	5.23e-3
rs4919643	10	104212367	G/C	0.28	TMEM180	intron	2.17e-11	-2.98	2.30e-5	-2.88	1.20e-5	-2.10	2.16e-3
rs12415043	10	104457599	C/G	0.26	ARL3	intron	2.17e-11	-3.20	6.06e-6	-2.98	6.14e-6	-1.87	7.48e-3
rs7089422	10	104595318	G/A	0.14	CYP17A1, C10orf32	intergenic	2.18e-11	-4.12	3.34e-3	-2.78	2.77e-4	-3.61	8.86e-7
rs4146429	10	104391622	A/G	0.26	SUFU, TRIM8	intergenic	2.44e-11	-3.18	7.95e-6	-2.79	2.20e-5	-2.07	3.06e-3
rs4146428	10	104391711	G/A	0.26	SUFU, TRIM8	intergenic	2.44e-11	-3.18	7.95e-6	-2.79	2.20e-5	-2.07	3.06e-3
rs10786701	10	104402298	G/A	0.26	TRIM8	intron	2.44e-11	-3.18	7.95e-6	-2.79	2.20e-5	-2.07	3.06e-3
rs10883761	10	104410008	A/G	0.26	TRIM8, ARL3	intergenic	2.44e-11	-3.18	7.95e-6	-2.79	2.20e-5	-2.07	3.06e-3
rs10883766	10	104454753	G/A	0.26	ARL3	intron	2.67e-11	-3.20	6.06e-6	-2.96	7.88e-6	-1.87	7.48e-3
rs4919669	10	104461965	T/A	0.26	ARL3	intron	3.04e-11	-3.20	6.06e-6	-2.94	9.18e-6	-1.87	7.48e-3

SNP	Chr	Position	Alleles	MAF	Gene	Location	Meta P-value	Arizona		Oklahoma		North/South Dakotas	
								Beta	P-value	Beta	P-value	Beta	P-value
rs4244354	10	104416167	G/A	0.26	TRIM8, ARL3	intergenic	3.16e-11	-3.18	7.95e-6	-2.79	2.20e-5	-2.03	3.74e-3
rs11191365	10	104420241	G/A	0.26	TRIM8, ARL3	intergenic	3.16e-11	-3.18	7.95e-6	-2.79	2.20e-5	-2.03	3.74e-3
rs8354	10	104426631	G/A	0.26	ARL3	UTR	3.94e-11	-3.09	1.20e-5	-2.98	6.61e-6	-1.87	7.48e-3
rs2298278	10	104380293	A/G	0.27	SUFU	UTR	6.12e-11	-3.13	1.10e-5	-2.77	2.50e-5	-1.97	4.69e-3
rs5011218	10	104391208	A/C	0.26	SUFU, TRIM8	intergenic	7.69e-11	-3.08	1.60e-5	-2.69	4.30e-5	-2.07	3.06e-3
rs140683362	10	104676874	C/G	0.16	CNNM2	intron	7.88e-11	-4.02	4.02e-3	-2.59	5.80e-4	-3.48	1.30e-6
rs729025	10	104332196	G/A	0.27	SUFU	intron	7.96e-11	-2.86	5.10e-5	-2.96	7.39e-6	-1.96	4.91e-3
rs3824756	10	104349340	A/G	0.27	SUFU	intron	7.96e-11	-2.86	5.10e-5	-2.96	7.39e-6	-1.96	4.91e-3
rs17114641	10	104277349	A/C	0.27	SUFU	intron	8.20e-11	-2.90	3.90e-5	-2.94	9.06e-6	-1.95	5.23e-3
rs10883735	10	104298436	G/A	0.27	SUFU	intron	8.89e-11	-2.90	3.90e-5	-2.93	1.00e-5	-1.95	5.23e-3
rs2902548	10	104477372	G/A	0.27	SFXN2	intron	9.95e-11	-3.58	2.88e-7	-2.69	2.90e-5	-1.45	3.64e-2
rs2778038	10	104505059	A/C	0.11	C10orf26	intron	1.02e-10	-4.87	4.40e-4	-2.78	3.69e-3	-3.98	2.31e-6
rs3934495	10	104351701	G/A	0.27	SUFU	intron	1.06e-10	-2.86	5.30e-5	-2.94	9.06e-6	-1.95	5.23e-3
rs10883737	10	104310888	G/C	0.27	SUFU	intron	1.12e-10	-2.84	5.80e-5	-2.96	8.87e-6	-1.95	5.23e-3
rs2432580	10	104552202	G/A	0.33	C10orf26	intron	1.14e-10	2.12	1.47e-3	2.41	1.01e-4	2.76	5.00e-5
rs11191343	10	104335215	G/A	0.27	SUFU	intron	1.14e-10	-2.84	5.80e-5	-2.94	9.06e-6	-1.95	5.23e-3
rs2244254	10	104479001	G/C	0.12	SFXN2	intron	1.16e-10	-5.13	1.92e-4	-2.74	3.29e-3	-3.72	7.83e-6

SNP	Chr	Position	Alleles	MAF	Gene	Location	Meta P-value	Arizona		Oklahoma		North/South Dakotas	
								Beta	P-value	Beta	P-value	Beta	P-value
rs284859	10	104563007	C/A	0.33	C10orf26	coding	1.19e-10	2.15	1.31e-3	2.30	1.91e-4	2.85	3.10e-5
rs3740409	10	104226635	G/A	0.27	TMEM180	UTR	1.21e-10	-2.66	1.69e-4	-2.93	1.10e-5	-2.10	2.32e-3
rs186550244	10	104329779	C/A	0.27	SUFU	intron	1.28e-10	-2.85	5.60e-5	-2.96	8.87e-6	-1.92	5.93e-3
rs4919644	10	104219578	G/A	0.27	TMEM180	intron	1.29e-10	-2.66	1.81e-4	-2.93	1.10e-5	-2.10	2.32e-3
rs7092340	10	104519658	A/G	0.12	C10orf26	intron	1.58e-10	-5.07	2.38e-4	-2.74	3.63e-3	-3.72	7.83e-6
rs12266856	10	104521040	G/A	0.12	C10orf26	intron	1.62e-10	-5.03	2.44e-4	-2.74	3.63e-3	-3.72	7.83e-6
rs2031604	10	104287781	G/A	0.27	SUFU	intron	1.89e-10	-2.90	3.90e-5	-2.93	8.51e-6	-1.78	1.01e-2
rs2244258	10	104478890	T/A	0.12	SFXN2	intron	1.98e-10	-5.13	1.92e-4	-2.75	3.37e-3	-3.63	1.40e-5
rs145033011	10	104405808	A/G	0.04	TRIM8	intron	2.39e-10	6.21	2.34e-3	6.40	5.36e-4	6.21	1.10e-5
rs284849	10	104581172	C/A	0.33	CYP17A1	intron	2.86e-10	2.50	2.34e-4	2.31	2.45e-4	2.41	3.23e-4
rs284848	10	104582115	G/A	0.33	CYP17A1	intron	2.86e-10	2.50	2.34e-4	2.31	2.45e-4	2.41	3.23e-4
rs2486758	10	104587470	A/G	0.33	CYP17A1, C10orf32	intergenic	5.08e-10	2.49	2.28e-4	2.29	2.72e-4	2.29	5.23e-4
rs2265308	10	104481104	G/A	0.12	SFXN2	intron	1.47e-9	-5.07	2.38e-4	-2.51	6.30e-3	-3.31	4.80e-5
rs2778037	10	104482697	G/C	0.12	SFXN2	intron	1.47e-9	-5.07	2.38e-4	-2.51	6.30e-3	-3.31	4.80e-5
rs9420873	10	104486012	G/A	0.12	SFXN2	intron	1.47e-9	-5.07	2.38e-4	-2.51	6.30e-3	-3.31	4.80e-5
rs7917772	10	104477433	G/A	0.38	SFXN2	intron	1.59e-9	2.45	7.71e-4	2.13	4.19e-4	2.12	3.66e-4
rs58706228	10	104499808	A/G	0.12	C10orf26	intron	1.95e-9	-5.07	2.38e-4	-2.45	7.96e-3	-3.31	4.80e-5
rs2244524	10	104476964	A/G	0.38	SFXN2	intron	2.83e-9	2.50	5.76e-4	2.42	6.90e-5	1.73	3.62e-3

SNP	Chr	Position	Alleles	MAF	Gene	Location	Meta P-value	Arizona		Oklahoma		North/South Dakotas	
								Beta	P-value	Beta	P-value	Beta	P-value
rs61869257	10	104506262	G/A	0.11	C10orf26	intron	3.40e-9	-4.70	1.00e-3	-2.63	5.38e-3	-3.51	3.60e-5
rs7090307	10	104507109	G/A	0.11	C10orf26	intron	3.40e-9	-4.70	1.00e-3	-2.63	5.38e-3	-3.51	3.60e-5
rs7077678	10	104428555	A/G	0.40	ARL3	intron	3.86e-9	2.39	8.92e-4	2.20	2.16e-4	1.90	1.38e-3
rs2286748	10	104404699	C/A	0.14	TRIM8	intron	3.89e-9	-5.65	7.97e-6	-1.50	7.29e-2	-3.20	6.30e-5
rs11191369	10	104447446	A/G	0.40	ARL3	intron	3.97e-9	2.39	8.92e-4	2.21	2.23e-4	1.90	1.38e-3
rs11191377	10	104462767	A/G	0.13	ARL3	intron	3.98e-9	-4.51	8.17e-4	-2.26	8.86e-3	-3.40	2.70e-5
rs10786705	10	104467695	G/A	0.13	SFXN2	intron	3.98e-9	-4.51	8.17e-4	-2.26	8.86e-3	-3.40	2.70e-5
rs10883776	10	104531009	A/G	0.11	C10orf26	intron	4.72e-9	-4.70	1.00e-3	-2.57	7.15e-3	-3.51	3.60e-5
rs7893687	10	104447593	A/T	0.13	ARL3	intron	4.97e-9	-4.51	8.17e-4	-2.20	1.07e-2	-3.40	2.70e-5
rs28408682	10	104393300	A/G	0.40	SUFU, TRIM8	intergenic	6.45e-9	2.33	1.45e-3	2.38	9.00e-5	1.74	2.94e-3
rs6892	10	104565860	A/G	0.25	C10orf26	UTR	7.43e-9	2.71	4.61e-3	2.58	1.26e-3	3.31	9.60e-5
rs11191362	10	104409225	G/A	0.13	TRIM8, ARL3	intergenic	1.22e-8	-4.87	2.15e-4	-1.45	9.02e-2	-3.61	8.42e-6
rs10883762	10	104410506	A/G	0.13	TRIM8, ARL3	intergenic	1.22e-8	-4.87	2.15e-4	-1.45	9.02e-2	-3.61	8.42e-6
rs10883756	10	104390102	A/G	0.40	SUFU, TRIM8	intergenic	1.24e-8	2.21	2.42e-3	2.33	1.11e-4	1.74	2.94e-3
rs3977755	10	104410200	G/A	0.13	TRIM8, ARL3	intergenic	1.32e-8	-4.87	2.15e-4	-1.43	9.44e-2	-3.61	8.42e-6
rs2244647	10	104476294	G/C	0.12	SFXN2	intron	2.00e-8	-4.74	8.13e-4	-1.92	3.69e-2	-3.56	2.10e-5

SNP	Chr	Position	Alleles	MAF	Gene	Location	Meta P-value	Arizona		Oklahoma		North/South Dakotas	
								Beta	P-value	Beta	P-value	Beta	P-value
rs2790914	10	104558008	G/A	0.36	C10orf26	intron	2.31e-8	2.05	2.02e-3	1.95	1.19e-3	2.19	8.24e-4
rs10883759	10	104402039	G/A	0.14	TRIM8	intron	2.64e-8	-5.65	7.97e-6	-1.34	1.04e-1	-2.85	2.84e-4
rs2250301	10	104538383	G/A	0.35	C10orf26	intron	2.69e-8	2.05	2.02e-3	2.00	9.10e-4	2.17	1.24e-3
rs3802676	10	104481692	G/A	0.11	SFXN2	intron	2.81e-8	-4.70	1.00e-3	-2.42	9.34e-3	-3.10	1.99e-4
rs10883769	10	104484937	G/C	0.11	SFXN2	intron	2.81e-8	-4.70	1.00e-3	-2.42	9.34e-3	-3.10	1.99e-4
rs7912571	10	104489378	A/G	0.11	SFXN2, C10orf26	intergenic	2.81e-8	-4.70	1.00e-3	-2.42	9.34e-3	-3.10	1.99e-4
rs7092723	10	104489754	G/A	0.11	SFXN2, C10orf26	intergenic	2.81e-8	-4.70	1.00e-3	-2.42	9.34e-3	-3.10	1.99e-4
rs7074894	10	104494524	A/C	0.11	C10orf26	intron	2.81e-8	-4.70	1.00e-3	-2.42	9.34e-3	-3.10	1.99e-4
rs7923711	10	104496533	G/C	0.11	C10orf26	intron	3.05e-8	-4.70	1.00e-3	-2.40	1.00e-2	-3.10	1.99e-4
rs7342026	10	104498896	A/G	0.11	C10orf26	intron	3.05e-8	-4.70	1.00e-3	-2.40	1.00e-2	-3.10	1.99e-4
rs10883771	10	104500148	A/G	0.11	C10orf26	intron	3.13e-8	-4.68	1.04e-3	-2.40	1.00e-2	-3.10	1.97e-4
rs3781571	10	104432447	G/A	0.14	ARL3	intron	3.25e-8	-4.51	8.17e-4	-2.01	1.70e-2	-3.03	1.27e-4
rs10883770	10	104496842	G/A	0.11	C10orf26	intron	3.56e-8	-3.86	5.63e-3	-2.44	9.91e-3	-3.51	3.60e-5
rs11191384	10	104496101	A/G	0.11	C10orf26	intron	3.60e-8	-4.70	1.00e-3	-2.34	1.16e-2	-3.10	1.99e-4
rs7089279	10	104459417	G/A	0.14	ARL3	intron	3.68e-8	-4.51	8.17e-4	-2.02	1.77e-2	-3.03	1.39e-4
rs3740606	10	104435358	G/A	0.14	ARL3	intron	3.95e-8	-4.51	8.17e-4	-1.96	2.00e-2	-3.03	1.27e-4
rs284846	10	104547280	A/G	0.35	C10orf26	intron	4.09e-8	2.05	2.02e-3	1.99	9.85e-4	2.10	1.75e-3

SNP	Chr	Position	Alleles	MAF	Gene	Location	Meta P-value	Arizona		Oklahoma		North/South Dakotas	
								Beta	P-value	Beta	P-value	Beta	P-value
rs2778039	10	104550273	A/G	0.35	C10orf26	intron	4.21e-8	2.05	2.02e-3	1.99	1.02e-3	2.10	1.75e-3
rs7893954	10	104308956	G/A	0.39	SUFU	intron	4.69e-8	2.63	4.65e-4	1.38	2.17e-2	2.19	2.37e-4
rs7908450	10	104749181	C/G	0.10	CNNM2	intron	7.76e-8	-5.23	2.08e-3	-1.81	6.50e-2	-3.71	1.50e-5
rs3977747	10	104411558	A/C	0.14	TRIM8, ARL3	intergenic	8.38e-8	-4.87	2.15e-4	-1.28	0.12	-3.24	4.80e-5
rs7899004	10	104331425	G/A	0.39	SUFU	intron	8.79e-8	2.15	4.07e-3	2.38	8.80e-5	1.47	1.24e-2
rs58109969	10	104592597	G/C	0.05	CYP17A1, C10orf32	intergenic	1.65e-7	-4.76	9.56e-3	-2.93	3.79e-2	-4.91	1.60e-5
rs10509762	10	104596462	A/C	0.05	CYP17A1, C10orf32	intergenic	1.65e-7	-4.76	9.56e-3	-2.93	3.79e-2	-4.91	1.60e-5
rs17115171	10	104614662	A/G	0.05	C10orf32, AS3MT	intergenic	1.83e-7	-4.76	1.04e-2	-2.93	3.87e-2	-4.91	1.60e-5
rs17115203	10	104629959	A/G	0.05	AS3MT	intron	2.08e-7	-4.56	1.21e-2	-2.93	3.79e-2	-4.91	1.60e-5

Abbreviations: Chr, chromosome. MAF, minor allele frequency. SNP, single nucleotide polymorphism.

All SNP 463 associations are below MetaboChip-wide significance threshold of 3.57e-7 for meta-analysis. Base position according to human genome build 18.

Table S7. Top MetaboChip associations for principal component 2 of arsenic species

SNP	Chr	Position	Alleles	MAF	Gene	Location	Meta P-value	Arizona		Oklahoma		North/South Dakotas	
								Beta	P-value	Beta	P-value	Beta	P-value
rs7098825	10	104618224	A/G	0.17	C10orf32, AS3MT	intergenic	2.17e-9	1.39	2.91e-4	1.14	8.62e-4	1.24	5.88e-4
rs3740393	10	104626645	G/C	0.21	AS3MT	intron	1.56e-8	1.19	2.06e-4	0.90	1.39e-3	0.90	3.20e-3
rs79668541	10	104783894	G/A	0.19	CNNM2	intron	1.99e-8	1.16	4.11e-4	0.95	9.69e-4	0.96	3.29e-3
rs17878846	10	104620402	T/A	0.19	AS3MT	intron	2.21e-8	1.12	5.37e-4	0.97	8.25e-4	0.96	3.39e-3
rs3824754	10	104604340	G/A	0.19	C10orf32	intron	2.77e-8	1.10	7.04e-4	0.97	8.25e-4	0.96	3.39e-3
rs11191416	10	104594906	A/C	0.19	CYP17A1, C10orf32	intergenic	2.82e-8	1.10	7.04e-4	0.96	8.39e-4	0.96	3.39e-3
rs11191425	10	104615960	G/A	0.19	C10orf32, AS3MT	intergenic	2.82e-8	1.10	7.04e-4	0.96	8.39e-4	0.96	3.39e-3
rs184992072	10	104846756	A/G	0.19	NT5C2	intron	2.95e-8	1.06	1.24e-3	0.93	1.20e-3	1.03	1.62e-3
rs3824755	10	104585839	C/G	0.19	CYP17A1	intron	3.02e-8	1.06	1.12e-3	0.96	8.39e-4	0.96	2.51e-3
rs1004467	10	104584497	A/G	0.19	CYP17A1	intron	3.02e-8	1.06	1.12e-3	0.96	8.39e-4	0.96	2.51e-3
rs2297787	10	104670127	T/A	0.20	CNNM2	intron	3.44e-8	1.03	1.60e-3	0.96	7.54e-4	0.98	2.30e-3
rs3740390	10	104628470	G/A	0.19	AS3MT	intron	3.47e-8	1.08	8.39e-4	0.96	9.30e-4	0.96	3.29e-3
rs113282265	10	104647238	A/G	0.19	AS3MT	intron	3.47e-8	1.08	8.39e-4	0.96	9.30e-4	0.96	3.29e-3
rs4409766	10	104606653	A/G	0.19	C10orf32	intron	4.22e-8	1.06	1.12e-3	0.93	1.20e-3	0.96	2.51e-3
rs11191479	10	104713610	A/G	0.19	CNNM2	intron	4.22e-8	1.05	1.32e-3	0.96	9.30e-4	0.98	2.72e-3

SNP	Chr	Position	Alleles	MAF	Gene	Location	Meta P-value	Arizona		Oklahoma		North/South Dakotas	
								Beta	P-value	Beta	P-value	Beta	P-value
rs12219901	10	104830957	A/G	0.19	CNNM2, NT5C2	intergenic	4.35e-8	1.05	1.32e-3	0.97	7.74e-4	0.96	3.29e-3
rs78821730	10	104674534	G/A	0.19	CNNM2	intron	4.98e-8	1.05	1.32e-3	0.96	8.97e-4	0.96	3.29e-3
rs11191595	10	104933038	A/C	0.19	NT5C2	intron	5.08e-8	1.11	6.76e-4	0.91	1.64e-3	0.95	3.38e-3
rs11191453	10	104649842	A/G	0.19	AS3MT	intron	5.14e-8	1.05	1.32e-3	0.96	9.30e-4	0.96	3.29e-3
rs12221193	10	104655257	A/C	0.19	AS3MT, CNNM2	intergenic	5.14e-8	1.05	1.32e-3	0.96	9.30e-4	0.96	3.29e-3
rs77180047	10	104656747	G/A	0.19	AS3MT, CNNM2	intergenic	5.14e-8	1.05	1.32e-3	0.96	9.30e-4	0.96	3.29e-3
rs17115213	10	104671133	A/G	0.19	CNNM2	intron	5.14e-8	1.05	1.32e-3	0.96	9.30e-4	0.96	3.29e-3
rs10509759	10	104679655	T/A	0.19	CNNM2	intron	5.14e-8	1.05	1.32e-3	0.96	9.30e-4	0.96	3.29e-3
rs5011520	10	104687506	G/A	0.19	CNNM2	intron	5.14e-8	1.05	1.32e-3	0.96	9.30e-4	0.96	3.29e-3
rs10883808	10	104711116	T/A	0.19	CNNM2	intron	5.14e-8	1.05	1.32e-3	0.96	9.30e-4	0.96	3.29e-3
rs10883815	10	104729169	A/G	0.19	CNNM2	intron	5.14e-8	1.05	1.32e-3	0.96	9.30e-4	0.96	3.29e-3
rs11191502	10	104755484	A/C	0.19	CNNM2	intron	5.14e-8	1.05	1.32e-3	0.96	9.30e-4	0.96	3.29e-3
rs11191514	10	104763354	G/A	0.19	CNNM2	intron	5.14e-8	1.05	1.32e-3	0.96	9.30e-4	0.96	3.29e-3
rs77787671	10	104766195	G/A	0.19	CNNM2	intron	5.14e-8	1.05	1.32e-3	0.96	9.30e-4	0.96	3.29e-3
rs75970938	10	104783638	A/G	0.19	CNNM2	intron	5.14e-8	1.05	1.32e-3	0.96	9.30e-4	0.96	3.29e-3
rs11191535	10	104805866	G/A	0.19	CNNM2	intron	5.14e-8	1.05	1.32e-3	0.96	9.30e-4	0.96	3.29e-3
rs10458729	10	104831469	G/A	0.19	CNNM2,	intergenic	5.14e-8	1.05	1.32e-3	0.96	9.30e-4	0.96	3.29e-3

SNP	Chr	Position	Alleles	MAF	Gene	Location	Meta P-value	Arizona		Oklahoma		North/South Dakotas	
								Beta	P-value	Beta	P-value	Beta	P-value
					NT5C2								
rs11191454	10	104649994	A/G	0.19	AS3MT	intron	5.14e-8	1.05	1.32e-3	0.96	9.30e-4	0.96	3.29e-3
rs11191472	10	104697006	T/A	0.19	CNNM2	intron	5.14e-8	1.05	1.32e-3	0.96	9.30e-4	0.96	3.29e-3
rs11191515	10	104766517	G/A	0.19	CNNM2	intron	5.14e-8	1.05	1.32e-3	0.96	9.30e-4	0.96	3.29e-3
rs11191548	10	104836168	A/G	0.19	CNNM2, NT5C2	intergenic	5.14e-8	1.05	1.32e-3	0.96	9.30e-4	0.96	3.29e-3
rs12411886	10	104675289	C/A	0.19	CNNM2	intron	5.14e-8	1.05	1.32e-3	0.96	9.30e-4	0.96	3.29e-3
rs12413409	10	104709086	G/A	0.19	CNNM2	intron	5.14e-8	1.05	1.32e-3	0.96	9.30e-4	0.96	3.29e-3
rs3781285	10	104815655	C/G	0.19	CNNM2	intron	5.14e-8	1.05	1.32e-3	0.96	9.30e-4	0.96	3.29e-3
rs12221064	10	104667116	G/A	0.19	AS3MT, CNNM2	intergenic	5.37e-8	1.05	1.32e-3	0.95	9.75e-4	0.96	3.29e-3
rs12217501	10	104841879	A/G	0.19	NT5C2	intron	5.46e-8	1.06	1.27e-3	0.95	1.03e-3	0.96	3.29e-3
rs12220743	10	104841902	G/A	0.19	NT5C2	intron	5.63e-8	1.05	1.32e-3	0.95	1.03e-3	0.96	3.29e-3
rs943037	10	104825909	G/A	0.19	CNNM2	coding	5.63e-8	1.05	1.32e-3	0.95	1.03e-3	0.96	3.29e-3
rs11191555	10	104847513	A/C	0.19	NT5C2	intron	5.81e-8	1.05	1.32e-3	0.93	1.20e-3	0.96	2.95e-3
rs732998	10	104887891	A/G	0.19	NT5C2	intron	6.47e-8	1.05	1.31e-3	0.93	1.20e-3	0.96	3.29e-3
rs79237883	10	104930936	A/G	0.19	LOC729081, NT5C2	intergenic	6.51e-8	1.05	1.32e-3	0.93	1.20e-3	0.96	3.29e-3
rs11191558	10	104854668	G/A	0.19	NT5C2	intron	6.51e-8	1.05	1.32e-3	0.93	1.20e-3	0.96	3.29e-3
rs11191560	10	104859028	A/G	0.19	NT5C2	intron	6.51e-8	1.05	1.32e-3	0.93	1.20e-3	0.96	3.29e-3

SNP	Chr	Position	Alleles	MAF	Gene	Location	Meta P-value	Arizona		Oklahoma		North/South Dakotas	
								Beta	P-value	Beta	P-value	Beta	P-value
rs11191580	10	104896201	A/G	0.19	NT5C2	intron	6.51e-8	1.05	1.32e-3	0.93	1.20e-3	0.96	3.29e-3
rs12220375	10	104891481	A/G	0.19	NT5C2	intron	6.51e-8	1.05	1.32e-3	0.93	1.20e-3	0.96	3.29e-3
rs12413046	10	104861194	A/G	0.19	NT5C2	intron	6.51e-8	1.05	1.32e-3	0.93	1.20e-3	0.96	3.29e-3
rs9633712	10	104863751	G/C	0.19	NT5C2	intron	6.51e-8	1.05	1.32e-3	0.93	1.20e-3	0.96	3.29e-3
rs12412038	10	104846152	G/A	0.19	NT5C2	intron	6.73e-8	1.05	1.32e-3	0.93	1.20e-3	0.95	3.39e-3
rs10883832	10	104861269	A/C	0.19	NT5C2	intron	6.85e-8	1.05	1.32e-3	0.93	1.27e-3	0.96	3.29e-3
rs17884001	10	104651235	G/A	0.19	AS3MT	UTR	7.14e-8	1.05	1.32e-3	0.92	1.33e-3	0.96	3.29e-3
rs10430665	10	104838420	G/A	0.19	NT5C2	UTR	8.41e-8	1.03	1.76e-3	0.94	1.16e-3	0.95	3.43e-3
rs188299216	10	104944768	G/A	0.19	NT5C2, LOC401648	intergenic	8.71e-8	1.06	1.22e-3	0.94	1.09e-3	0.91	4.98e-3
rs1060240	10	104873327	A/G	0.19	NT5C2	intron	8.77e-8	1.06	1.31e-3	0.91	1.59e-3	0.95	3.45e-3
rs79780963	10	104942489	G/A	0.19	NT5C2	intron	9.75e-8	1.11	6.81e-4	0.91	1.60e-3	0.89	6.24e-3
rs77420391	10	104935813	G/A	0.19	NT5C2	intron	1.26e-7	1.05	1.32e-3	0.93	1.20e-3	0.90	6.08e-3
rs11191582	10	104903643	G/A	0.19	NT5C2	intron	1.26e-7	1.05	1.32e-3	0.93	1.20e-3	0.90	6.08e-3
rs11191447	10	104642313	G/A	0.19	AS3MT	intron	1.44e-7	1.08	8.39e-4	0.82	4.11e-3	0.96	3.29e-3
rs1926032	10	104819459	G/A	0.18	CNNM2	intron	1.65e-7	1.01	2.32e-3	0.86	3.58e-3	1.05	1.82e-3
rs12768205	10	104637839	G/A	0.27	AS3MT	intron	1.78e-7	1.04	6.56e-4	0.72	4.87e-3	0.80	4.13e-3
rs10883796	10	104645305	G/A	0.27	AS3MT	intron	1.78e-7	1.04	6.56e-4	0.72	4.87e-3	0.80	4.13e-3
rs17727044	10	104811246	T/A	0.17	CNNM2	intron	1.81e-7	-0.48	0.17	-1.35	5.10e-5	-1.00	4.82e-4

SNP	Chr	Position	Alleles	MAF	Gene	Location	Meta P-value	Arizona		Oklahoma		North/South Dakotas	
								Beta	P-value	Beta	P-value	Beta	P-value
rs78893207	10	104786128	A/G	0.17	CNNM2	intron	2.66e-7	-0.55	0.11	-1.21	2.20e-4	-1.01	4.28e-4
rs10786722	10	104650058	G/A	0.27	AS3MT	intron	2.68e-7	1.00	1.09e-3	0.72	4.87e-3	0.80	4.13e-3
rs7897654	10	104652448	A/G	0.27	AS3MT, CNNM2	intergenic	2.68e-7	1.00	1.09e-3	0.72	4.87e-3	0.80	4.13e-3
rs10883799	10	104653300	G/A	0.27	AS3MT, CNNM2	intergenic	2.68e-7	1.00	1.09e-3	0.72	4.87e-3	0.80	4.13e-3
rs1046778	10	104651474	A/G	0.27	AS3MT	UTR	2.68e-7	1.00	1.09e-3	0.72	4.87e-3	0.80	4.13e-3
rs12252500	10	104784974	G/C	0.17	CNNM2	intron	3.16e-7	-0.55	0.11	-1.21	2.74e-4	-1.01	4.28e-4
rs12257935	10	104793052	C/A	0.17	CNNM2	intron	3.16e-7	-0.55	0.11	-1.21	2.74e-4	-1.01	4.28e-4
rs12266291	10	104796874	G/A	0.17	CNNM2	intron	3.16e-7	-0.55	0.11	-1.21	2.74e-4	-1.01	4.28e-4
rs74749600	10	104722986	A/G	0.18	CNNM2	intron	3.20e-7	-0.39	0.24	-1.35	5.10e-5	-1.00	4.82e-4
rs11191523	10	104782390	A/G	0.18	CNNM2	intron	3.40e-7	-0.55	0.11	-1.19	3.00e-4	-1.01	4.28e-4
rs11191525	10	104783425	C/G	0.18	CNNM2	intron	3.40e-7	-0.55	0.11	-1.19	3.00e-4	-1.01	4.28e-4
rs58317752	10	104789257	A/T	0.18	CNNM2	intron	3.40e-7	-0.55	0.11	-1.19	3.00e-4	-1.01	4.28e-4
rs77827514	10	104811999	G/A	0.18	CNNM2	intron	3.40e-7	-0.55	0.11	-1.19	3.00e-4	-1.01	4.28e-4
rs78214351	10	104812678	A/G	0.18	CNNM2	intron	3.40e-7	-0.55	0.11	-1.19	3.00e-4	-1.01	4.28e-4
rs12257941	10	104823151	G/A	0.18	CNNM2	intron	3.40e-7	-0.55	0.11	-1.19	3.00e-4	-1.01	4.28e-4
rs12765002	10	104625338	G/A	0.25	AS3MT	intron	3.47e-7	0.99	1.56e-3	0.76	3.50e-3	0.79	5.44e-3
rs76892505	10	104709280	G/A	0.18	CNNM2	intron	3.61e-7	-0.37	0.27	-1.36	4.70e-5	-1.00	4.82e-4

SNP	Chr	Position	Alleles	MAF	Gene	Location	Meta P-value	Arizona		Oklahoma		North/South Dakotas	
								Beta	P-value	Beta	P-value	Beta	P-value
rs79331374	10	104872903	G/A	0.17	NT5C2	intron	3.71e-7	-0.55	0.11	-1.18	3.35e-4	-1.01	4.28e-4

Abbreviations: Chr, chromosome. MAF, minor allele frequency. SNP, single nucleotide polymorphism.

All SNP 84 associations are below MetaboChip-wide significance threshold of 3.57e-7 for meta-analysis. Base position according to human genome build 18.

Table S8. Top candidate SNP associations for percent inorganic arsenic

SNP	Chr	Position	Alleles	MAF	Gene	Location	Meta P-value	Arizona		Oklahoma		North/South Dakotas	
								Beta	P-value	Beta	P-value	Beta	P-value
rs4919694	10	104698978	T/C	0.19	CNNM2	intron	8.10e-10	0.18	6.50e-05	0.08	1.40e-05	0.12	0.02
rs3740390	10	104638480	C/T	0.2	AS3MT	intron	1.76e-08	-0.27	4.21e-10	-0.02	0.33	-0.15	5.26e-03
rs11191453	10	104659852	T/C	0.2	AS3MT	intron	6.48e-07	-0.26	6.71e-09	-0.03	0.79	-0.15	5.26e-03
rs7911488	10	105154089	A/G	0.26	USMG5	UTR	1.04e-06	-0.26	7.72e-09	-0.01	0.71	-0.12	0.01
rs3740394	10	104634474	A/G	0.19	AS3MT	intron	2.53e-06	0.19	4.10e-05	0.11	0.08	0.12	0.01

Abbreviations: Chr, chromosome. MAF, minor allele frequency. SNP, single nucleotide polymorphism.

All SNP 5 associations are below candidate SNP significance threshold of 9.33e-5 for meta-analysis. Base position according to human genome build 18.

Table S9. Top candidate SNP associations for percent monomethylarsonate

SNP	Chr	Position	Alleles	MAF	Gene	Location	Meta P-value	Arizona		Oklahoma		North/South Dakotas	
								Beta	P-value	Beta	P-value	Beta	P-value
rs3740390	10	104638480	C/T	0.20	AS3MT	intron	9.24e-13	-0.27	2.07e-14	-0.08	0.11	-0.14	5.97e-4
rs11191453	10	104659852	T/C	0.20	AS3MT	intron	5.57e-12	-0.26	6.69e-13	-0.07	0.11	-0.14	5.97e-4
rs7911488	10	105154089	A/G	0.26	USMG5	UTR	6.54e-8	-0.22	4.15e-10	-0.04	0.41	-0.09	9.93e-3
rs11191439	10	104638723	T/C	0.19	AS3MT	coding	2.60e-7	0.12	1.15e-3	0.10	0.06	0.14	1.51e-4
rs4919694	10	104698978	T/C	0.19	CNNM2	intron	4.74e-7	0.12	9.85e-4	0.09	0.10	0.14	1.66e-4

Abbreviations: Chr, chromosome. MAF, minor allele frequency. SNP, single nucleotide polymorphism.

All SNP 5 associations are below candidate SNP significance threshold of 9.33e-5 for meta-analysis. Base position according to human genome build 18.

Table S10. Top candidate SNP associations for percent dimethylarsinate

SNP	Chr	Position	Alleles	MAF	Gene	Location	Meta P-value	Arizona		Oklahoma		North/South Dakotas	
								Beta	P-value	Beta	P-value	Beta	P-value
rs3740390	10	104638480	C/T	0.20	AS3MT	intron	8.63e-23	0.30	2.69e-18	0.12	9.70e-4	0.19	1.40e-7
rs11191453	10	104659852	T/C	0.20	AS3MT	intron	2.18e-21	0.28	4.49e-16	0.12	1.02e-3	0.19	1.40e-7
rs3740394	10	104634474	A/G	0.19	AS3MT	intron	8.30e-20	-0.20	1.04e-8	-0.19	9.40e-6	-0.18	1.52e-8
rs11191439	10	104638723	T/C	0.19	AS3MT	coding	2.89e-19	-0.21	8.20e-9	-0.18	3.50e-5	-0.19	1.36e-8
rs4919694	10	104698978	T/C	0.19	CNNM2	intron	1.67e-18	-0.21	7.65e-9	-0.16	1.28e-4	-0.18	1.70e-8
rs7911488	10	105154089	A/G	0.26	USMG5	UTR	7.04e-18	0.26	4.80e-14	0.10	3.80e-3	0.15	2.29e-6

Abbreviations: Chr, chromosome. MAF, minor allele frequency. SNP, single nucleotide polymorphism.

All SNP 6 associations are below candidate SNP significance threshold of 9.33e-5 for meta-analysis. Base position according to human genome build 18.

Table S11. Top candidate SNP associations for principal component 1 of arsenic species

SNP	Chr	Position	Alleles	MAF	Gene	Location	Meta P-value	Arizona		Oklahoma		North/South Dakotas	
								Beta	P-value	Beta	P-value	Beta	P-value
rs3740394	10	104634474	A/G	0.19	AS3MT	intron	2.83e-38	5.37	1.29e-11	6.00	3.60e-14	5.57	1.14e-15
rs11191439	10	104638723	T/C	0.19	AS3MT	coding	1.12e-36	5.48	7.27e-12	5.74	5.02e-13	5.44	6.04e-15
rs4919694	10	104698978	T/C	0.19	CNNM2	intron	9.67e-36	5.42	1.17e-11	5.58	1.91e-12	5.41	8.49e-15
rs3740390	10	104638480	C/T	0.20	AS3MT	intron	6.50e-34	-6.00	6.24e-15	-4.82	6.94e-12	-5.06	9.04e-11
rs11191453	10	104659852	T/C	0.20	AS3MT	intron	1.80e-32	-5.70	2.77e-13	-4.81	7.77e-12	-5.06	9.04e-11
rs7911488	10	105154089	A/G	0.26	USMG5	UTR	3.57e-31	-5.34	7.35e-12	-4.33	4.21e-12	-4.20	1.65e-10
rs4925	10	106022789	C/A	0.13	GSTO1	coding	4.60e-6	-4.31	5.95e-4	-2.28	0.01	-1.65	0.04
rs1147611	10	106025258	G/T	0.19	GSTO1	intron	8.45e-6	-2.76	0.01	-1.73	0.02	-1.87	4.54e-3
rs2297235	10	106034491	A/G	0.12	GSTO2	UTR	1.67e-5	-4.32	5.23e-4	-2.08	0.02	-1.43	0.07

Abbreviations: Chr, chromosome. MAF, minor allele frequency. SNP, single nucleotide polymorphism.

All SNP 9 associations are below candidate SNP significance threshold of 9.33e-5 for meta-analysis. Base position according to human genome build 18.

Table S12. Top candidate SNP associations for principal component 2 of arsenic species

SNP	Chr	Position	Alleles	MAF	Gene	Location	Meta P-value	Arizona		Oklahoma		North/South Dakotas	
								Beta	P-value	Beta	P-value	Beta	P-value
rs3740390	10	104638480	C/T	0.20	AS3MT	intron	3.96e-8	0.97	8.11e-4	0.97	8.11e-4	0.97	8.11e-4
rs11191453	10	104659852	T/C	0.20	AS3MT	intron	6.10e-8	0.96	8.88e-4	0.96	8.88e-4	0.96	8.88e-4
rs11191439	10	104638723	T/C	0.19	AS3MT	coding	7.55e-7	-1.18	3.05e-4	-1.18	3.05e-4	-1.18	3.05e-4
rs4919694	10	104698978	T/C	0.19	CNNM2	intron	9.18e-7	-1.17	3.64e-4	-1.17	3.64e-4	-1.17	3.64e-4
rs3740394	10	104634474	A/G	0.19	AS3MT	intron	6.64e-6	-1.09	8.13e-4	-1.09	8.13e-4	-1.09	8.13e-4

Abbreviations: Chr, chromosome. MAF, minor allele frequency. SNP, single nucleotide polymorphism.

All SNP 5 associations are below candidate SNP significance threshold of 9.33e-5 for meta-analysis. Base position according to human genome build 18.

Figure S1. Quantile-quantile plot for MetaboChip of percent arsenic species and principal components of arsenic species

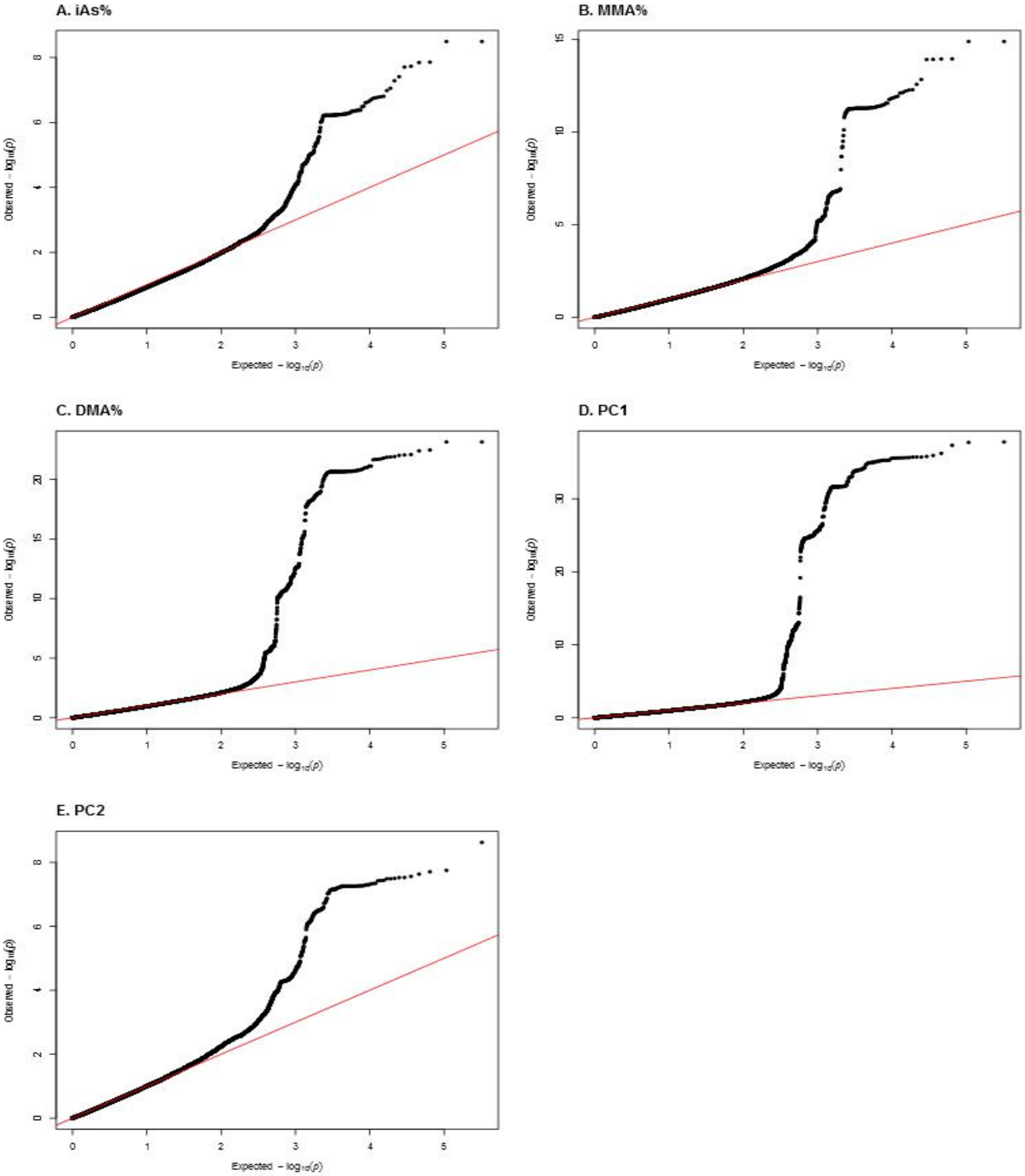

Deviation from expected p-values using MetaboChip for A. percent inorganic arsenic (iAs%), B. percent monomethylarsononate (MMA%), C. percent dimethylarsinate (DMA%), D. principal components 1 (PC1), and E. principal components 2 (PC2).

Figure S2. Quantile-quantile plot for candidate SNPs of percent arsenic species and principal components of arsenic species

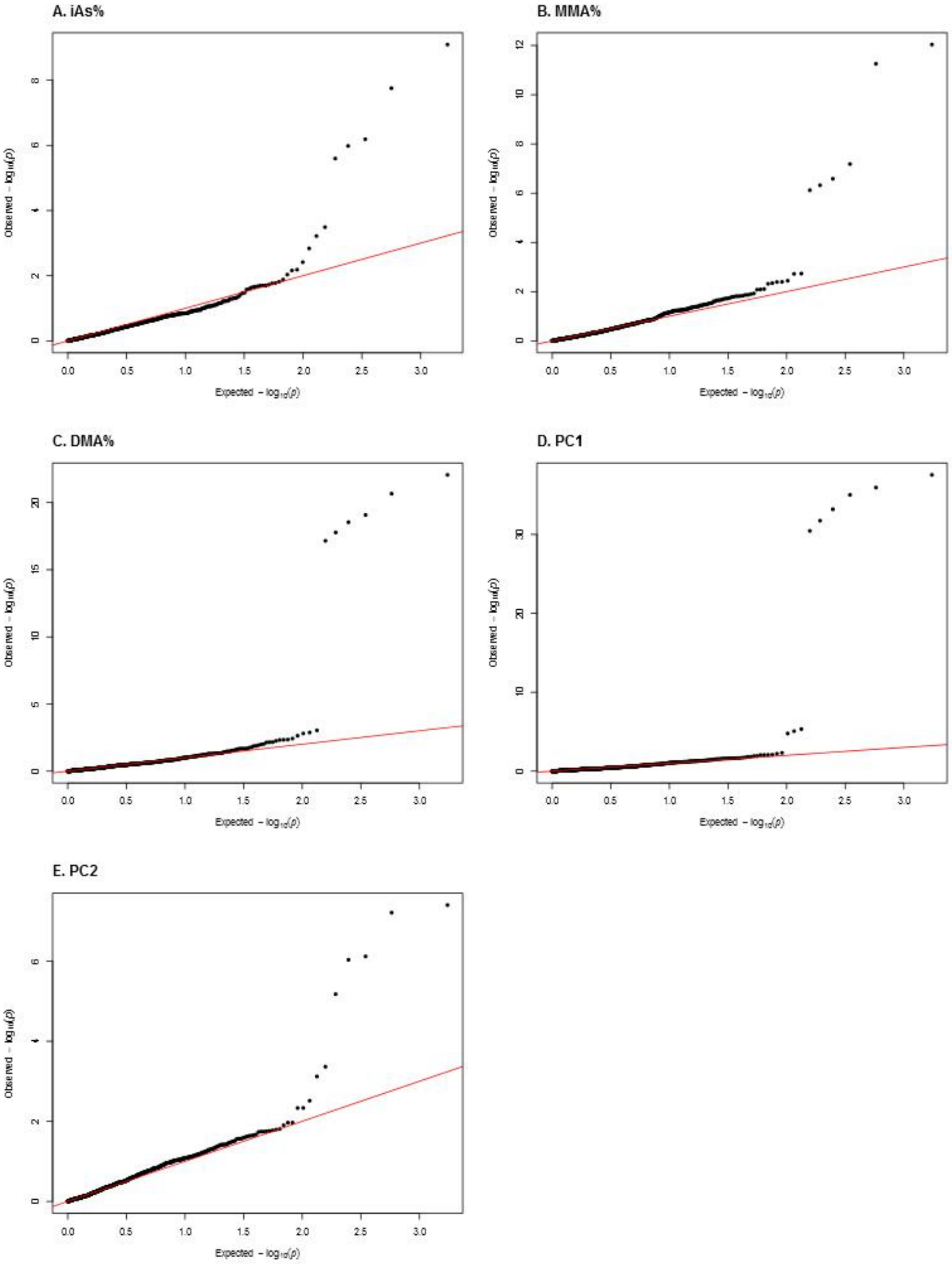

Deviation from expected p-values using MetaboChip for A. percent inorganic arsenic (iAs%), B. percent monomethylarsononate (MMA%), C. percent dimethylarsinate (DMA%), D. principal components 1 (PC1), and E. principal components 2 (PC2)

Figure S3. Manhattan plot of MetaboChip associations for percent inorganic arsenic

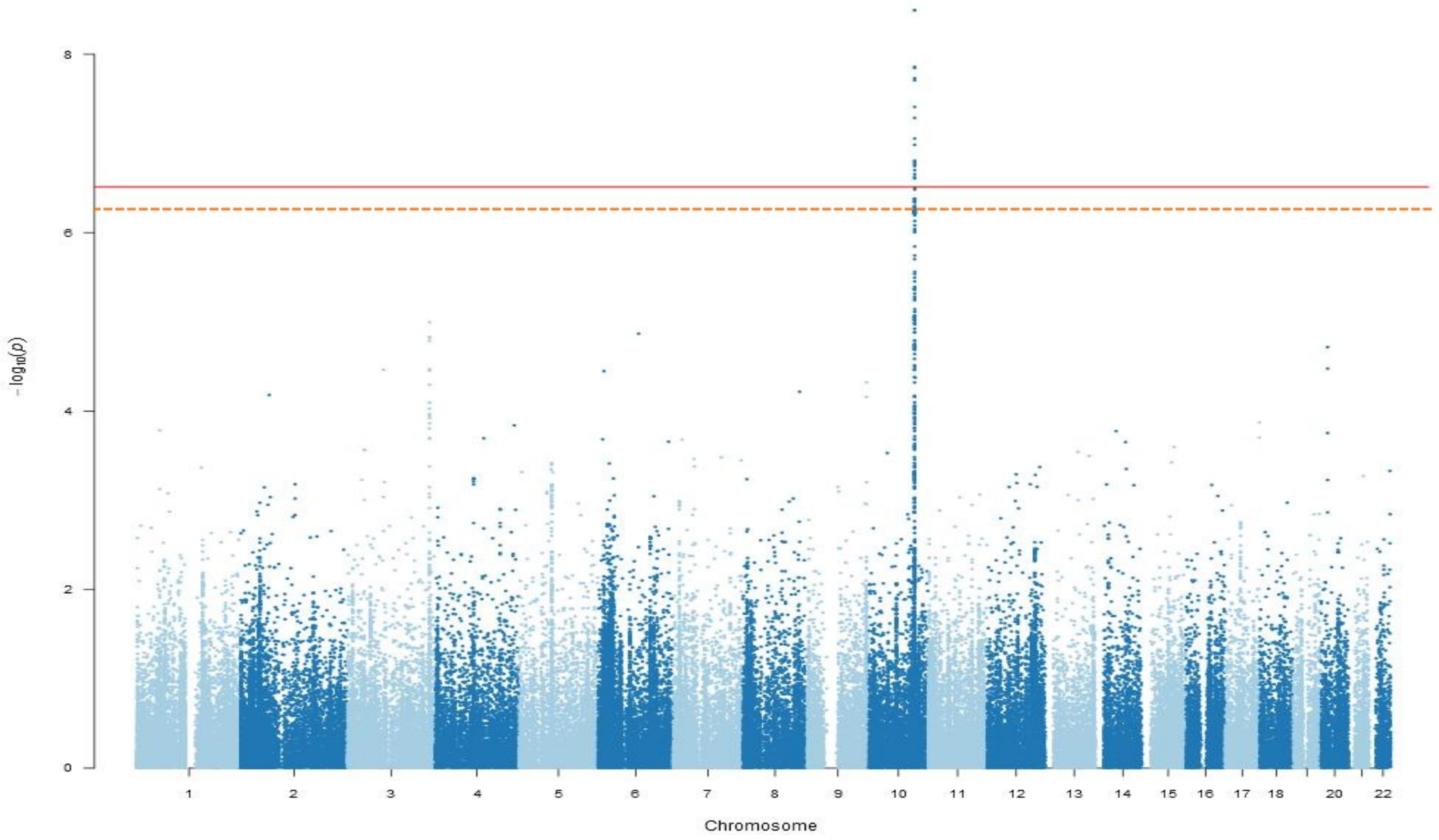

The solid red line is the MetaboChip-wide significance threshold at -log(4.13e-7) or 6.38. The dashed orange line is the suggestive MetaboChip-

Figure S4. Manhattan plot of MetaboChip associations for percent monomethylarsononate

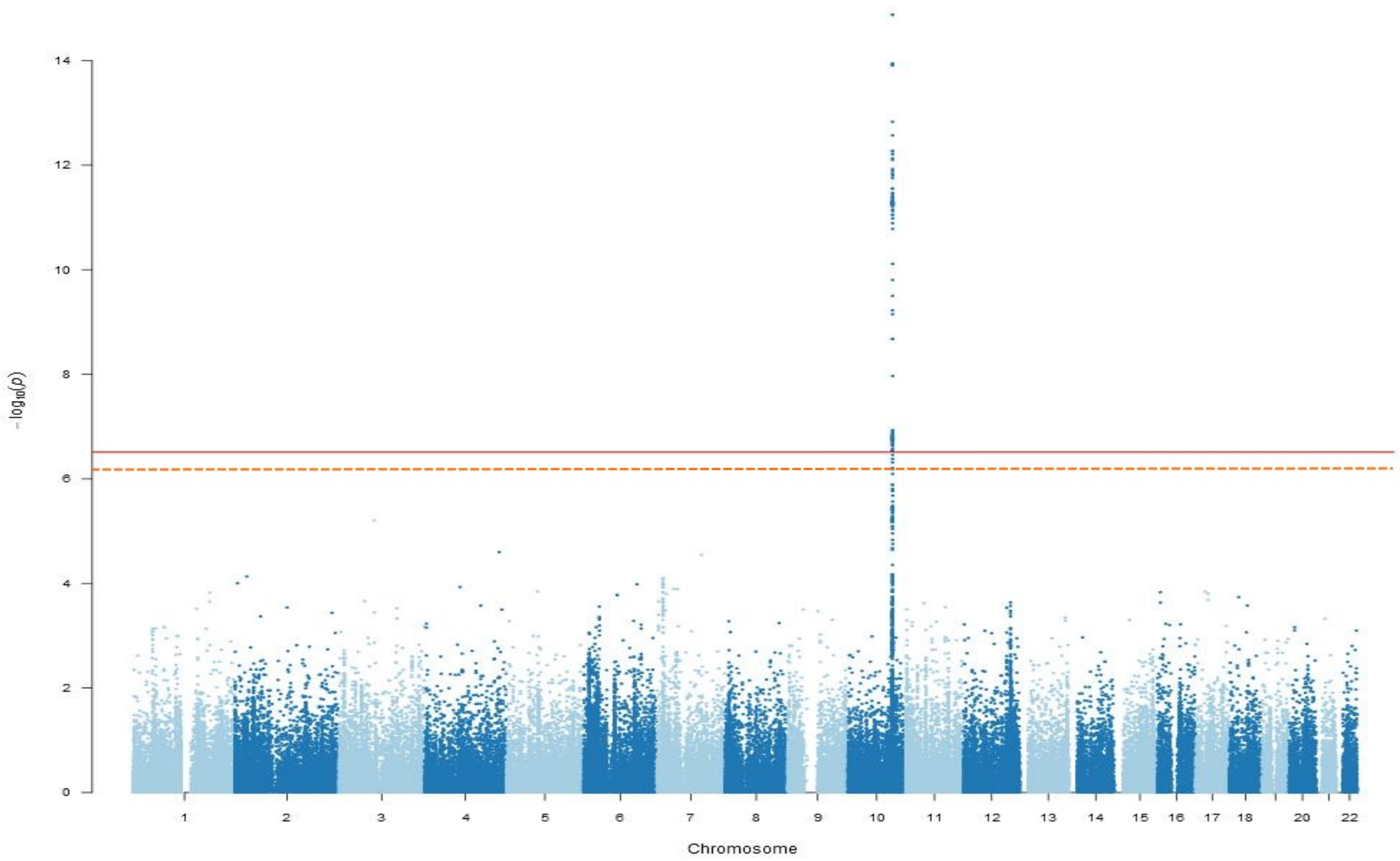

The solid red line is the MetaboChip-wide significance threshold at -log(4.13e-7) or 6.38. The dashed orange line is the suggestive MetaboChip-wide LD threshold at –log(7.77e-7).

Figure S5. Manhattan plot of MetaboChip associations for percent dimethylarsinate

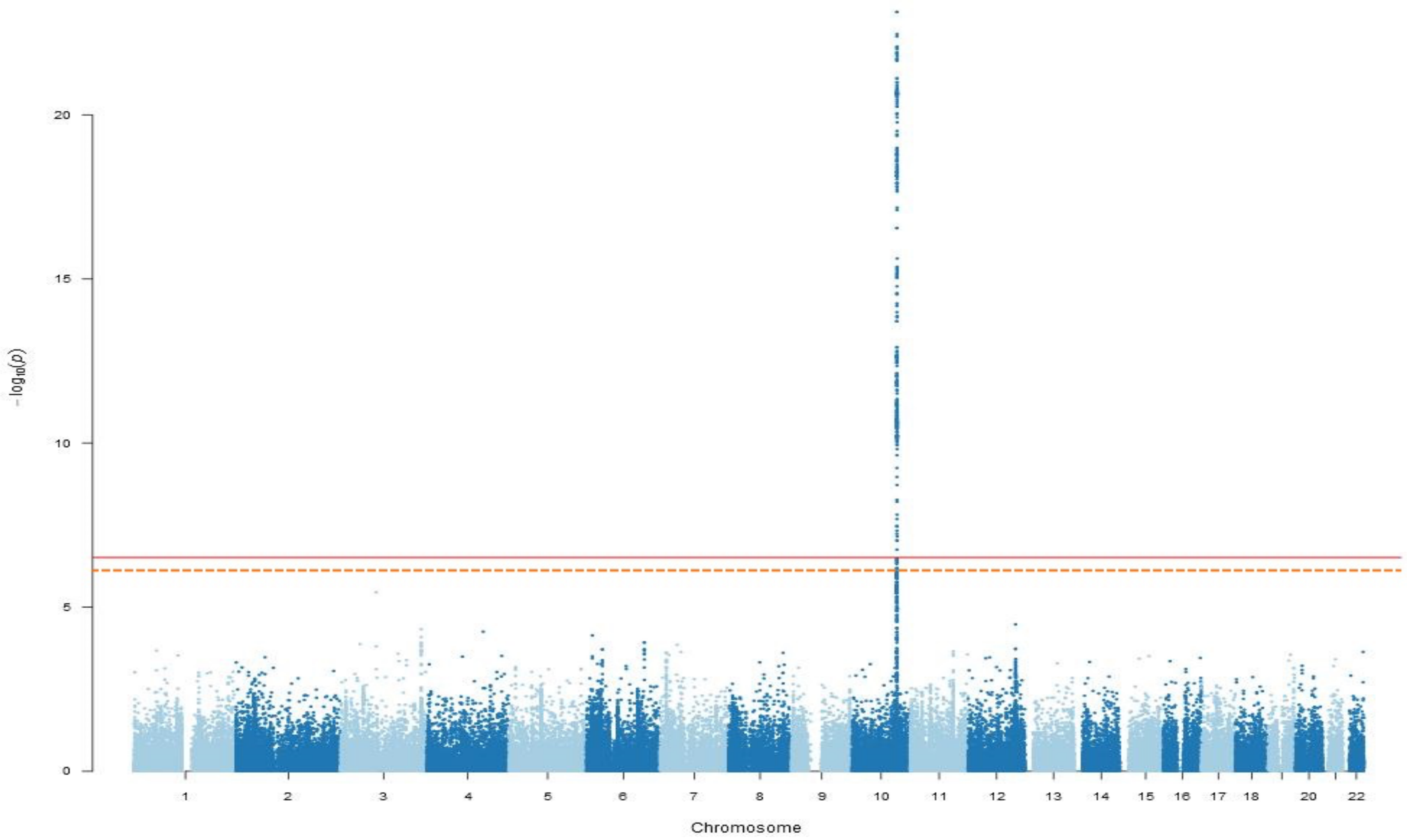

The solid red line is the MetaboChip-wide significance threshold at -log(4.13e-7) or 6.38. The dashed orange line is the suggestive MetaboChip-wide LD threshold at –log(7.77e-7).

Figure S6. Manhattan plot of MetaboChip associations for principal component 1 of arsenic species

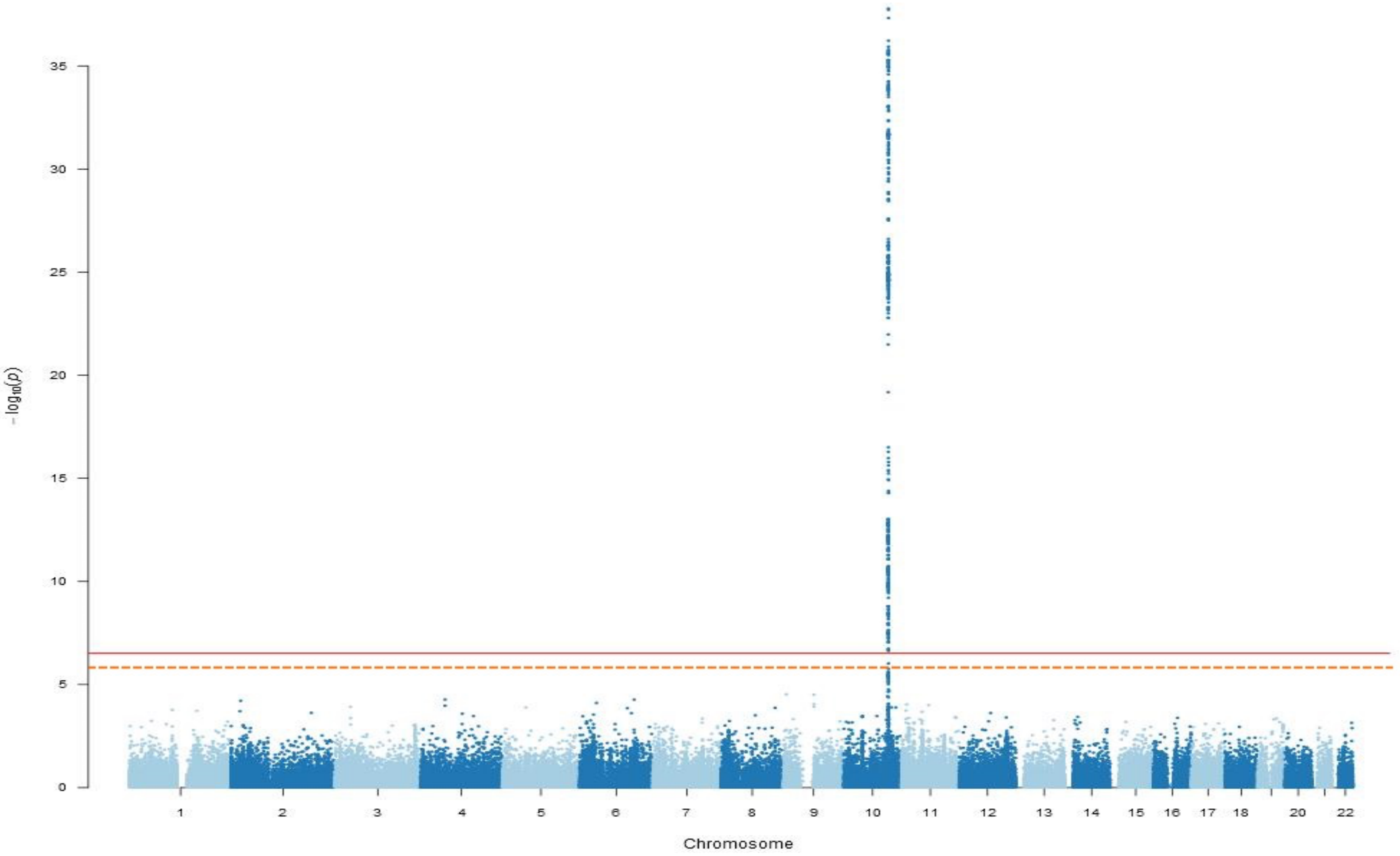

The solid red line is the MetaboChip-wide significance threshold at -log(4.13e-7) or 6.38. The dashed orange line is the suggestive MetaboChip-wide LD threshold at –log(7.77e-7).

Figure S7. Manhattan plot of MetaboChip associations for principal component 2 of arsenic species

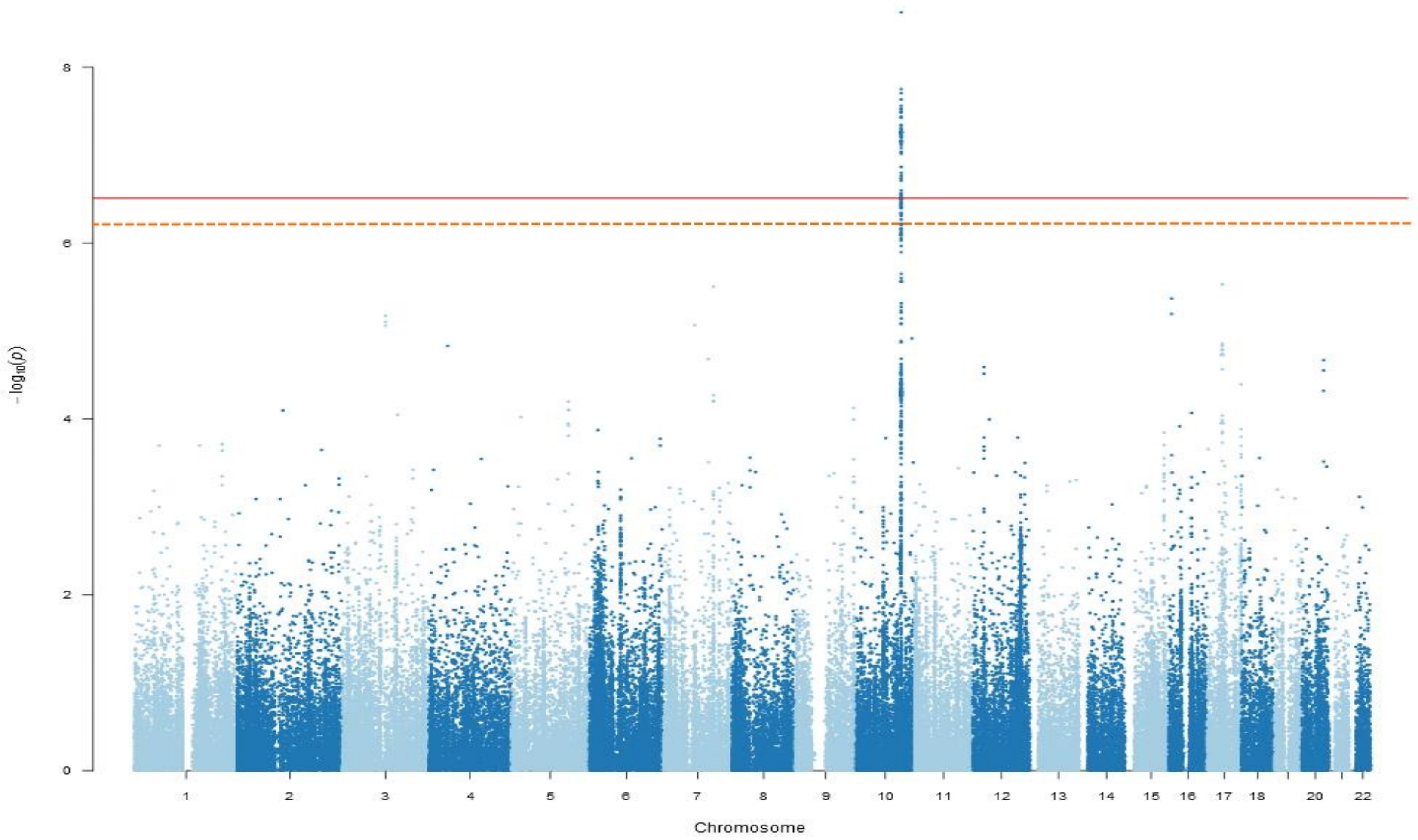

The solid red line is the MetaboChip-wide significance threshold at -log(4.13e-7) or 6.38. The dashed orange line is the suggestive MetaboChip-wide LD threshold at –log(7.77e-7).

Figure S8. Regional association plot at 10q24 of percent arsenic species conditioned on rs12768205

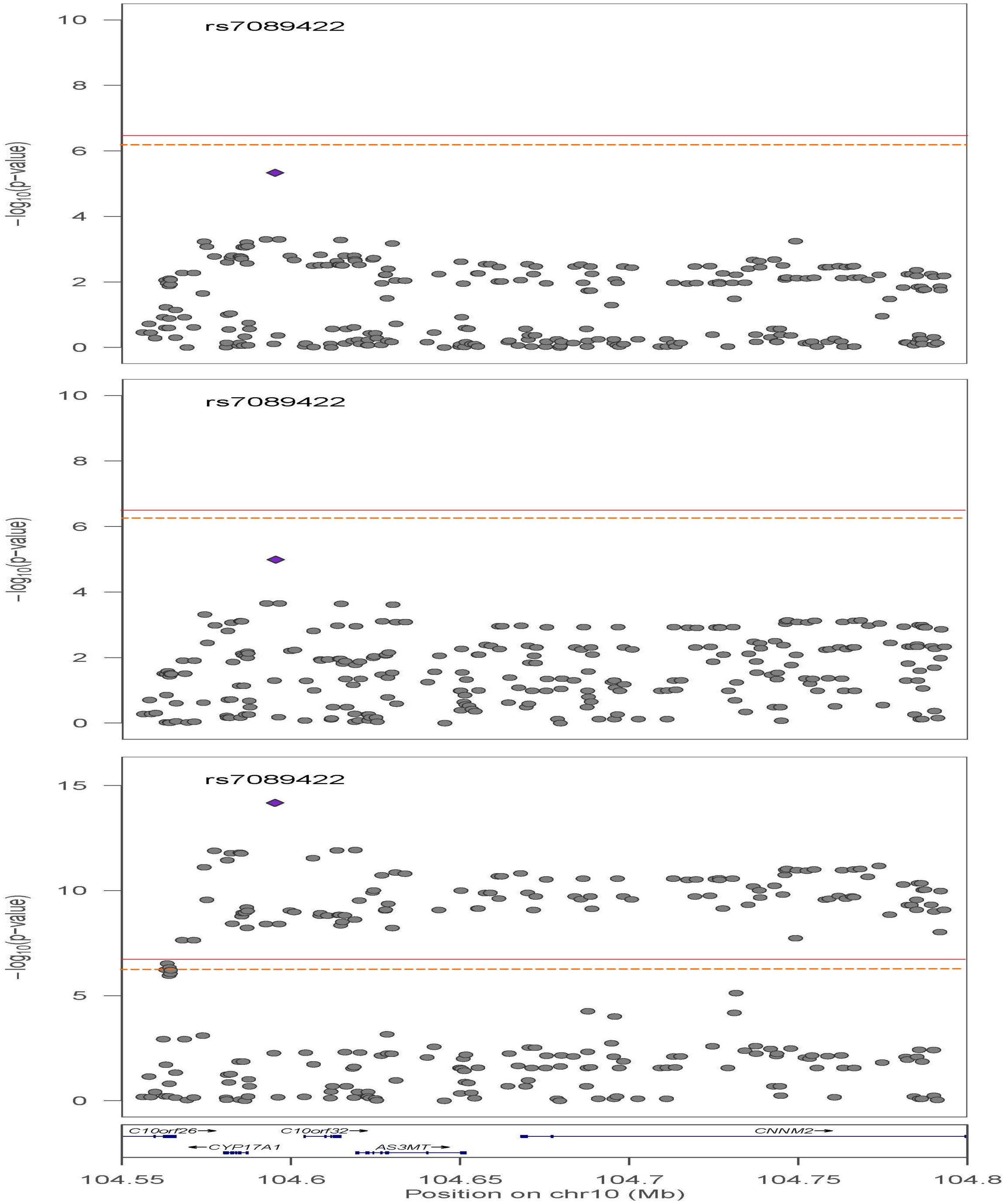

Index SNP rs12768205 nearby associations according to human genome build 18 for percent inorganic arsenic (iAs%) in top panel, percent monomethylarsononate (MMA%) in middle panel, and percent

dimethylarsinate (DMA%) in bottom panel. The solid red line is the MetaboChip-wide significance threshold at -log(4.13e-7) or 6.38. The dashed orange line is the suggestive MetaboChip-wide LD threshold at –log(7.77e-7).

Figure S9. Regional association plot at 10q24 of principal components of arsenic species conditioned on index SNP

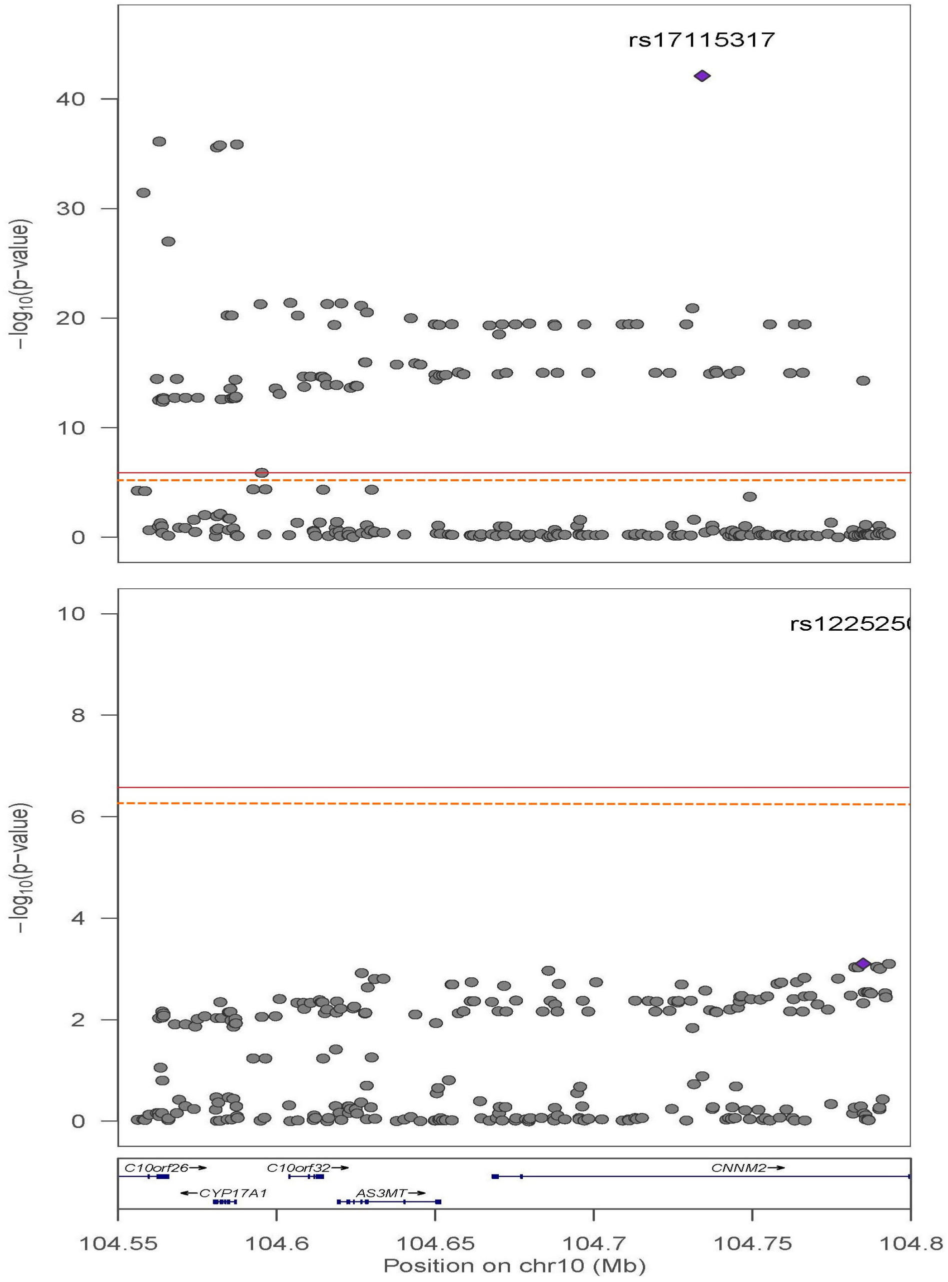

Index SNPs for principal components (PC1 rs3740394 in top panel, PC2 rs7098825 in bottom panel) and nearby associations according to human genome build 18. The solid red line is the MetaboChip-wide significance threshold at -log(4.13e-7) or 6.38. The dashed orange line is the suggestive MetaboChip-wide LD threshold at –log(7.77e-7).

Figure S10. Distribution of percent arsenic species by rs3740394 genotype

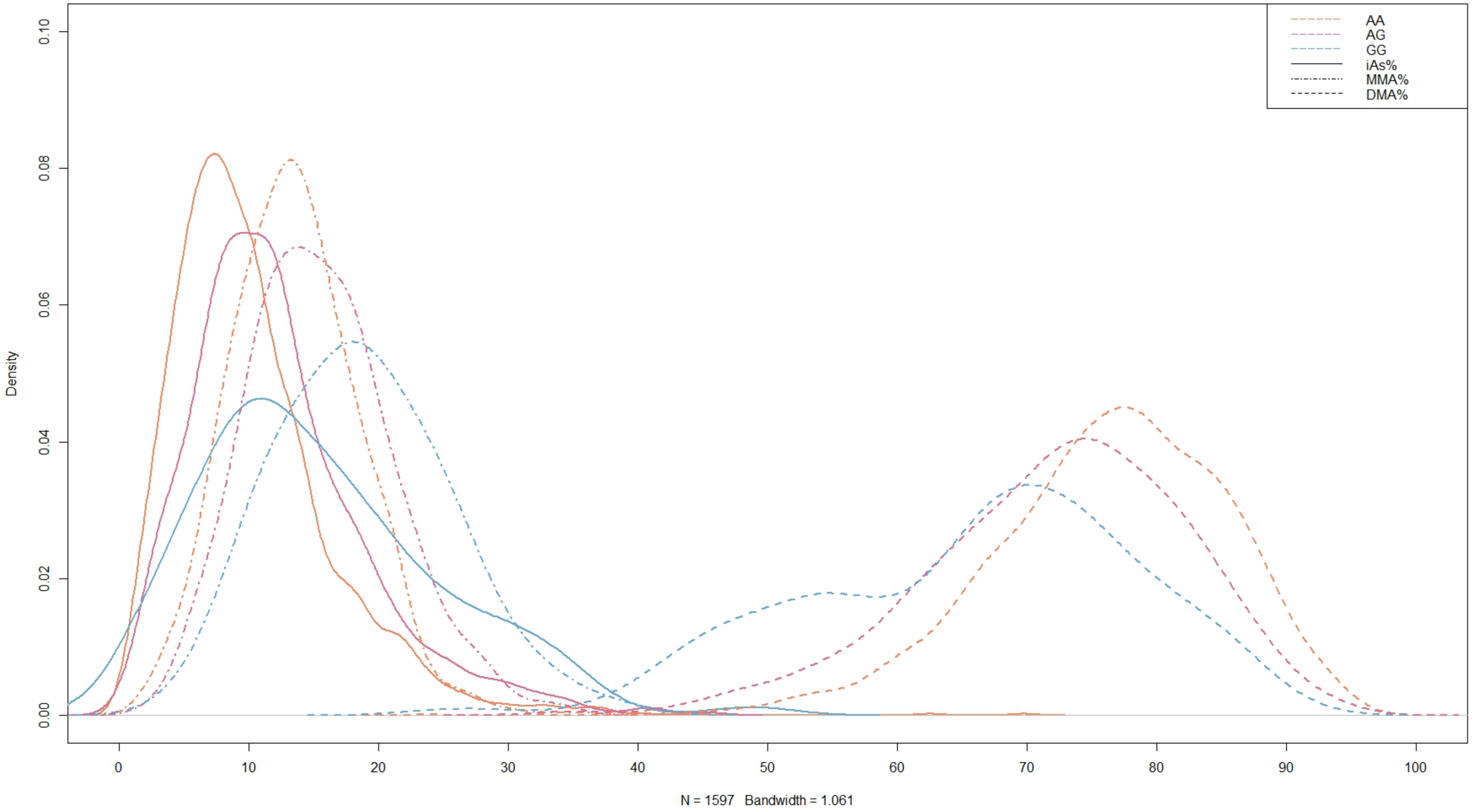

Index SNP rs3740394 for percent arsenic species principal components shows separation of distribution of percent inorganic arsenic (iAs%), percent monomethylarsononate (MMA%) and dimethylarsinate (DMA%) by genotype. Of the 2,428 participants, the distribution of genotypes are homozygous dominant AA (1594), heterozygous AG (732), homozygous recessive GG (102).

Figure S11. Distribution of percent arsenic species by rs7098825 genotype

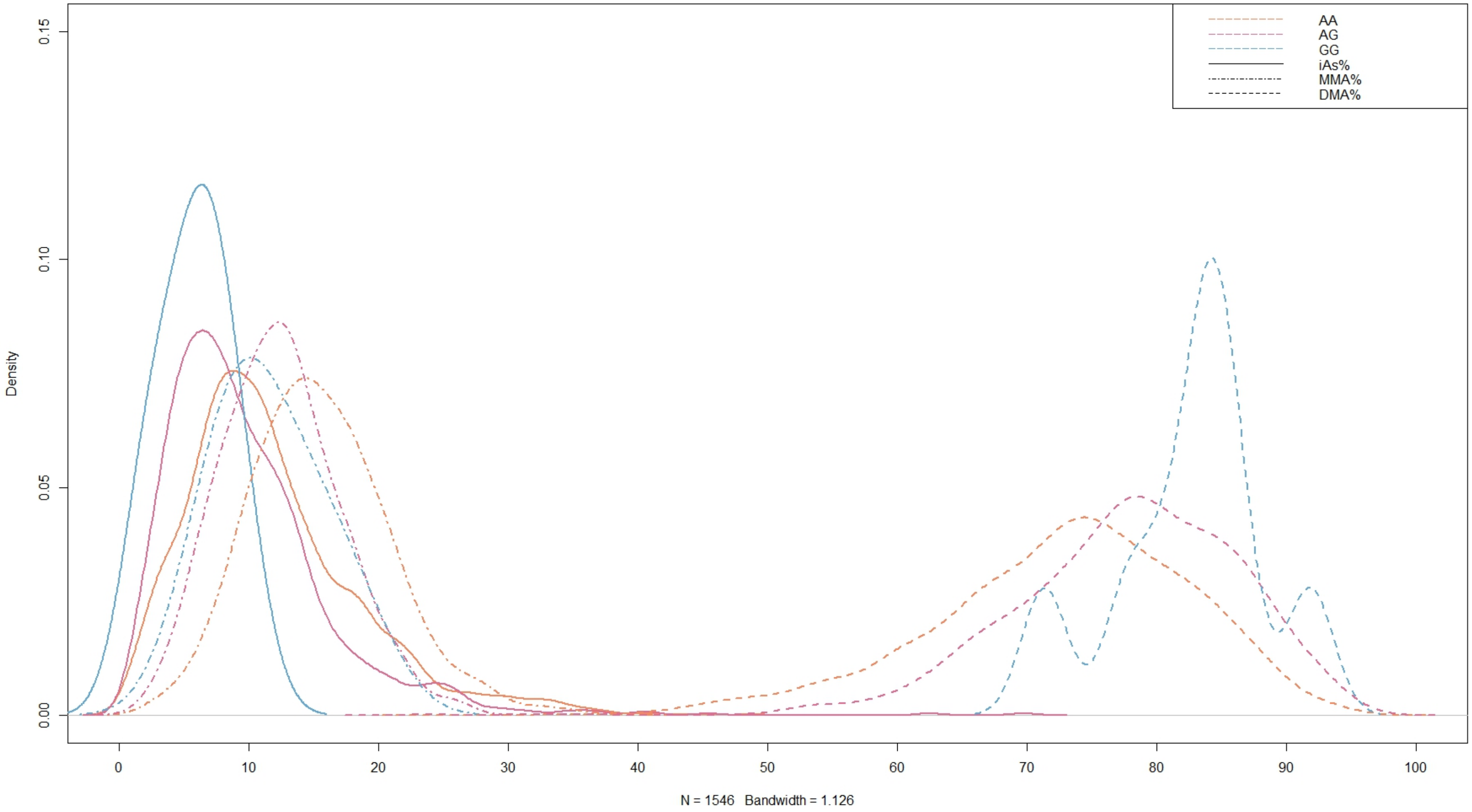

Index SNP rs7098825 for percent arsenic species principal components shows separation of distribution of percent inorganic arsenic (iAs%), percent monomethylarsononate (MMA%) and dimethylarsinate (DMA%) by genotype. Of the 2,398 participants who were polymorphic at rs7098825, the distribution of genotypes are homozygous dominant AA (1543), heterozygous AG (847), homozygous recessive GG (8).
